# Targeting the Spliceosomal Protein USP39 Through Allosteric Ligands and PROTAC‐Induced Degradation

**DOI:** 10.1002/anie.202516809

**Published:** 2025-12-21

**Authors:** Daniel Schäfer, Cristian Prieto‐Garcia, Jianhui Wang, Marcel Heinz, Vigor Matkovic, Pavel Kielkowski, Sebastian Hasselbeck, Varun Jayeshkumar Shah, Stefan Knapp, Gerhard Hummer, Ivan Dikic, Xinlai Cheng

**Affiliations:** ^1^ Buchmann Institute for Molecular Life Sciences Johann Wolfgang Goethe‐University Frankfurt am Main Max‐von‐Laue‐Str. 15 D‐60438 Frankfurt am Main Germany; ^2^ Institute for Pharmaceutical Chemistry Johann Wolfgang Goethe‐University Frankfurt am Main Max‐von‐Laue‐Str. 9 D‐60438 Frankfurt am Main Germany; ^3^ Frankfurt Cancer Institute Paul‐Ehrlich‐Str. 42–44 D‐60596 Frankfurt am Main Germany; ^4^ Mildred‐Scheel‐Nachwuchszentrum (MSNZ) University Cancer Center (UCT) Frankfurt University Hospital Frankfurt am Main Theodor‐Stern‐Kai 7 D‐60596 Frankfurt am Main Germany; ^5^ Institute of Biochemistry II Frankfurt University Hospital Building 75, Faculty of Medicine Frankfurt am Main Theodor‐Stern‐Kai 7 D‐60596 Frankfurt am Main Germany; ^6^ Department of Theoretical Biophysics Max Planck Institute of Biophysics Max‐von‐Laue‐Str. 3 60438 Frankfurt am Main Germany; ^7^ Department of Chemistry Ludwig Maximilian University München Würmtalstrasse 201 81375 Munich Germany; ^8^ Institute of Biophysics Johann Wolfgang Goethe‐University Max‐von‐Laue‐Str. 1 D‐60438 Frankfurt am Main Germany

**Keywords:** Biological chemistry and chemical biology, Drug discovery, PROTAC, Spliceosome, USP39

## Abstract

The precise regulation of gene expression is fundamental to cellular homeostasis and diversity. Dysregulation of splicing has been implicated in a range of diseases, including cancer and neurodegeneration. Ubiquitin‐specific protease 39 (USP39), an essential spliceosome component lacking enzymatic activity, has remained an elusive target for pharmacological intervention. Here, we report the discovery of small‐molecule ligands that selectively engage with USP39 through a thiazole scaffold, primarily interacting with its zinc finger domain. Guided by AlphaFold‐based structure–activity relationship studies, we designed and optimized proteolysis‐targeting chimeras (PROTACs), culminating in the development of USP39_PROTAC_V1, which harnesses the von Hippel–Lindau (VHL) E3 ubiquitin ligase for targeted degradation. Biophysical and biochemical assays demonstrated potent ternary complex formation and nanomolar‐range binding affinities. In cellular models, USP39_PROTACs achieved efficient degradation of USP39 at concentrations as low as 1 nM, with minimal off‐target effects as confirmed by proteome‐wide profiling. Mechanistic studies revealed that degradation was dependent on VHL recruitment and was abrogated by proteasome or neddylation inhibition. Notably, USP39 depletion recapitulated 5′‐splice‐site‐specific splicing patterns previously described, thereby validating both the mechanism of action and the therapeutic relevance of this approach—particularly for modulating splicing‐associated disease pathways such as cancer and retinitis pigmentosa.

## Graphical abstract

Proteolysis‐targeting chimeras (PROTACs) enable degradation of proteins previously considered undruggable by harnessing the cellular ubiquitin–proteasome system. In this study, Schäfer et al. identify thiazole‐based small molecules that allosterically bind the zinc finger domain of ubiquitin‐specific protease 39 (USP39), a non‐enzymatic scaffold protein essential for spliceosome assembly and implicated in proteotoxicity‐relevant diseases. These ligands were converted into PROTACs that effectively recruit E3 ligases, induce potent, selective USP39 degradation at nanomolar concentrations in cells.

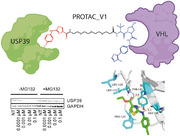

## Introduction

RNA splicing is a fundamental process in eukaryotic cells where introns are removed and exons are joined to form mature mRNA molecules from pre‐mRNA.^[^ ^1^, ^2^ ^]^ Alternative splicing enables a single gene to produce multiple protein variants, thereby significantly enhancing proteome diversity.^[^ ^3^ ^]^ Splicing is crucial for various cellular functions, including differentiation, development, and adaptive responses to environmental stimuli. Dysregulation of this process has been implicated in numerous diseases, such as cancer.^[^ ^4^ ^]^

Although the precise mechanisms linking splicing dysregulation to cancer cell death remain incompletely understood, one critical mechanism could involve proteotoxic stress, which arises from the accumulation of misfolded or unstable proteins resulting from impaired RNA‐splicing fidelity.^[^ ^5^ ^]^ At the core of alternative splicing is the spliceosome, a dynamic RNA‐protein complex adopting two main forms: the major spliceosome, characterized by U2‐type small nuclear ribonucleoproteins (snRNPs), and the minor spliceosome, characterized by U12‐type snRNPs.^[^ ^6^ ^]^ A vital component within these spliceosomes is ubiquitin‐specific protease 39 (USP39), also known as U4/U6.U5 tri‐snRNP‐associated protein 2.^[^ ^6^, ^7^ ^]^

Recent studies have demonstrated that loss or deficiency of USP39 disrupts spliceosome assembly, leading to proteotoxic stress and physiologically relevant cell death in vivo.^[^ ^8^ ^]^ In addition, other reports have provided strong evidence for the critical role of USP39 in cancer development and oncogenesis.^[^ ^9^, ^10^ ^]^ These findings highlight USP39 as a key regulator of spliceosome integrity and suggest that targeting USP39 could represent a promising therapeutic strategy for aggressive cancer.^[^ ^11^ ^]^

Interestingly, USP39 differs from other ubiquitin‐specific proteases because it lacks essential residues for catalytic activity such as the active site histidine and cysteine.^[^ ^12^ ^]^ Recent structural studies using cryo‐electron microscopy provided insights into the critical role of USP39 in spliceosome function.^[^ ^6^, ^13^ ^]^ Specifically, interactions mediated by the zinc‐finger domain of USP39 (USP39‐ZNF) with the U5 snRNP and its USP domain with pre‐mRNA‐processing factor 6 and 8 have been identified as key interactions.^[^ ^6^, ^13^ ^]^ The lack of catalytic activity and the difficulty of targeting USP39‐mediated protein interactions pose challenges to drug development efforts aimed at modulation USP39.

Proteolysis‐targeting chimeras (PROTACs) have recently emerged as innovative and powerful therapeutic tools, particularly effective in targeting proteins previously considered challenging or “undruggable”.^[^ ^2^, ^14^, ^15^, ^16^ ^]^ Unlike traditional small molecule inhibitors, PROTACs do not need to target an enzymatically active site. Instead, they utilize the ubiquitin‐proteasome system to degrade their target proteins.^[^ ^17^ ^]^ Previous studies have highlighted that ligand‐induced proximity can enhance protein–protein interactions by reducing the entropic cost of binding, enabling PROTACs to efficiently degrade target proteins even when the initial ligand‐protein interaction affinity is moderate,^[^ ^18^ ^]^ similar to thalidomide‐ and rapamycin‐based molecular glues.^[^ ^19^, ^20^ ^]^ This feature makes PROTACs particularly valuable for targeting proteins that are devoid of catalytic activity such as USP39. Additionally, PROTACs are often highly specific reducing off‐target activity commonly associated with conventional inhibitors.^[^ ^21^ ^]^

In our study, we employed a chemical screening approach, successfully identifying thiazole‐based compounds capable of binding USP39. By transforming these binders into PROTAC molecules, we achieved potent degradation of USP39 at low nanomolar concentrations, illustrating a potential therapeutic avenue for treating diseases associated with splicing dysregulation.

## Results and Discussion

### Chemical Screening Identified Thiazole‐Containing Compounds as USP39 Ligands

Our previous research demonstrated that a deficiency of USP39 leads to impaired spliceosome assembly and toxic splicing defects at the 5′‐splice sites.^[^ ^8^ ^]^ To investigate whether USP39 can be degraded through the ubiquitin‐proteasome system, we utilized the dTAG system by fusing FKBP12^F36V^ to the N‐terminus of USP39 (Figure SI1A).^[^ ^22^ ^]^ Treatment with dTAG13 resulted in rapid degradation of FKBP12^F36V^‐USP39 within 24 h without affecting endogenous USP39 levels (Figure SI1A), confirming that USP39 can be degraded by the ubiquitin‐proteasome pathway, a prerequisite for developing PROTAC‐based degraders.

**Figure 1:**
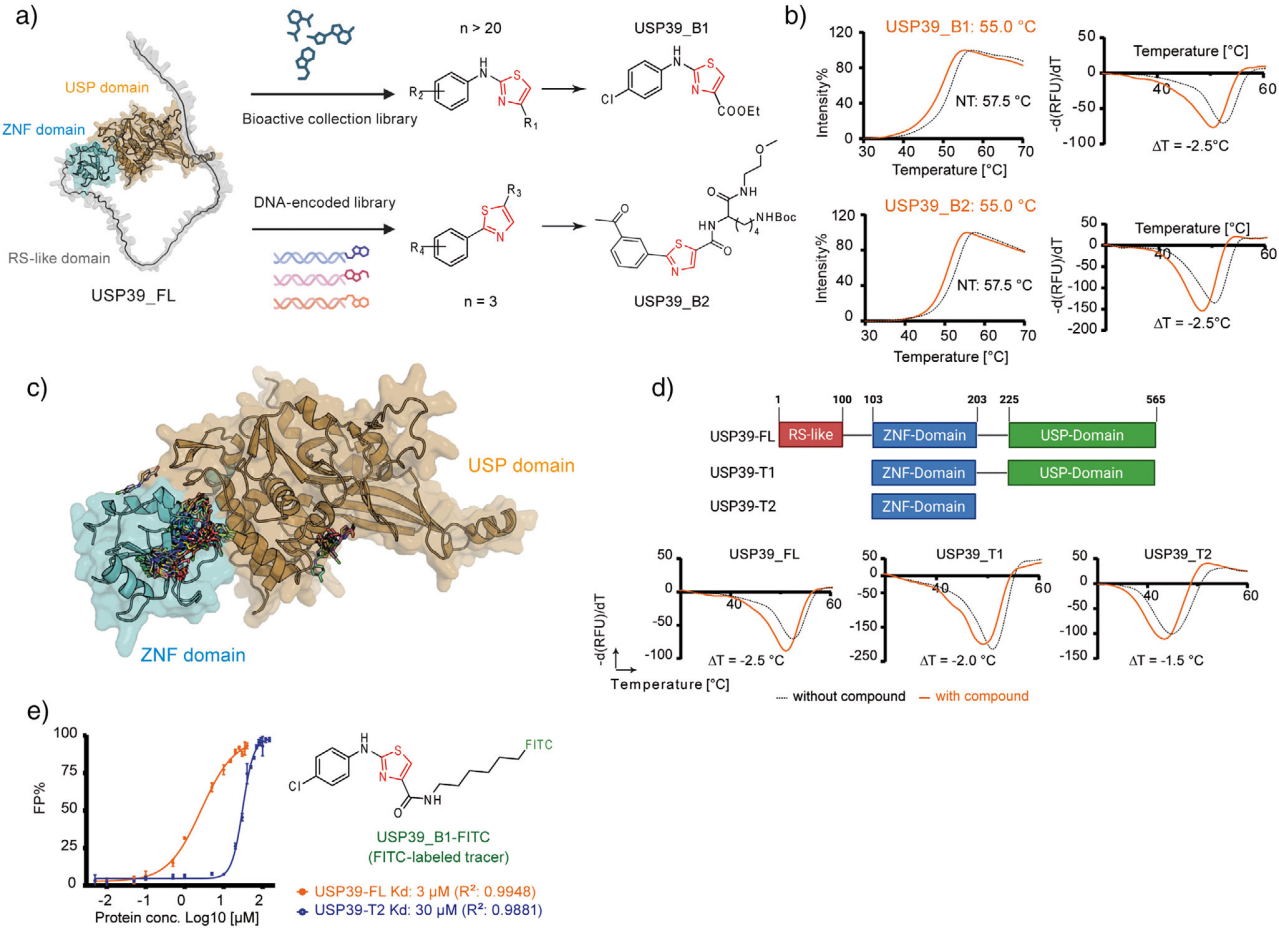
Chemical screening identified thiazole‐containing compounds as USP39 binders. a) AlphaFold3‑predicted structure of full‐length USP39 (USP39_FL) showing three domains: an N‑terminal RS‑like domain (grey), a central zinc‑finger (ZNF) domain (cyan), and a C‑terminal USP domain (gold). Chemical screening of two compound collections yielded two lead ligands USP39_B1 and USP39_B2, each containing a thiazole scaffold. b) Thermal shift assay (TSA) demonstrated compound‐induced decreases in USP39_FL thermal stability (∆*T* = *T*_max_Compound_ − *T*_max___USP39_), confirming direct binding. c) AlphaFold3 predicted USP39_B1 interaction with the ZNF domain (cyan) of truncated USP39 constructs (ZNF + USP domains in gold), highlighting the 100 most probable binding confirmation. d) Domain architecture of the three USP39 expression constructs used for binding studies: USP39_FL, USP39_ZNF_USP (USP39_T1), and USP39_ZNF (USP39_T2), and corresponding ∆*T* values obtained by TSA in the presence of USP39_B1. e) Chemical structure of the FITC‑labeled tracer USP39_B1‑FITC (green). Corresponding non‐logarithmic binding curves confirming saturation at higher concentrations are provided in Figure SI1F. Error bars representing standard deviations from three independent measurements. Curves were fitted using a specific one‐site binding model with a Hill slope parameter. In this context, the Hill slope is used to stabilize curve fitting under tight‐binding FP conditions and does not indicate cooperative binding.

To identify potential USP39 binders, we purified full‐length USP39 protein (USP39_FL, Figure SI1B) and conducted chemical screening using two complementary strategies. First, an in‐house bioactive collection of approximately 200 small molecules, previously assembled from our studies on cellular reprogramming, was screened using thermal shift assay (TSA, Figure 1a).^[^ ^2^, ^23^, ^24^, ^25^, ^26^ ^]^ This focused library was chosen because alterative splicing has been shown to play an important role in epigenetic reprogramming.^[^ ^27^ ^]^ In parallel, a DNA‐encoded library (DEL) screening comprising roughly 600 million unique small molecules was performed. Each DEL compound carries a covalently attached DNA barcode that enables identification after target binding. For this screening, purified USP39_FL protein was immobilized on magnetic beads and incubated with the library; unbound molecules were washed away, and bound DNA‐tagged compounds were eluted. PCR amplification and next‐generation sequencing of the barcode region identified enriched binders, which were subsequently synthesized off‐DNA for biochemical validation. Although multiple chemotypes were recovered from the DEL, only the thiazole series exhibited a coherent structure‐activity relationship and reproducible binding upon off‐DNA resynthesis. Consistently, 20 thiazole‐containing compounds were also identified from the in‐house screen (Figure SI1E), underscoring a convergence between the two orthogonal discovery platforms. Among these, the two most promising candidates from each library, designated USP39_B1 and USP39_B2, were selected for further validation (Figure 1a, Scheme SI1).

TSA confirmed binding by demonstrating decreased thermal stability of USP39_FL in the presence of both compounds (∆*T*_m_: ‐2.5 °C, Figure 1b). The crystal structure of human USP39_FL remains unsolved.^[^ ^28^ ^]^ Structural predictions using AlphaFold 3 indicated that USP39_FL comprises distinct domains, including an unfolded RS‐like domain, a ZNF domain, and a USP domain (Figure 1a,c,d). This architecture closely resembles the truncated spliceosome‐associated derivate USP39_T1 (ZNF and USP domains, Figure 1d) described by Bai et al. and Zhang et al.^[^ ^6^, ^13^ ^]^ They demonstrated that USP39_T1 is essential for spliceosome assembly. Molecular modeling further predicted with high confidence (96%), that USP39_B1 interacted preferably with the ZNF domain (Figure 1c).

To experimentally confirm these interactions, we performed TSA using purified truncated USP39_T1 and USP39_T2 (ZNF domain only), as shown in Figure 1d (Figure SI1C,D). Both protein variants showed reduced thermal stability when treated with USP39_B1, supporting the in silico predicted binding to the ZNF domain (Figure 1c). Fluorescence polarization (FP) assays using USP39_B1 conjugated to fluorescein isothiocyanate (USP39_B1‐FITC, Figure 1e and Scheme SI2) as a FITC‐labeled tracer yielded a K_D_ of 3 µM for USP39_FL (Figure 1e). A reduced binding affinity of 30 µM was observed for USP39_T2, indicating that USP39_B1 primarily binds the ZNF domain, and the USP and RS‐like domain might also contribute to the interaction (Figures 1e and SI1F).

### AlphaFold3‐Guided Compound‐USP39 Interaction Study

**Figure 2:**
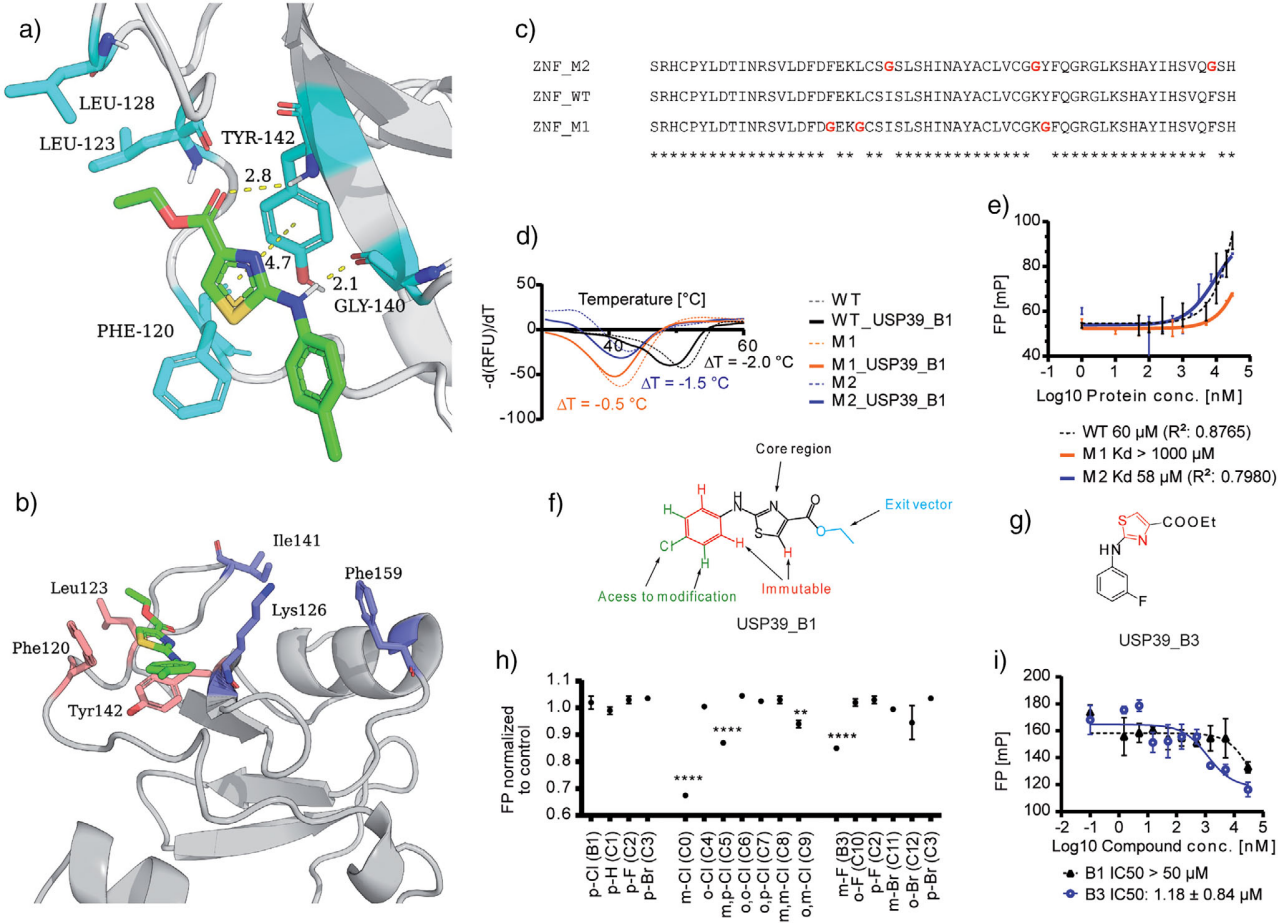
AlphaFold3‐guided compound‐USP39 interaction study. a) Molecular model of USP39_T2 (gray ribbon) bound to USP39_B1 (green sticks), showing key hydrogen bonds and π–π stacking (cyan residue). b) Expanded view of the ZNF binding site highlighting hydrophobic interactions with Phe120, Lys123, and Tyr142 (pink sticks), while Lys128, Ile141, and Phe159 (blue sticks) do not participate directly. c) Sequence alignment of ZNF_WT and two mutants (ZNF_M1 and ZNF_M2) with substituted residues shown in red. d) TSA of ZNF_WT, ZNF_M1, and ZNF_M2 in the absence (dashed lines) and presence (solid lines) of USP39‐B1. E) FP titrations of USP39_B1‐FITC with ZNF_WT, ZNF_M1, and ZNF_M2. f) Chemical structure of USP39_B1 showing the 2‐aminothiazole core (immutable, red), the chlorophenyl modification site (modifiable, green), and the ethyl ester exit vector (blue). g) Chemical structure of the optimized derivative USP39_B3. h) Competitive FP screening of phenyl‐ring analogs at 5 µM normalized to USP39_B1 binding (**, *p* < 0.01; ****, *p* < 0.0001). i) Competitive FP assay comparing USP39_B1‐FITC binding to USP39_B1 and USP39_B3 in the presence of USP39_T2.

USP39_B1 comprises a thiazole moiety and a phenyl motif connected by a secondary amine.^[^ ^23^ ^]^ AlphaFold3 modeling was used for molecular docking studies against the ZNF domain of USP39. While the docking scores showed variability, limiting confident binding pose prediction, key interactions were consistently observed across high‐scoring models. Specifically, the thiazole motif appeared to form two hydrogen bonds with the protein backbone carbonyl of Tyr142 (CO···HN_Tyr142_) and Gly140 (NH···OC_Gly140_), and a π–π stacking interaction between thiazole and the phenyl ring of Tyr142 (Figure 2a, Table SI1–SI3). Additionally, a hydrophobic pocket formed by the side chains of Phe120, Leu123, and Leu128 is predicted to accommodates the ethyl group of the ligand ethyl ester.

To validate the AI‐predicted interaction model, we introduced point mutations into residues predicted to be implicated in ligand binding. A triple mutant, Phe120, Leu123, and Tyr142, was generated (ZNF_M1). For comparison, a control triple mutant (ZNF‐M2) was constructed by substituting three spatially adjacent, but according to our docking data irrelevant residues for ligand binding, Lys126, Ile141, and Phe159. (Figure 2b,c).

TSA revealed that the ZNF_M1 variant abolished ligand‐induced destabilization (Δ*T*_m_ = –0.5 °C), indicating a loss of binding. In contrast, the ZNF_M2 variant retained comparable thermal responses to wild‐type USP39 (Δ*T*_m_ = −1.5 °C), suggesting that the mutated residues in M2 were not critical for interaction (Figure 2d). Consistent with these results, fluorescence polarization assays showed that the ZNF_M1 mutant failed to bind USP39_B1‐FITC, while both ZNF_M2 and wild‐type constructs displayed comparable K_D_ values (Figure 2e). These findings confirm the critical role of Phe120, Leu123, and Tyr142 in mediating compound binding and provide mechanistic validation of the docking model.

In agreement with our screening results, the docking model suggested that the thiazole moiety was the key interacting motif with USP39 and that the carboxylic acid ethyl ester attached to the thiazole ring can serve as a suitable exit vector for conjugation (Figure 2f). To systematically investigate structure–activity relationships, we synthesized a series of derivatives by modifying the phenyl ring, either by substituting the chlorine atom with other halogens or varying the substitution pattern (Figure SI2B). In a competitive binding assay performed at 5 µM compound concentration, derivatives with meta‐F (USP39_B3, Figure 2g) and meta‐Cl substituents showed significantly improved binding affinity compared to the original USP39_B1 (Figure 2h).

Follow‐up titration experiments confirmed this enhancement, with USP39_B3 displaying an IC_50_ value of 1.18 µM against USP39_T2 in the presence of the tracer (USP39_B1‐FITC). In contrast, a weak inhibitory effect was observed in the presence of USP39_B1 at 50 µM (Figure 2i). Compounds bearing one or two chlorine atoms in the ortho position failed to show measurable binding (Figure SI2A; C4, C6), consistent with docking predictions suggesting that ortho‐Cl substituents sterically interfere with the thiazole sulfur, thereby preventing the compound from adopting the required conformation for productive interaction with the protein. Conversely, Cl and F at the meta position were well tolerated and did not induce steric hindrance. Additionally, the introduction of a second Cl (Figure SI2A; C6 – C9) further decreased binding affinity, in line with computational modeling predictions that the binding pocket on the ZNF domain surface cannot accommodate bulky substituents. Finally, replacing Cl with alternative functional groups, such as methyl, methoxy, or cyano, did not lead to any improvement in binding affinity (Figure SI2B).

### Design and in Vitro Characterization of USP39‐targeting PROTACs

**Figure 3:**
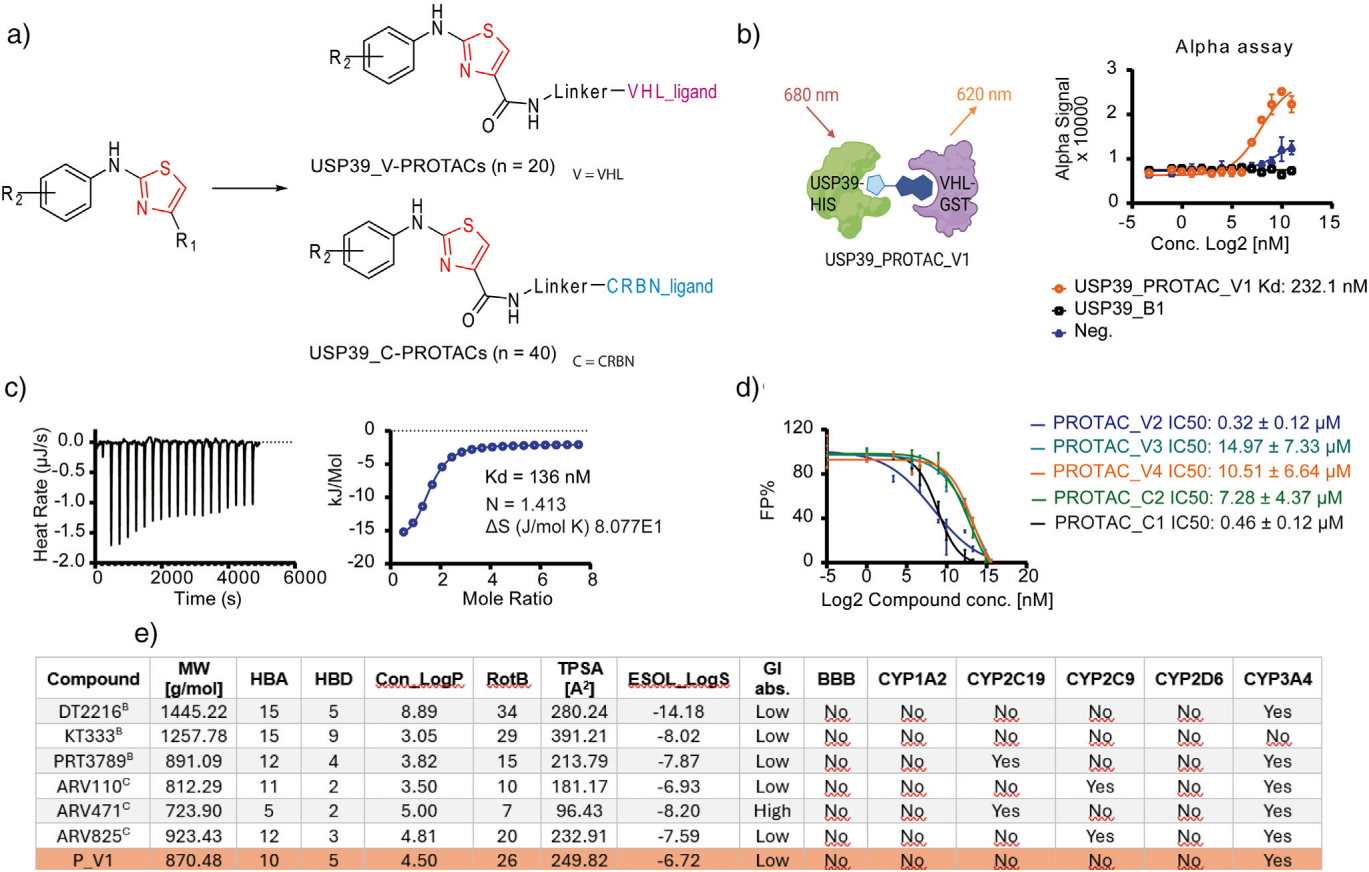
Design and in vitro characterization of USP39‐targeting PROTACs. a) Synthetic strategy for PROTAC synthesis. Thiazole‐based USP39 binders were conjugated via diverse linkers architectures to either a VHL ligand (USP39_V‐PROTACs, *n* = 20) or a CRBN ligand (USP39_C‐PROTACs, *n* = 40). b) Alpha assay‐based ternary complex formation assessment with His‐tagged USP39 and GST‐tagged VHL in the presence of USP39_PROTAC_V1 (orange) or parent binder USP39_B1 (black). c) ITC confirming direct binding between USP39_PROTAC_V1 and USP39_T2. d) Competitive FP assays comparing selected VHL‐ and CRBN‐based PROTAC derivatives. USP39_B1‐FITC served as the tracer, while USP39_T2 acted as the protein. The results were obtained from two independent experiments, each performed in triplicates and are presented as mean ± SD. e) Predicted results from AI‐Based DMPK analysis. MW: Molecular weight; HBA: H‐Bond acceptors; HBD: H‐Bond donors; Con_LogP: Consensus LogP; RotB: Rotatable Bonds; TPSA: Topological polar surface area; ESOL_LogS: predicted aqueous solubility (Log10 mol L^−1^); GI: Gastrointestinal absorption; BBB: Blood–brain barrier. A comprehensive overview of all PROTACs and related data is available in Table SI5 of the Supporting Information.

**Scheme 1:**
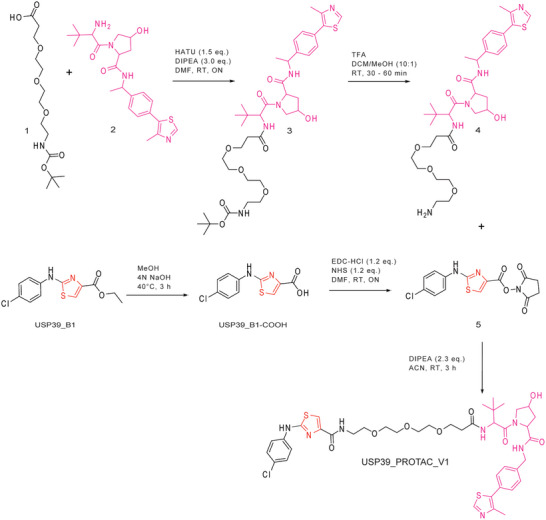
Schematic representation of the chemical synthesis of PROTACs, exemplified by the synthesis of the VHL‐based PROTAC USP39_PROTAC_V1, featuring a PEG3 linker designed to enhance solubility and cellular permeability.

Based on computational structural modeling, we hypothesize that USP39_B1 binds to engage USP39 through shallow, surface‐level interactions rather than occupying a deep, hydrophobic pocket. As expected, we did not observe clear cellular responses upon treatment with the free compounds. Therefore, to enable cellular activity through induced protein degradation, we employed these USP39 ligands as warheads for PROTAC design. Among E3 ligases, CRBN and VHL are the most commonly utilized in PROTAC development.^[^ ^2^, ^15^, ^29^, ^30^ ^]^ Given the crucial role of the linker in mediating ubiquitin transfer from the E3 ligase to the protein of interest (POI),^[^ ^31^ ^]^ we synthesized a series of PROTACs (20 based on VHL and 40 on CRBN) with diverse linkers (Figure 3a, Table SI4). One notable prototype, USP39_PROTAC_V1, incorporated a PEG3 linker to enhance water solubility,^[^ ^31^ ^]^ and exhibited a thermal shift in USP39_FL comparable to that induced by USP39_B1 (Scheme 1, Figure SI3A). All PROTACs were synthesized via standard amidation reactions between amine and carboxylic acid moieties, activated by HATU or NHS esters, under mild conditions (Scheme 1).

The Alpha assay evaluating ternary complex formation between His‐tagged USP39 and GST‐tagged VHL demonstrated a strong binding affinity for USP39_PROTAC_V1 (*K*_D_ = 232 nM), whereas no clear interaction was observed with USP39_B1 (Figure 3b).^[^ ^15^, ^32^ ^]^ Isothermal titration calorimetry (ITC) confirmed robust binding between USP39_PROTAC_V1 and USP39_T2 (*K*_D_ = 136 nM, Figure 3c). In contrast, ITC measurements of the ligand USP39_B1 did not yield a clear binding isotherm. Similar observations have been reported previously for compounds exhibiting moderate affinities toward protein lacking well‐defined hydrophobic binding pockets, where ligand engagement occurs primarily at surface‐exposed regions rather than within structured cavities.^[^ ^33^, ^34^, ^35^ ^]^ Competitive FP assays confirmed that USP39_PROTAC_V2 and USP39_PROTAC_C1 containing meta‐Cl substituents (Figure 2b), exhibited stronger binding with *K*_D_ values of 0.32 µM and 0.46 µM, respectively (Figure 3d, Table SI4).

Physicochemical properties critically influence pharmacokinetic and pharmacodynamic (PK/PD) behavior of small molecules and bifunctional degraders, particularly PROTACs.^[^ ^36^ ^]^ While classical small‐molecule optimization follows the Lipinski “Rule‐of‐Five” (Ro5), beyond‐Ro5 (bRo5) modalities like PROTACs pose additional challenges related to cell permeability, bioavailability, and formulation.^[^ ^37^ ^]^ To systematically assess these parameters for further cell‐based validation, we evaluated our VHL‐ and CRBN‐based PROTACs using SwissADME (Figure 3e, Table SI5). For benchmarking, we included six clinically advanced PROTACs, three VHL‐based (DT2216, KT333, PRT3789) and three CRBN‐based (ARV110, ARV471, ARV825), as we reported recently.^[^ ^36^ ^]^

Among all compounds evaluated, USP39_PROTAC_V1 (P_V1) displayed a balanced physicochemical profile that positions it advantageously within the optimal space for cell‐permeable and pharmacologically tractable PROTACs. Its moderate molecular weight (870.5 g mol^−1^) and intermediate polar surface area (TPSA = 249.8 Å^2^) fall within the range reported for several orally or parenterally active degraders (e.g., PRT3789 = 891 g mol^−1^, 213.8 Å^2^), but are substantially lower than those of bulkier VHL‐based degraders such as DT2216 or KT333, which often suffer from limited permeability.^[^ ^38^ ^]^ The consensus LogP of 4.50 indicates a favorable hydrophilic–lipophilic balance, supporting sufficient solubility while maintaining membrane permeability. In line with this, predicted solubility (ESOL LogS = −6.72) and 26 rotatable bonds reflect a flexible yet synthetically accessible structure with physicochemical properties comparable to those of clinically validated PROTACs. Importantly, P_V1 showed no predicted inhibition of CYP1A2, CYP2C19, or CYP2C9, minimizing potential metabolic liabilities, while its moderate interaction with CYP3A4 remained consistent with other advanced degraders such as ARV‐110 and PRT‐3789. Low predicted GI absorption and lack of BBB permeability are also in line with PROTACs designed for peripheral rather than central targets.

Taken together, USP39_PROTAC_V1 combines compact molecular architecture, balanced lipophilicity, and low predicted off‐target metabolism—traits expected to enhance cellular uptake and metabolic stability. These favorable physicochemical and ADME properties similar to other clinically advanced VHL‐based PROTACs justified its selection as the lead candidate for subsequent cellular degradation and mechanistic studies.

### Effective and Specific Cellular Degradation of USP39 by USP39‐PROTACs

**Figure 4:**
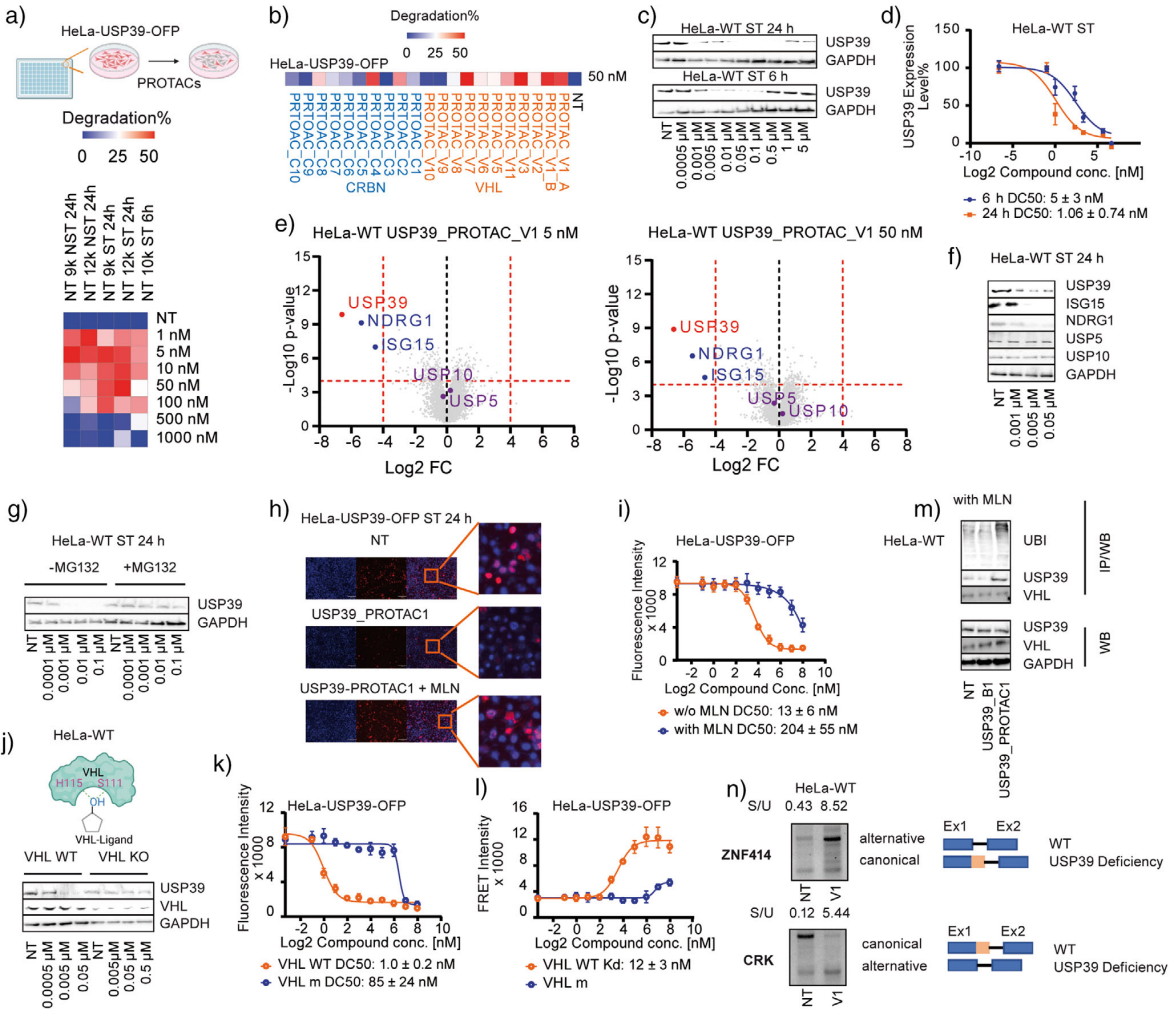
Effective and selective degradation of USP39 in cells by USP39‐PROTACs. a) Optimization of cellular degradation assay in HeLa cells transiently overexpressing USP39‑OFP (HeLa‐USP39‐OFP). Heatmap shows percent USP39 degradation under varying conditions: initial cell densities, serum starvation (ST), treatment durations (6 h, 24 h), and compound concentrations. b) Heatmap summarizing degradation efficacy of CRBN‑based (blue) and VHL‑based (orange) PROTACs at 50 nM in HeLa‑USP39‑OFP cells. c) Representative western blot of USP39 levels in HeLa‐WT cells after 6 h and 24 h treatment with USP39_PROTAC_V1 at indicated concentrations; GAPDH served as a loading control. d) Quantified results from (C), showing relative USP39 protein levels normalized to GAPDH. e) Volcano plot from proteome‐wide mass spectrometry comparing protein levels of HeLa‐WT cells treated with 5 nM (left) and 50 nM (right) USP39_PROTAC_V1 treated, and DMSO control (24 h). *p* < 0.0001, log2Fold‐change (FC) < −4; Red: USP39; Blue: NDRG1 and ISG15; Purple: USP5 and USP10. f) Representative Western blot analysis of USP39, USP5, USP10, ISG15, and NDRG1 levels in HeLa‐WT cells following 24 h ST treatment with USP39_PROTAC_V1 at the indicated concentrations. GAPDH was used as a loading control. g) Proteasome dependence of USP39 degradation. HeLa‐WT cells were co‐treated with MG132 (0.25 µM) or DMSO control and USP39_PROTAC_V1 for 24 h ST. h) Representative immunofluorescence images of HeLa‐USP39_OFP cells treated with DMSO (NT), USP39_PROTAC_V1 (10 nM, 24 h), or USP39_PROTAC_V1 + MLN4924 (1 µM), stained for USP39 (red) and nuclei (blue). i) Quantification of USP39 degradation by fluorescence microscopy, showing DC_50_ values of 13 nM without MLN4924 (orange) and 204 nM with MLN4924 (blue) (MLN4924 = 5 uM). j) VHL‐dependence of PROTAC‐induced degradation. Schematic of PROTAC recruitment to VHL via hydroxyl‐proline interactions (His115, Ser111). Western blot comparing USP39 levels in VHL WT and VHL KO HeLa cells treated with USP39_PROTAC_V1. k) Fluorescence assay in HEK293T cells co‐expressing USP39‑OFP and either VHL_WT‑EGFP or VHL_m (Δ91–121). VHL WT in orange and VHL_m in blue. l) FRET assay measuring ternary complex formation in cells with MLN4924; PROTAC_V1 induces complex formation in VHL WT (orange), with minimal signal in VHL_m (blue). m) Co‐immunoprecipitation (IP) of VHL followed by western blot (WB) for ubiquitin (UBI), USP39, and VHL in the presence of MLN4924; PROTAC_V1 treatment enriches USP39 and polyubiquitin. n) RT–PCR analysis of alternative splicing in ZNF414 and CRK transcripts. Comparison of untreated (NT) and USP39_PROTAC_V1–treated (10 nM, 24 h) samples. Percent spliced/unspliced (S/U) ratios are shown, reflecting USP39‐deficiency–associated splicing defects. Data represent at least four independent biological replicates.

To evaluate the degradation efficacy of USP39_PROTACs, we adapted our recently developed cellular fluorescence assay in HeLa cells transiently overexpressing USP39‐OFP (HeLa‐USP39‐OFP).^[^ ^32^ ^]^ Various experimental conditions were tested, including different initial seeding densities, the presence or absence of serum starvation, varying treatment durations, and compound concentration gradients (Figure 4a). Among these, a 16‐h serum starvation followed by a 24‐h treatment provided the most consistent and reproducible degradation of USP39 and was used for further experiments. Under these experimental conditions, a series of CRBN‐ and VHL‐based PROTACs were evaluated in an in‐cell fluorescence assay at a concentration of 50 nM to assess their ability to induce target protein degradation (Figure 4b, Table SI4). Although SwissADME predictions indicated that CRBN‐based PROTACs may possess more favorable pharmacokinetic properties (e.g., solubility and absorption), the majority of USP39–CRBN–PROTACs failed to induce measurable USP39 degradation in cells (Figure 4b). In contrast, several VHL‐based PROTACs, particularly USP39_PROTAC_V1, demonstrated robust degradation activity. These findings suggest that pharmacodynamic factors, such as ternary complex formation and E3 ligase engagement, may play a more decisive role than predicted pharmacokinetic parameters. Future structure‐guided optimization of linker composition and exit vectors will be essential to fully elucidate the determinants of degradation efficiency for USP39‐targeting PROTACs.

A comprehensive analysis across four cell lines, including HeLa, HEK293T, LNCap, and Kelly, demonstrated that USP39 degradation was initiated at concentrations as low as 5 nM following 6 h of treatment and at 1 nM after 24 h of treatment (Figures 4a,c,d and SI3B‐D). Notably, at concentrations above 500 nM, we observed a recovery in USP39 levels, indicative of the “hook‐effect”, a known phenomenon in PROTAC‐treated systems.^[^ ^2^, ^14^ ^]^ To assess the selectivity of USP39 degradation, a proteome‐wide analysis was conducted using LC‐MS/MS based proteomics. As shown in Figure 4e (*p* < 0.0001, log2FC < ‐4), USP39 emerged as the most significantly downregulated protein among over 6000 detectable proteins. Apart from USP39, only NDRG1 and ISG15, both known to be involved in cell cycle regulation and cellular stress response, showed significant downregulation at 5 nM and 50 nM USP39_PROTAC_V1 after 24 h.^[^ ^8^ ^]^ These finding were further validated by western blot (WB) analysis, which confirmed consistent downregulation of USP39, ISG15, and NDRG1. In contrast, the homologous, USP5 and USP10, remained unaffected in both proteomic and immunoblot analyses (Figure 4f).

To confirm that USP39 degradation was proteasome dependent, cells were co‐treated with MG132, a proteasome inhibitor, which markedly suppressed USP39 degradation (Figure 4g). Similarly, MLN4924, a neddylation inhibitor that suppresses the activating neddylation required for cullin RING E3 ligases (e.g., VHL and CRBN), rescued USP39 from PROTAC_V1‐mediated degradation, reducing the DC_50_ value by approximately 20‐fold in immunofluorescence assays using both fluorescence microscopy (Figure 4h) and plate reader quantification at a concentration of 1 µm respectively 5 µm MLN4924 (Figure 4i).

Mechanistically, USP39_PROTAC_V1 recruits the VHL E3 ligase via a ligand containing a hydroxyl‐proline group that interacts with His115 and Ser111 of VHL (Figure 4j).^[^ ^39^ ^]^ As expected, USP39 degradation was significantly attenuated in VHL‐KO HeLa cells treated with USP39_PROTAC_V1 (Figure 4j). In HEK293T cells co‐expressing USP39_OFP and VHL_WT‐EGFP, a DC_50_ of 1 nM was observed. However, this effect was reduced more than 80‐fold in cells expressing a truncated version of VHL variant (VHL_m) lacking the hydroxyl‐proline binding domain (aa: 91–121, Figure 4k).^[^ ^40^ ^]^

A FRET assay conducted in the presence of MLN4924 further validated ternary complex formation in treated cells. PROTAC_V1 induced complex formation with a K_D_ value of 12 nM, while a minimal signal was observed with VHL_m (Figure 4l). Additionally, co‐immunoprecipitation experiments revealed enrichment of USP39 and polymerized ubiquitin from VHL pull‐downs in PROTAC_V1‐treated samples, confirming ternary complex formation and functional ubiquitin transfer (Figure 4m).

Consistent with recent reports by Prieto‐Garcia et al. and Rogalska et al., USP39 depletion enhances the usage of non‐canonical 5′ splice sites.^[^ ^8^, ^41^ ^]^ In USP39‐depleted cells, reduced tri‐snRNP availability and impaired spliceosome maturation diminish the kinetic advantage of canonical 5′ splice sites. This results in a more balanced competition between U1–U2 binding at alternative and canonical sites as previously reported by our groups.^[^ ^8^ ^]^ Thus, we observed consistent usage of non‐canonical 5′ splice sites in USP39‐deficient cells, particularly consistent for specific genes, such as ZNF414 and CRK.^[^ ^8^ ^]^ The usage of cryptic 5′ splice sites led to an elongated exon 1 in ZNF414 and a shortened exon 1 in CRK.^[^ ^8^ ^]^ To further investigate the selectivity of USP39 degraders that we developed, we established an assay to detect canonical and non‐canonical 5′ splice site usage in exon 1 of both genes upon exposure to PROTACs (Figure 4m, Table SI5). Treatment with 10 nM USP39_PROTAC_V1 for 24 h reproduced these cryptic splicing events, highlighting the selectivity of USP39 degraders (Figure 4n). These results demonstrated that USP39 degradation via PROTACs faithfully mimicked the splicing alterations observed in USP39‐depleted cells.^[^ ^8^ ^]^

## Conclusion

Precise spatial proximity control of protein–protein interactions is fundamental to various biological processes, providing both functional specificity and diversity within cellular pathways.^[^ ^42^, ^43^ ^]^ Our identification of the first small‐molecule ligands targeting USP39, a key inducer of proteotoxicity‐relevant diseases, subsequently developed into PROTAC molecules, uses this fundamental biological principle to functionally mimic the native ubiquitin–proteasome regulatory mechanism for USP39 degradation.

Traditional drug discovery paradigms typically focus on developing high‐affinity small molecules targeting well‐defined hydrophobic binding pockets on enzymes or receptor proteins.^[^ ^44^, ^45^, ^46^, ^47^ ^]^ However, proteins lacking conventional enzymatic clefts, such as USP39, present considerable challenges and are frequently labeled “undruggable”.^[^ ^12^, ^48^, ^49^, ^50^, ^51^ ^]^ Recent developments highlight that low to moderate binding affinity may still be sufficient for effective therapeutic outcomes, particularly within the context of PROTAC‐mediated degradation. For example, Chessum et al. successfully developed a PROTAC targeting the non‐catalytic protein Pirin, demonstrating that high affinity was not strictly necessary for effective degradation.^[^ ^52^ ^]^ Similarly, Han et al. revealed that moderate affinities between PROTAC components and target proteins or E3 ligases can still achieve significant degradation.^[^ ^53^ ^]^ Our findings align with these observations. The thiazole‐based ligands identified in our study bind to USP39 primarily via discrete hydrogen bonds and aromatic stacking interactions, rather than deep hydrophobic pocket interactions and notably induce a negative thermal shift. Although ligand‐induced protein destabilization is uncommon—as binding typically stabilizes proteins with well‐defined hydrophobic cavities such as kinases—proteins lacking structured binding pockets, like multi‐domain scaffolding or RNA‐binding proteins, may exhibit the opposite effect. For example, cas9 displays ligand‐induced destabilization and Cimmperman et al. reported a similar observation for PLK1 upon binding to its phosphorylated peptide substrate.^[^ ^54^, ^55^, ^56^ ^]^ In the case of USP39, which lacks a classical catalytic pocket, it is plausible that compound binding perturbs a stabilizing surface loop or alters ZNF domain conformation, thereby reducing overall protein stability. A related phenomenon was reported by Reinhard et al., demonstrating that protein conformational stability can be both positively and negatively modulated even by buffer conditions.^[^ ^57^ ^]^

Taken together, our study demonstrates that chemically induced proximity can effectively target traditionally undruggable proteins like the spliceosome component USP39. The results emphasize the utility of moderate‐affinity ligands in driving efficient and selective protein degradation via PROTAC technology, highlighting the pivotal role of binding dynamics rather than absolute affinity in enabling efficient ternary complex formation. This work expands the landscape of therapeutic strategies for intractable targets and suggests broader applications of induced proximity beyond conventional PROTACs.^[^ ^32^, ^58^, ^59^ ^]^

## Supporting Information

The chemical syntheses, reagents utilized, chemical structures, NMR spectra, LC/MS and MS spectra, chemical and biological methods, and the protein analyses are included as Supporting Information. The authors have provided additional references in the Supporting Information, which have been included in the manuscript with consecutive numbering.^[^ ^60^, ^61^, ^62^, ^63^, ^64^, ^65^, ^66^, ^67^ ^]^

## Conflict of Interests

The authors declare no conflict of interest.

## Supporting information



Supporting information

## Data Availability

The data that support the findings of this study are available from the corresponding author upon reasonable request.
