# Supplementary material for: Targeting the Spliceosomal Protein USP39 Through Allosteric Ligands and PROTAC‐Induced Degradation
Source: Angew Chem Int Ed Engl. 2025 Dec 21;65(5):e16809. doi: 10.1002/anie.202516809 (PMC12851022; doi:10.1002/anie.202516809)
Supplement: Supplementary file 1 — Supporting information [file ANIE-65-e16809-s001.docx]

Supplementary Information

**Targeting the Spliceosomal Protein USP39 Through Allosteric Ligands and PROTAC-Induced Degradation**

Daniel Schäfer, Cristian Prieto-Garcia, Jianhui Wang, Marcel Heinz, Vigor Matkovic, Pavel Kielkowski, Sebastian Hasselbeck, Varun Jayeshkumar Shah, Stefan Knapp, Gerhard Hummer, Ivan Dikic, Xinlai Cheng*

D. Schäfer, J. Wang, S. Hasselbeck, V. Matkovic, Prof. Dr. I. Dikic, Prof. Dr. S. Knapp, Dr. rer. nat. habil. X. Cheng
Buchmann Institute for Molecular Life Sciences
Goethe University Frankfurt am Main
Max-von-Laue-Str. 15, D-60438 Frankfurt am Main, Germany

D. Schäfer, J. Wang, S. Hasselbeck, Prof. Dr. S. Knapp, Dr. rer. nat. habil. X. Cheng
Institute for Pharmaceutical Chemistry
Johann Wolfgang Goethe-University
Max-von-Laue-Str. 9, D-60438 Frankfurt am Main, Germany

D. Schäfer, Prof. Dr. S. Knapp, Dr. rer. nat. habil. X. Cheng
Frankfurt Cancer Institute
Paul-Ehrlich-Str. 42-44, D-60596 Frankfurt am Main, Germany

Dr. rer. nat. habil. X. Cheng
Mildred-Scheel-Nachwuchszentrum (MSNZ), University Cancer Center (UCT) Frankfurt
University Hospital Frankfurt am Main
Theodor-Stern-Kai 7, D-60596 Frankfurt am Main, Germany

V. Matkovic, C. Prieto-Garcia, V. J. Shah, Prof. Dr. I. Dikic
Institute of Biochemistry II Frankfurt
University Hospital Building 75, Faculty of Medicine Frankfurt am Main
Theodor-Stern-Kai 7, D-60596 Frankfurt am Main, Germany

Dr. M. Heinz, Prof. Dr. G. Hummer
Department of Theoretical Biophysics
Max Planck Institute of Biophysics
Max-von-Laue-Str. 3, 60438 Frankfurt am Main, Germany

Dr. P. Kielkowski
Department of Chemistry
Ludwig Maximilian University München
Würmtalstrasse 201, 81375 Munich, Germany.

Prof. Dr. G. Hummer
Institute of Biophysics
Johann Wolfgang Goethe-University
Max-von-Laue-Str. 1, D-60438 Frankfurt am Main, Germany

** Corresponding author: Xinlai Cheng E-mail: cheng@pharmchem.uni-frankfurt.de*

**Table of Contents**

| Figure SI1 (USP39 degradation and protein purification) | Page 03 |
| --- | --- |
| Figure SI2 (SAR compounds) | Page 04 |
| Figure SI3 (USP39_PROTAC_V1 TSA and western blot) | Page 04 |
| Scheme SI1 (Synthesis of USP39_B2) | Page 04 |
| Scheme SI2 (Synthesis of USP39_B1-FITC) | Page 05 |
| Biological and chemical methods and reagents | Page 06 |
| Table SI1: Site scores of potential binding sites and shallow binding sites of the USP39 ZnF construct determined with SiteMap in Schrödinger. | Page 18 |
| Table SI2: Docking scores, XP Gscores and Glide emodel of USP39_B1 for the different binding sites. | Page 18 |
| Table SI3: Docking scores, XP Gscores and Glide emodel of synthesized ligand for shallow binding site 1 (Shallow_Site1). | Page 19 |
| Table SI4: Representation of the synthesized PROTACs. | Page 20 |
| Table SI5: Predicted results from AI-based SwissADME analysis. | Page 25 |
| Table SI6: Primer sequence | Page 26 |
| Chemical syntheses and analyses | Page 27 |
| SI-References | Page 97 |

**Figure SI1:** *Confirm USP39 degradation by proteosome and protein purification. (A) Verification of the degradability of USP39 by the ubiquitin-proteasome system using a dTAG system by the fusion of FKBP12^F36V^ with USP39. (B) Purification of USP39_FL by size exclusion chromatography and determination of the purity and mass of the protein by SDS gel and mass spectroscopy (66,442 kDa). (C) Purification of USP39_T1 by size exclusion chromatography and determination of the purity and mass of the protein by SDS gel. (D) Purification of USP39_T2 by size exclusion chromatography and determination of the purity and mass of the protein by SDS gel and mass spectroscopy (14,256 kDa) (E) Chemical structure of the DEL-screening hits* (F) Representation of non-logarithmic diagrams from fluorescence polarization assays for the binding of USP39_B1-FITC to USP39_FL (orange) and USP39_T2 (blue) with the corresponding Kd values.


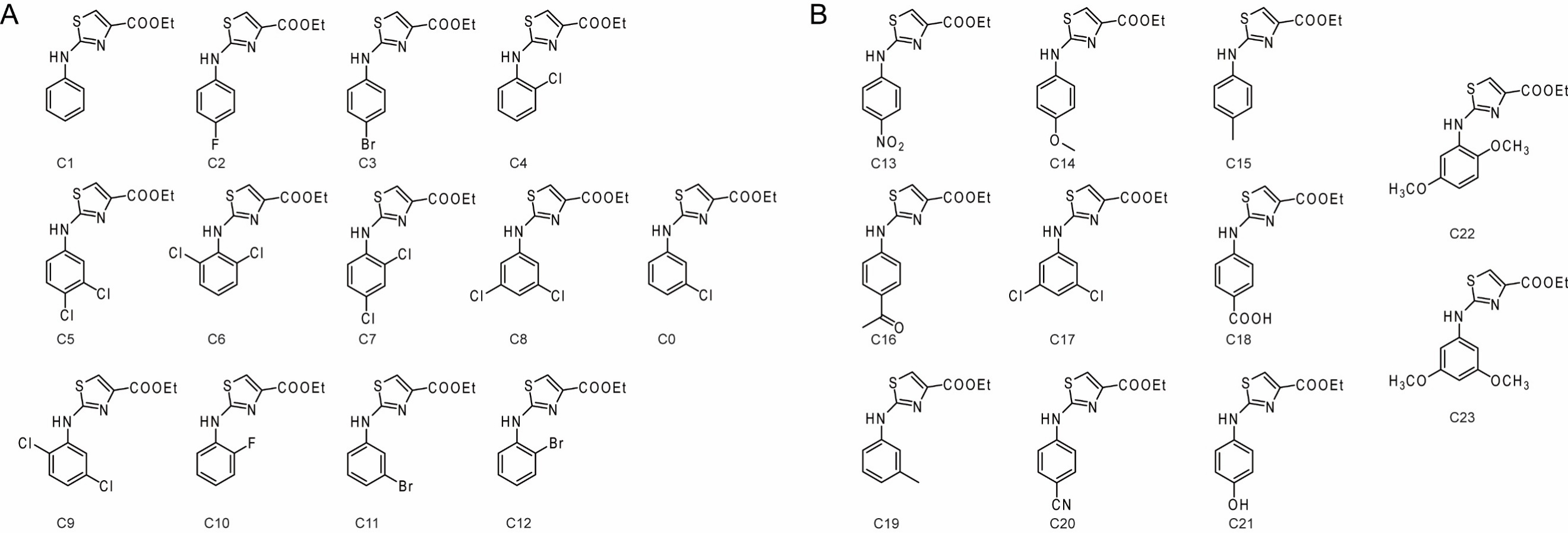


**Figure SI2:** SAR compounds. (A) Chemical structures of the compounds C0 to C12. (B) Chemical structures of the compounds C13 to C23.


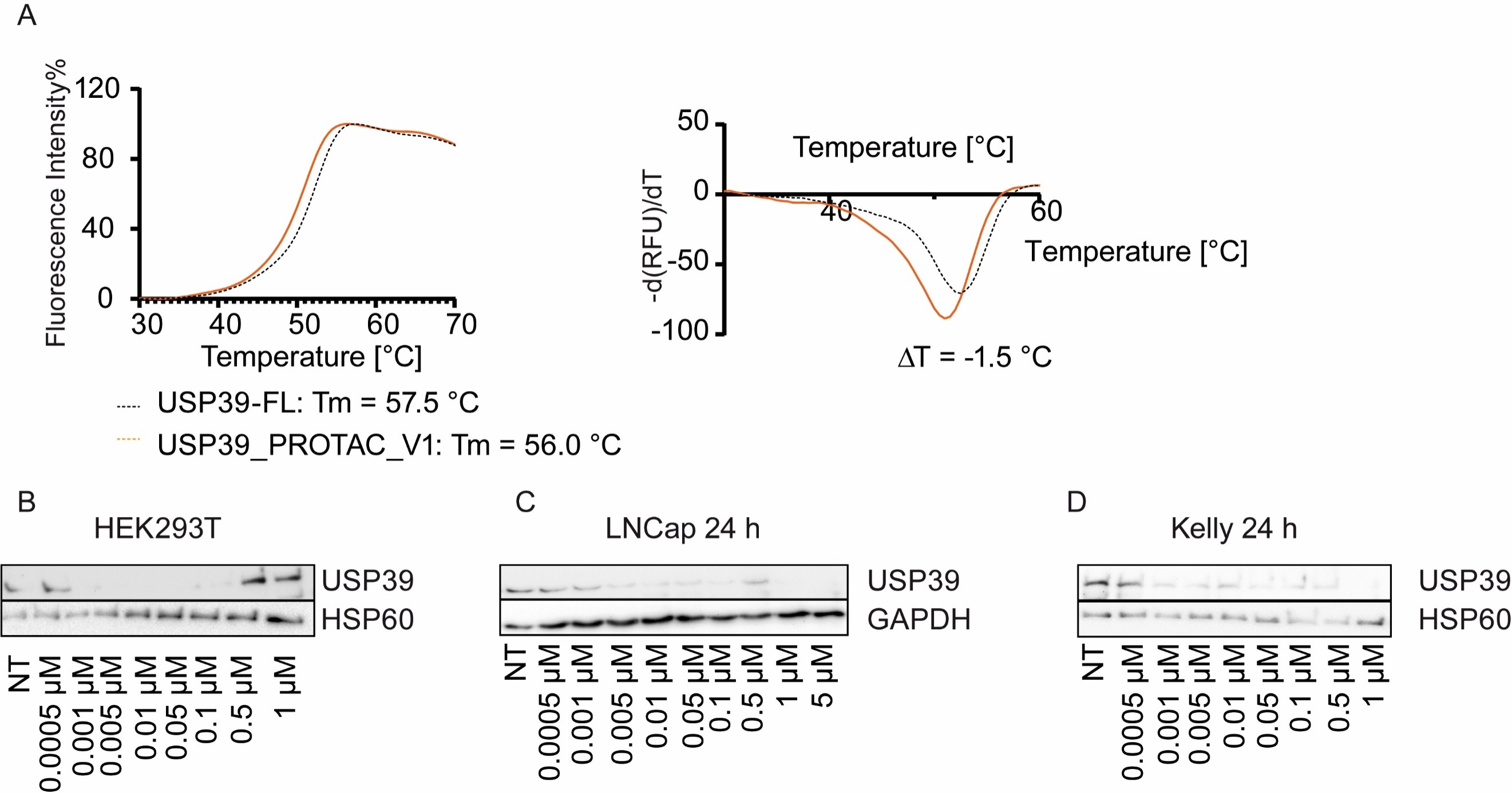


***Figure SI3****: (A) The results of the TSA measurement of USP39_PROTAC_V1 with USP39_FL. Western blot results obtained by treatment of HEK293T (B), LNCap (C) and Kelly (D) cells with a USP39_PROTAC_V1 concentration of 0.5 to 5000 nM and 24 h treatment as well as 16 h serum starvation.*


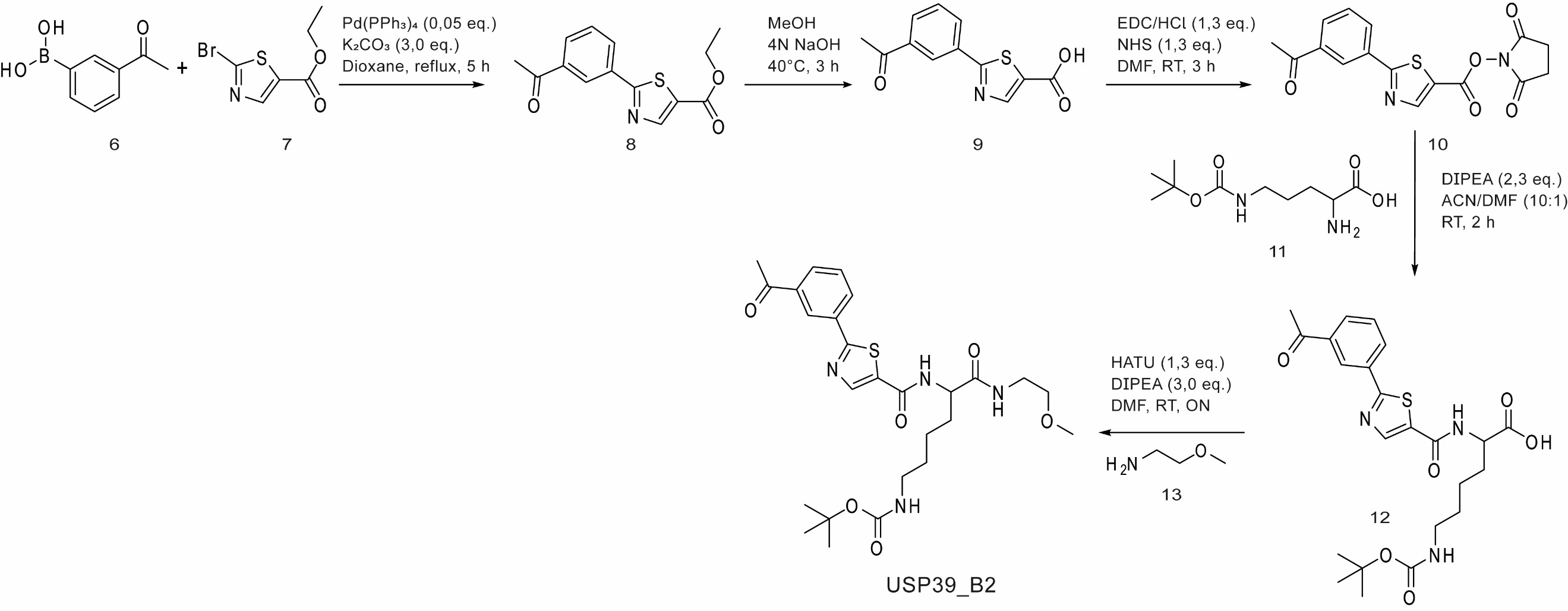


**Scheme SI1**: Synthesis of USP39_B2.


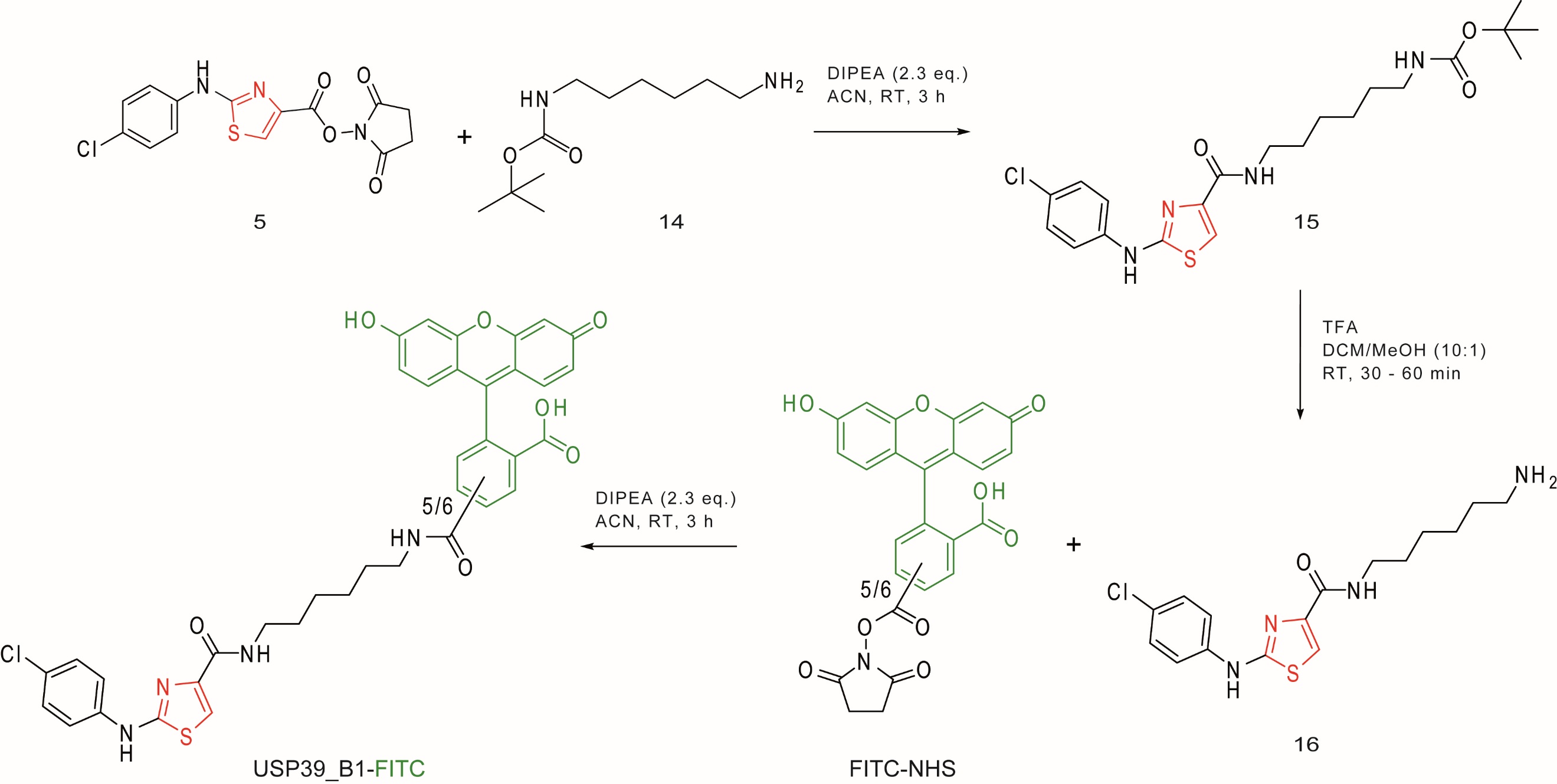


**Scheme SI2**: Synthesis of USP39_B1-FITC.

**Experiments**

**Chemistry**

**Reagents and Purity**

Solvents and reagents were obtained from commercial suppliers, meeting at least reagent-grade standards, or were purified according to established methods prior to use if necessary. Thin-layer chromatography (TLC) was performed using Alugram SIL G/UV254 sheets (Macherey & Nagel) or the puriFlash XS520Plus system from Advion Interchim to monitor chemical reactions. Column chromatographic purifications were conducted using the puriFlash XS520Plus system from Advion Interchim, employing a packed reverse-phase (RP) silica gel column and a UV detector. Elution was carried out with acetonitrile combined with a 1 vol% aqueous solution of formic acid. The purity of the compounds was confirmed through HPLC or LC/MS analysis. Deuterated solvents for NMR measurements were purchased from Eurisotop.

**Analytical methods**

**MS:**

Mass spectrometry was measured as a service at the Faculty of Biochemistry, Chemistry and Pharmacy at Goethe University, D-60438 Frankfurt am Main, Germany. The mass-analyses of the compounds were performed by ESI of sample dissolved in MeOH or DMSO. The measurement was performed on the ESI-Q-MS surveyor MSQ Master MSQ20979 (Thermo Fisher Scientific, cat: 3344722).

**NMR:**

^1^H (300 Hz/400 Hz) and ^13^C NMR (300 Hz/400 Hz) spectra were recorded on Bruker 300 MHz or 400 MHz NMR systems. Chemical shifts are reported in ppm, with DMSO-d6 used as the solvent, referenced at **δ**=2.50 for 1H and **δ**=40.0 for ^13^C. The following abbreviations were used to describe the multiplicities in the NMR spectra: s = singlet, d = doublet, t = triplet, q = quartet, quint = quintet, m = multiplet.

**High performance liquid chromatography (LS/MS)**

Determination of the compound purity by HPLC was conducted on an Agilent 1260 Infinity II device with a 1260 DAD HS detector (G7117C) and a LC/MSD device (G6125B, ESI pos. 100-1000). The compounds were analyzed on a Poroshell 120 EC-C18 (Agilent, 3 x 150 mm, 2.7 µm) reversed phase column using 0.1% formic acid in water (A) and in acetonitrile (B) as a mobile phase

**Note**: Two separate ESI-based instruments were used for compound characterization. Stand-alone ESI-MS enabled rapid confirmation of molecular ions and accurate mass determination, whereas LC-ESI-MS, performed on a dedicated LC-MS system, provided chromatographic separation coupled with mass detection to assess purity, detect by-products or degradation species, and verify retention times.

**Biology**

**Cell culture:**

HeLa (ATCC, CCL-2) and HEK293T (ATCC, CRL-3216) were cultured in Dulbecco’s modified Eagle’s medium (DMEM; Thermo Fisher Scientific, 31966021), while Kelly WT (Department of Medical Oncology, Dana-Farber Cancer Institute, Bosten, MA) and LnCap (ATCC, CRL-1740) cells were maintained in RPMI 1640 (Thermo Fisher Scientific, 61870036). All media were supplemented with 10% fetal bovine serum (FBS) and 100 mg/ml (1%) penicillin/streptomycin (PS). Serum starvation was performed with 0% fetal bovine serum (FBS) and 1% penicillin/streptomycin (PS). The cells were cultivated at 37°C in a humidified atmosphere with 5% CO_2_.

**Plasmids:**

The USP39 coding sequence was inserted into the pLEX_305-N-dTAG vector (Addgene #91797) using the Gateway recombination system (Life Technologies/Thermo Fisher Scientific), in accordance with the manufacturer’s protocol to generate the dTAG-USP39 construct.

**Transient transfection and degradation:**

HeLa cells were transiently transfected with dTAG-USP39 plasmid using standard transfection protocols. The following day, cells were treated with either DMSO (vehicle control) or dTAG13 (R&D Systems, Catalog #: 6605) at the indicated concentrations. After 24 or 48 hours, USP39 protein levels were assessed by western blotting.

**Splicing**

HEK293T (ATCC, CRL-3216) were seeded in 12-well plates, with each well containing 150 000 cells cultured in DMEM. The cells were cultivated at 37°C in a humidified atmosphere with 5% CO_2_. After 24 hours cultivation, the cell culture was starved overnight with 1.5 ml of DMEM containing 10% FBS, without PS. The following day, treat with USP39_B1 (100 nM) and USP39_PROTAC_V1 at concentrations of 0, 10 nM, 50 nM, 100 nM, and 1 µM for 24 hours. Cells were collected for the RNA extract. Reverse transcription-polymerase chain reaction (RT-PCR) was performed according to the manufacturer’s instructions (CFX96 Touch Real-Time PCR Detection System, Bio-Rad, #1855201, Germany). Cells were washed with PBS and lysed using 200 μl Qiazol reagent (Qiagen, Germany). To the lysate, 40 μl of chloroform was added, followed by vortexing for 15 s. The mixture was then centrifuged at 12 000 × g for 15 min at 4°C. The aqueous (upper) phase was transferred to a new tube, mixed with 100 μl of isopropanol and incubated for 5 min. After another centrifugation at 12 000 × g for 15 min at 4°C, the supernatant was discarded. The RNA pellet was washed with 70% ethanol, collected by centrifugation at 7000 × g for 5 min and then resuspended in 20 μl of RNase-free water. RNA quality was assessed using a NanoDrop (Thermo Fisher Scientific, Nanodrop 1000).

Equivalent amounts of RNA were reverse transcribed using the FastGene Scriptase II cDNA kit (Nippon Genetics, Germany). To each RNA sample (1 μg), 1 μl of hexamer and 1 μl of dT primer were added, followed by incubation at 42°C for 10 min and then at room temperature for 5 min. A master mixture containing Deoxynucleotide triphosphates (dNTPs), RNase inhibitor and reverse transcriptase in reaction buffer was prepared and added to each RNA sample to a final volume of 20 μl. The mixture was incubated at 42°C for 1 h and the reaction was halted by heating at 80°C for 5 min. PCR was performed using OneTaq quick-load 2X master mix with Standard Buffer (New England Biolabs, M0486L). Each 20 μl reaction contained 10 μl of SYBR Green PCR Master Mix, 2 μl of 1:10 diluted complementary DNA (cDNA), 1 μl of forward and reverse primer mix (final concentration 0.5 μM each; primer sequences are listed in Table SI5), and 7 μl of nuclease-free water. ACTB was used as the internal reference gene for normalization. Thermal cycling was carried out using the following conditions: initial denaturation at 95°C for 3 min; 34 amplification cycles of 95°C for 30 s, annealing at the gene-specific melting temperature (Tm) for 30 s, and extension at 68°C for 60 s; followed by a final extension at 72°C for 5 min. The reactions were held at 4°C until further analysis. PCR products were visualized by electrophoresis on a 2% (w/v) agarose gel stained with a nucleic acid dye and imaged under UV illumination.

**Fluorescence polarization assay (FP)**

The fluorescence polarization assay was performed in black 384-well plates (Greiner, #784076) using a microplate-reader PHERAstar® FSX to determine the interaction between the Protein (USP39FL, USP39T2, USP39MV1 and USP39MV2), the tracer and the compounds. A FP filter with the wavelengths 485-520-520 was used for the measurement. The tracer USP39_B1_FITC (0.5 nM) is used to measure fluorescence polarization. For the preparation of a stock solution, the tracer (1 nM) was added to the protein with a concentration of 10 uM (USP39FL) and 100 uM (USP39T2, USP39MV1 and USP39MV2) in the buffer (25 mM HEPES, 150 mM NaCl, 5% glycerol, 0.5 mM TCEP, pH 7.5). The protein tracer solution is added to the wells and subsequently the compound (required concentration) is added to achieve a volume of 10 uL per well with a final concentration of the tracer (0.5 nM) and the protein 5 uM (USP39FL), 50 uM (USP39T2, USP39MV1 and USP39MV2). The microtiter plate is centrifuged at 2000 RPM for one minute before starting the measurement.

**Bradford assay:**

A dilution series of BSA was prepared and applied to a 96-well plate. The concentrations used were 2 mg/mL, 1 mg/mL, 0.5 mg/mL, 0.25 mg/mL, 0.125 mg/mL, 0.075 mg/mL, and 0.0375 mg/mL. For each sample, 2 µL was added to the wells. Then, 98 µL of Bradford reagent was added to each well. The absorbance was measured at 595 nm using Tecan Infinite® 200 PRO microplate reader. Using the calibration curve, the concentration of each sample was determined and normalized to the lowest concentration.

**Western blot:**

HEK293T (ATCC, CRL-3216), HeLa (ATCC, CCL-2), Kelly WT (Department of Medical Oncology, Dana-Farber Cancer Institute, Bosten, MA) and LnCap (ATCC, CRL-1740) were seeded in 12-well plates, with each well containing 250 000 cells cultured in cell culture medium (DMEM or RPMI). The cells were cultivated at 37°C in a humidified atmosphere with 5% CO_2_. If applicable the cell culture was starved overnight after 24 hours with 1.0 ml of cell culture medium (DMEM or RPMI) containing 0% FBS and 1% PS. The following day, the 0% FBS medium was removed and 1 ml cell culture medium (DMEM or RPMI) with 10% FBS and 1% PS was added. The cells were treated with USP39_PROTAC-V1 (0.5 nM, 1 nM, 5 nM, 10 nM, 50 nM, 100 nM, 500 nM, 1000 nM, 5000 nM) and, if applicable, with MG132 (0.25 uM) for 24 hours. The very next day, the medium was removed and 80 uL lysis buffer (1 mM EDTA, 0.5% Triton X-100, 5 mM NaF, 6 M urea, 1 mM Na_3_VO_4_, 10 mg/ml pepstatin, 100 mM PMSF and 3 mg/ml aprotinin in phosphate buffered saline (PBS)) was added. The cells were shaken in the lysis buffer for 5 minutes at room temperature. The samples were subsequently centrifuged at 12 000 × g for 5 min at 4°C and the DNA pellet was removed. The protein concentration was determined and standardized using the Bradford assay (Sigma-Aldrich, Germany). To do this, the samples were diluted with the buffers supplied.

Blot detection was carried out using enhanced chemiluminescence (ECL). A total of 40 mg of total protein was separated on either 8%, 10% or 12% SDS-PAGE gels, followed by subsequent immunoblotting with specific antibodies. GAPDH, or VINCULIN antibody served as the loading control. Primary antibodies (diluted 1:1000) were incubated overnight at 4°C in TBS (pH 7.5) with 0.1% Tween-20 and 5% BSA/milk. Secondary antibodies (diluted 1:10,000, Dianova, Germany) were incubated in TBS (pH 7.5) with 5% milk and 0.1% Tween-20 for 1 hr at room temperature.

**ITC:**
All reactant solutions were kept in the protein storage buffer (25 mM HEPES, pH 7.5, 150 mM NaCl, 0.5 mM TCEP, 5% glycerol). Buffer, protein, and reactant solutions were centrifuged at full speed for 15 min at 4°C, followed by degassing with a degassing station (TA Instruments, #6326) for an additional 15 min to remove any air bubbles. The measurements were performed with a Nano ITC microcalorimeter (TA Instruments). Purified USP39_T2 was used for ITC measurements. The compound USP39_PROTAC_V1 (3 % v/v DMSO) were titrated against the protein. The compounds were also titrated against the above buffer to subtract the background noise. 250 µL of compound (150 µM, 3 % DMSO) was titrated against 1 mL of USP39_FL protein (4.29 µM, 10 % DMSO). The protein solution was transferred into the cell of the calorimeter and the compound into the syringe. Before starting the measurement, the needle was auto-calibrated for 3000 s at a stirring rate of 300 rpm, so the enthalpy reached the baseline successfully. The titration experiment took place with 24 injections a 10 µL of compound. The first default injection started with 4 µL. The data was analysed using the Nanoanalyze Software with the fitting program at multiple sites. Each set was repeated with compound-to-buffer titration. The experiments were conducted with a DMSO percentage of 10%

**In cellular fluorescence assay:**

HeLa (ATCC, CCL-2) were seeded in 96-well plates, with each well containing 9 000 cells cultured in DMEM. The cells were cultivated overnight at 37°C in a humidified atmosphere with 5% CO._._ Lipofectamine 3000 (Thermo Fisher Scientific, L3000015) was used as the transfection reagent. For the preparation of the transfection mixture, in tube I Lipofectamine (0.2 μl) was added to 6 μl Opti-MEM medium. In tube II, P3000 (0.2 μl) and 250 ng of plasmid were added to another 6 μl Opti-MEM medium. The contents of tube II were then combined with those of tube I and incubated at room temperature for 5–10 min. The cell culture medium was refreshed with 80 µl of DMEM containing 10% FCS, without PS, and then the transfection mixture was added to the cells. The cells were incubated overnight, followed by a medium replacement with DMEM supplemented with 10% FCS and 1% PS, and further incubated for 48 h before treatment. The cells were treated with USP39_PROTAC-V1 (0.5 nM, 1 nM, 5 nM, 10 nM, 50 nM, 100 nM, 500 nM, 1000 nM, 5000 nM) diluted in DMSO and, if applicable, with MLN (1 uM) for 24 hours. The medium was subsequently removed and 80 uL of a 4% paraformaldehyde-PBS solution (PFA) was added and incubated for 12 minutes. The PFA-PBS solution was removed and 60 uL of an 1 % Hoechst solution (PureBlu™ Hoechst 33342 nuclear staining dye, #1351304) in PBS was added and incubated for a further 8 minutes. The Hoechst-PBS solution is discarded and the cells are carefully washed twice with 60 uL PBS each time. After the second wash, the PBS is removed and 80 uL of fresh PBS is added. The fluorescence of the OFP-USP39 and Hoechst dye is measured using an ECHO revolve microscope RVL2-K2. The evaluation of the fluorescence present and the associated degradation of USP39 was carried out using ImageJ.

**Thermal Shift Assay**

The thermal shift assay was performed in 96-well plates (Bio-Rad, #9601) using a CFX Connect Real-Time PCR Detection System (Bio-Rad, #1855201) to determine the thermal stability of the proteins and protein-compound-complexes. According to the manufacturer`s instructions, SYPRO Orange (10×, Thermo Fisher Scientific, #S6650) was used as a fluorescent dye to measure the fluorescent intensity at an excitation/emission (Ex/Em) of 470/570 nm. In this assay, a HEPES-based buffer (25 mM, pH = 7.5) with a salt concentration of 150 mM NaCl was used and the final volume per well was 10 uL. The proteins have a final concentration of 5 uM (USP39FL), 10 uM (USP39T1) and 50 uM (USP39T2). The microtiter plate is centrifuged at 3500 RPM for one minute before starting the measurement.

**Cell transformation**

50 μL of BL21 competent E. coli cell suspension were thawed and homogenized. Then, 2 μL of plasmid DNA were added to the cell suspension. The mixture was gently inverted a few times and kept on ice for 30 minutes. Next, the mixture was briefly placed in 42°C warm water for 10 seconds, then immediately transferred to ice for 5 minutes. Afterwards, 950 μL of LB medium were added to the mixture, which was then incubated at 37°C with shaking at 450 rpm in a heating block. An agar plate containing the appropriate antibiotic was prewarmed to 37°C, inoculated with 100 μL of the mixture, and incubated overnight at 37°C. The agar plate with the culture was stored at 4°C.

**Preculture**

150 mL of LB media and 150 μL of 100 mg/mL ampicillin stock solution for the expression of USP39Fl, or 150 μL of 50 mg/mL kanamycin for the expression of USP39T2, were added to a 250 mL baffled flask. The flask was inoculated with a stab of transformed BL21 E. coli cells. The preculture was incubated at 37°C with shaking at 150 rpm overnight. From this initial preculture, a glycerol stock of the transformed cells was prepared by diluting 1 mL of the preculture with 100 μL of 50% glycerol.

**Expression culture**

In a plastic baffled flask, 1 L of LB media was added and autoclaved. 1 mL of 100 mg/mL ampicillin stock was added. The expression culture was inoculated with 10 mL of preculture (OD value ~ 0.05), incubated at 37°C and shaken at 150 rpm for approximately 2.5 hours until an OD600 of 0.6 – 1.0 was reached. Subsequently the expression culture was cooled to 18°C and the expression was induced with 1 mL of 1 M IPTG stock. Two drops of antifoam 204 were added. After the induction, the expression culture was incubated at 18°C and shaken overnight at 150 rpm.

**Cell harvest**

The expression culture was transferred into centrifuge flasks and centrifuged for 20 minutes at 4°C and 4000 rpm. If the further purification was not carried out on the same day, the cell pellet was transferred into centrifuge tubes, and stored at -80°C.

**Cell lysis**

To the lysis buffer containing 50 mM Tris, 400 mM NaCl, 20 mM imidazole, pH 7.5, the following components were added: 100 μL per 100 mL of lysozyme, 25 μL per 100 mL of DNAse, 200 μL per 100 mL of TCEP, and one tablet of protease inhibitor cocktail (Roche). The cell pellets were resuspended in 15 mL/cell pellet of lysis buffer. The cell suspension was stirred gently for 20 minutes to ensure homogenization. Next, the cells were sonicated at 35% power with 5-second pulses and 10-second pauses at 4°C, continuing until a noticeable change in color indicated sufficient lysis. The resulting suspension was transferred into centrifuge tubes and centrifuged at 4°C at 12,000 rpm for 1 hour.

**Purification**

NiNTA beads were incubated with the supernatant for 30 minutes under gentle shaking, whereas the pellet was discarded. Subsequently, the NiNTA beads were transferred into a chromatography column. The NiNTA beads with the adsorbed POI were washed twice with 50 mL lysis buffer. The elution was performed first with elution buffer A (50 mM Tris, 400 mM NaCl, 40 mM imidazole, pH 7.5) and eluction buffer B (50 mM Tris, 400 mM NaCl, 400 mM imidazole, pH 7.5).

**SDS-PAGE**

For SDS-PAGE analysis, a 10% acrylamide gel was used for the full-length USP39, and a 15% acrylamide gel was employed for analyzing the zinc-finger domain. To prepare the samples, 10 µL of loading dye (BlueStar Plus prestained protein marker, Nippon genetics, # MWP04) was added to 40 µL of each sample, which were then incubated at 95°C for 5 minutes. Subsequently, 10 µL of the prepared samples were loaded onto the gel. The gasket was filled with SDS running buffer, the voltage was set to 230 V, and the samples were electrophoresed for approximately 50 minutes. After electrophoresis, the gel was stained with Coomassie solution for 15 minutes. To remove excess stain and destain the background, the gel was shaken in Coomassie destaining solution for 45 minutes.

**Size exclusion chromatography (SEC)**

Fractions of were combined according to the SDS-PAGE and concentrated using Amicon Ultra centrifuge filter with a 30 kDa (USP39Fl, USP39T1), respectively 10 kDa (USP39T2) molecular weight cut off in the centrifuge at 4°C and 4000 rpm. The concentrated protein solution was purified by SEC. The fractions containing the protein were determined with SDS-PAGE, combined, and concentrated using the Amicon Ultra centrifuge filter at 4°C and 4000 rpm. The protein was aliquoted and stored at -80°C using cryopreservation with liquid nitrogen.

**Alpha-Assay**

The USP39_Fl was purified as described above. The VHL-GST protein was purchased from Sigma Aldrich. Both were diluted to a concentration of 100 nM in an alphaLISA-PPI buffer (#AL015C, PerkinElmer) containing 250 mM HEPES (pH = 7.3), 500 mM NaCl, 2.5% Triton X-100 and 2.5% BSA. A mixture of 10 nM USP39Fl and 10 nM VHL-GST in 9 µL of the PPI buffer was exposed to increasing concentrations of either USP39_PROTAC_V1 or USP39_B1 (1 µL of each compound) and incubated at room temperature for 60 minutes. Subsequently, 1 µL of GST acceptor beads (at a concentration of 500 µg/mL, #AL110C, PerkinElmer) and 1 µL of FLAG donor beads (at a concentration of 500 µg/mL, #AS103D, PerkinElmer) were added to the reaction buffer and incubated for an additional 60 minutes at room temperature. The plate reader used for this experiment was the Alphaplate-384 (#6005350, Perkim) with an excitation/emission wavelength of 680 nm/615 nm.

**Immunoprecipitation**

HeLa cells were initially seeded in a 6-well plate at a density of 300,000 cells per well and incubated overnight. The cells were treated with USP39_PROTAC_V1 or USP39_B1 (100 nM) in the presence or absence of MLN4924 (10 µM) for 6 h. The cells were then lysed in an immunoprecipitation lysis buffer (comprising 20 mM Tris HCl at pH 8, 150 mM NaCl, 1% Nonidet P-40, and 2 mM EDTA). A cocktail of protease and protein-phosphatase inhibitors was freshly added, including 5 mM NaF, 1 mM Na3VO4, 10 mg/mL Pepstatin, 100 mM PMSF, and 3 mg/mL Aprotinin.

A pre-cleaning step was performed by adding rabbit serum 20 µL for 1 h at 4°C and then incubated with 20 µL of protein A magnetic beads for 30 min at 4°C. The supernatant was incubated with 2 µL USP39 antibody with gentle rocking overnight at 4°C. Protein A magnetic beads were added and incubated for 1-3 h at 4°C. Pellet was washed several times with lysis buffer and re-suspended in the loading buffer and heated to 95°C for 3 min.

**Fluorescence resonance energy transfer (FRET) assay**

The FRET assay was performed as previously reported.^2^ HEK293T cells were seeded in a black 96-well plate at a density of 15 000 cells/well. The cells were transfected with USP39-OFP and VHL-EGFP, as described above. After 48 h, the cells were treated with increasing concentrations of BRD4-ProGrader1 for 4 h combined with MG132 (0.25 µM) to block the degradation. FRET signals were recorded by a plate reader (Ex/Em: 488 nm/573 nm).

**DEL-Screening**

His-tagged USP39 constructs (USP39-full length, USP39-ZnF (zinc finger domain, USP39 98-196aa) domain and USP39-DUB domain (219-555aa)) were transformed into BL21(DE3) competent cells and single-colony inoculations were induced at an OD600 of 0.6 with 0.5 mM IPTG for 18 h at 18 °C. Cultures were centrifuged and cell pellets stored at −70 °C. All cells were lysed by sonication and centrifuged at 35,000×g. The clarified cell extract was incubated with 2.5 mL of Ni- NTA resin pre-equilibrated with lysis buffer (50 mM HEPES pH 7.5, 500 mM NaCl, 10 mM imidazole, 5% Glycerol, 0.5 mM TCEP). The column was washed with 100 mL Binding Buffer (50 mM HEPES pH 7.5, 500 mM NaCl, 5% glycerol, 10 mM imidazole, 0.5 mM TCEP), 50 mL wash buffer (50 mM HEPES pH 7.5, 500 mM NaCl, 5% glycerol, 40 mM imidazole, 0.5 mM TCEP) and eluted with 15 mL of Elution Buffer (50 mM HEPES pH 7.5, 500 mM NaCl, 5% glycerol, 250 mM imidazole, 0.5 mM TCEP). The eluant fractions were concentrated to 5 mL and applied to a Superdex 200 16/60 column pre-equilibrated in GF Buffer (50 mM HEPES pH 7.5, 200 mM NaCl, 0.5 mM TCEP, 5% glycerol). Eluted protein fractions were pooled and concentrated to 5 mg mL−1. Following purification, all His-tagged USP39 constructs (full-length, ZnF domain, and DUB domain) were used for compound screening using the GenDECL (DNA-Encoded Chemical Library) platform from GenScript. For pulldown experiments, Dynabeads™ His-Tag Isolation and Pulldown magnetic beads were employed to immobilize the His-tagged proteins according to the instructions provided in the GenDECL Kit Guide (GenScript, L00862).

Pulldown, wash, and elution steps were performed as outlined in the GenDECL protocol. Briefly, 10 μg of target protein was incubated with the magnetic beads, followed by incubation with the GenDECL compound library. After a series of washing steps to remove unbound compounds, bound chemical entities were eluted. This selection was carried out in three successive rounds to enrich for high-affinity binders.

The final eluates from the second and third selection rounds were sent to GenScript for next-generation sequencing (NGS) library preparation, sequencing, and barcode-based data analysis as part of their add-on screening service. All steps from amplification to hit identification were carried out by GenScript's technical team as described in the official GenDECL user manual (L00862, November 2024 edition).

**Computer guided compound-protein interaction study:**

Initial structure of the USP39 (domain: 98-195aa: ZnF domain) construct with His-tag was generated using AlphaFold 3 (AF3) webserver^3^ for the sequence: MHHHHHHSSGVDLGTENLYFQSMEDRRSRHCPYLDTINRSVLDFDFEKLCSISLSHINAYACLVCGKYFQGRGLKSHAYIHSVQFSHHVFLNLHTLKFYCLPDNYEIIDSSLEDITYVLKPT.

The structure for ligand USP39_B1 was generated in Schrödinger version 2024-2 using the 2D Sketcher and processed using Ligprep (Schrödinger Release 2024-2: LigPrep, Schrödinger, LLC, New York, NY, 2024).

The AF3 generated protein structure was processed using the standard protein preparation workflow.^4^ Potential ligand binding sites were determined by SiteMap following the standard grid protocol for receptor binding sites, and also for shallow binding sites (Table SI1-3).^5^

Shallow_Site5 was not further considered as druggable due to its low SiteScore. Receptor grids for the remaining binding sites were generated and ligand was docked with extra precision (XP) using Glide.^6, 7^

We proceeded with Shallow_Site1 and docked all other synthesized ligands. The experimental observed binder has the best docking score.

**Mass spectrometry-based proteomics:**

All samples were prepared in 96-well plate format using the optimized SP3 protocol and UHPLC MS grade solvents.^8^ The cells pellets were lysed in 1% NP40, 0.2% (w/v) SDS in 25 mM HEPES, pH 7.5. Protein concentration in lysates was determined using bicinchoninic acid assay (BCA) from Pierce (#23221). Protein amount was adjusted to 10 μg in total volume of 10 μL lysis buffer. The protein was loaded onto a mixture of 1:1 hydrophilic (Sera-Mag SpeedBeads Carboxylate, GE Healthcare, 45152105050350) and hydrophobic carboxylate-coated magnetic beads (Sera-Mag SpeedBeads Carboxylate, GE Healthcare, 65152105050350, 1 μL each at 10 μg/μl) pre-washed three times with 100 µL of H2O. The magnetic beads with protein sample were mixed at 850 rpm, 1 min at room temperature (RT). To initiate the binding, 20 µL of acetonitrile containing 0.25% formic acid was added, and the mixture was incubated at RT for 8 min at 850 rpm. Subsequently, the beads were washed three times with 180 μl of 80% (v/v) EtOH and once with 180 μl acetonitrile, with incubation at RT for 30 s and 850 rpm between each wash. After the last wash the beads were resuspended in 21 μL of 100 mM ammonium acetate buffer (ABC). The wash steps and ABC buffer addition was performed by liquid handling robot (Hamilton Microlab Prep). The on-beads digestion was performed with trypsin (Promega, V5113, 1 μg) overnight 37 ˚C and 850 rpm. The resulting peptide mixture was eluted from the magnetic beads into a new 1.5 mL tube. The magnetic beads were washed with 50 and 30 μL of 1% (v/v) formic acid and incubated at 40 ˚C, 850 rpm for 5 min. The fractions were added to the first elution fraction. The combined fractions were further purified from remaining magnetic beads on magnet. MS measurements were performed on an Orbitrap Eclipse Tribrid Mass Spectrometer (Thermo Fisher Scientific) coupled to an UltiMate 3000 Nano-HPLC (Thermo Fisher Scientific) via a nanospray Flex ion source (Thermo Fisher Scientific) equipped with column oven (Sonation) and FAIMS interface (Thermo Fisher Scientific). Peptides were loaded on an Acclaim PepMap 100 μ-precolumn cartridge (5 μm, 100 Å, 300 μm ID x 5 mm, Thermo Fisher Scientific) and separated at 40°C on a PicoTip emitter (noncoated, 15 cm, 75 μm ID, 8 μm tip, New Objective) that was in-house packed with Reprosil-Pur 120 C18-AQ material (1.9 μm, 150 Å, Dr. A. Maisch GmbH). Buffer composition. Buffer A consists of MS-grade H2O supplemented with 0.1% FA. Buffer B consists of acetonitrile supplemented with 0.1% FA. The LC gradient from 4 to 35.2 % buffer B in 36 min was used. The flow rate was 0.3 µL/min.

**Data-independent acquisition:**

The DIA duty cycle consisted of one MS1 scan followed by 30 MS2 scans with an isolation window of the 4 m/z range, overlapping with an adjacent window at the 2 m/z range. MS1 scan was conducted with Orbitrap at 60000 resolution power and a scan range of 200 – 1800 m/z with an adjusted RF lens at 30%. MS2 scans were conducted with Orbitrap at 30000 resolution power, RF lens was set to 30%. The precursor mass window was restricted to a 500 – 740 m/z range. HCD fragmentation was enabled as an activation method with a fixed collision energy of 35%. FAIMS was performed with one CV at -45V for both MS1 and MS2 scans during the duty cycle.

Standalone DIA-NN software under version 2.0 was used for protein identification and quantification.^9, 10^ First, a spectral library was predicted in silico by the software’s deep learning-based spectra, RTs and IMs prediction using Uniprot Human FASTA (containing canonical and isoforms downloaded on 02.05.2025). DIA-NN search settings: FASTA digest for library-free search/library generation option was enabled, together with a match between runs (MBR) option and precursor FDR level set at 1%. The mass accuracy and the scan window were set to 0 to allow the software to identify optimal conditions. The precursor m/z range was changed to 500-740 m/z to fit the measuring parameters.

**Proteome analysis**:

Perseus (1.6.10.43) was used to log2 transform LFQ intensities, replace missing values from normal distribution and construct the volcano plots.^11^

The mass spectrometry proteomics data have been deposited at the ProteomeXchange Consortium (http://proteomecentral. proteomexchange.org) via the PRIDE partner repository, with data set identifier PXD000000 [PXD number = PXD065738].

**Supplementary Tables**

**Table SI1:** Site scores of potential binding sites and shallow binding sites of the USP39 ZnF construct determined with SiteMap in Schrödinger.

| Binding site | SiteScore |
| --- | --- |
| Site2 | 0.590 |
| Site1 | 0.575 |
| Shallow_Site1 | 0.903 |
| Shallow_Site4 | 0.902 |
| Shallow_Site2 | 0.872 |
| Shallow_Site3 | 0.864 |
| Shallow_Site5 | 0.462 |

**Table SI2:** Docking scores, XP Gscores and Glide emodel of USP39_B1 for the different binding sites.

| Binding site | Docking score | XP GScore | Glide emodel |
| --- | --- | --- | --- |
| Site2 | -1.710 | -1.710 | -25.177 |
| Site1 | -4.015 | -4.015 | -34.769 |
| Shallow_Site1 | -4.940 | -4.940 | -37.898 |
| Shallow_Site4 | -1.442 | -1.442 | -31.847 |
| Shallow_Site2 | -0.858 | -0.858 | -26.010 |
| Shallow_Site3 | -2.080 | -2.080 | -28.508 |

**Table SI3:** Docking scores, XP Gscores and Glide emodel of synthesized ligand for shallow binding site 1 (Shallow_Site1).

|  | Docking score | XP Gscore | Glide emodel |
| --- | --- | --- | --- |
| USP39_B1 | -4.940 | -4.940 | -37.898 |
|  | -4.582 | -4.582 | -33.903 |
| C1 | -4.565 | -4.565 | -35.553 |
|  | -4.103 | -4.103 | -40.952 |
|  | -4.064 | -4.064 | -39.046 |
|  | -4.027 | -4.027 | -33.366 |
|  | -2.898 | -2.898 | -40.554 |
| C0 | -2.863 | -2.863 | -40.308 |
| C3 | -2.751 | -2.751 | -41.113 |
| C8 | -2.697 | -2.697 | -43.281 |
|  | -2.519 | -2.519 | -38.832 |

**Table SI4:** Representation of the synthesized PROTACs.

| **Name** | **X** | **Y** | **R** | **Z** |
| --- | --- | --- | --- | --- |
| **Structure** | | | | |
| **PROTAC_V1** | Cl | H | PEG(3) | VHL |
|  | | | | |
| **PROTAC_V2** | H | Cl | PEG(3) | VHL |
|  | | | | |
| **PROTAC_V3** | H | F | PEG(3) | VHL |
|  | | | | |
| **PROTAC_V5** | Cl | H | -C2-piperazine-C4 | VHL |
|  | | | | |
| **PROTAC_V6** | H | Cl | C4 | VHL |
|  | | | | |
| **PROTAC_V7** | H | F | C4 | VHL |
|  | | | | |
| **PROTAC_V9** | Cl | H | none | VHL |
|  | | | | |
| **PROTAC_V10** | Cl | H | C5 | VHL |
|  | | | | |
| **PROTAC_V11** | Cl | H | PEG(1) | VHL |
|  | | | | |
| **PROTAC_C1** | H | Cl | PEG(3) | CRBN |
|  | | | | |
| **PROTAC_C2** | H | F | C4 | CRBN |
|  | | | | |
| **PRTOAC_C3** | Cl | H | C6 | CRBN |
|  | | | | |
| **PROTAC_C4** | Cl | H | C2-piperazine | CRBN |
|  | | | | |
| **PROTAC_C5** | Cl | H | PEG(1) | CRBN |
|  | | | | |
| **PROTAC_C6** | H | Cl | C4 | CRBN |
|  | | | | |
| **PROTAC_C7** | Cl | H | PEG(3) | CRBN |
|  | | | | |
| **PROTAC_C8** | H | F | PEG(3) | CRBN |
|  | | | | |
| **PROTAC_C9** | Cl | H | C2 | CRBN |
|  | | | | |

**Table SI5:** Predicted results from AI-based SwissADME analysis.^A^ (P = USP39_PROTAC)

| Compound | MW [g/mol] | HBA | HBD | Con_LogP | RotB | TPSA [A^2^] | ESOL_LogS | GI abs. | BBB | CYP1A2 | CYP2C19 | CYP2C9 | CYP3A4 | CYP3A4 |
| --- | --- | --- | --- | --- | --- | --- | --- | --- | --- | --- | --- | --- | --- | --- |
| DT2216^B^ | 1445.22 | 15 | 5 | 8.89 | 34 | 280.24 | -14.18 | Low | No | No | No | No | No | Yes |
| KT333^B^ | 1257.78 | 15 | 9 | 3.05 | 29 | 391.21 | -8.02 | Low | No | No | No | No | No | No |
| PRT3789^B^ | 891.09 | 12 | 4 | 3.82 | 15 | 213.79 | -7.87 | Low | No | No | Yes | No | No | Yes |
| ARV110^C^ | 812.29 | 11 | 2 | 3.50 | 10 | 181.17 | -6.93 | Low | No | No | No | Yes | No | Yes |
| ARV471^C^ | 723.90 | 5 | 2 | 5.00 | 7 | 96.43 | -8.20 | High | No | No | Yes | No | No | Yes |
| ARV825^C^ | 923.43 | 12 | 3 | 4.81 | 20 | 232.91 | -7.59 | Low | No | No | No | Yes | No | Yes |
| P_V1 | 870.48 | 10 | 5 | 4.50 | 26 | 249.82 | -6.72 | Low | No | No | No | No | No | Yes |
| P_V2 | 870.48 | 10 | 5 | 4.49 | 26 | 249.82 | -6.72 | Low | No | No | No | No | No | Yes |
| P_V3 | 854.02 | 11 | 5 | 4.27 | 26 | 249.82 | -6.28 | Low | No | No | No | No | No | Yes |
| P_V4 | 1091.34 | 14 | 6 | 5.01 | 37 | 322.29 | -7.30 | Low | No | No | No | No | No | Yes |
| P_V5 | 892.53 | 9 | 5 | 4.06 | 21 | 245.68 | -7.23 | Low | No | No | No | No | No | Yes |
| P_V6 | 752.35 | 7 | 5 | 4.53 | 18 | 222.13 | -7.06 | Low | No | No | No | No | No | Yes |
| P_V7 | 735.89 | 8 | 5 | 4.32 | 18 | 222.13 | -6.63 | Low | No | No | No | No | No | Yes |
| P_V8 | 804.93 | 1 | 5 | 1.59 | 26 | 295.64 | -4.61 | Low | No | No | No | No | No | Yes |
| P_V9 | 681.27 | 6 | 4 | 4.73 | 13 | 193.03 | -7.49 | Low | No | Yes | Yes | Yes | No | Yes |
| P_V10 | 766.37 | 7 | 5 | 4.76 | 18 | 222.13 | -7.40 | Low | No | No | No | No | No | Yes |
| P_C1 | 699.17 | 9 | 4 | 2.53 | 19 | 205.53 | -4.25 | Low | No | No | No | Yes | No | Yes |
| P_C2 | 564.59 | 7 | 4 | 2.33 | 11 | 177.84 | -4.16 | Low | No | No | No | Yes | No | Yes |
| P_C3 | 609.10 | 6 | 4 | 3.10 | 13 | 177.84 | -5.07 | Low | No | No | No | Yes | No | Yes |
| P_C4 | 622.09 | 7 | 3 | 2.23 | 9 | 172.29 | -5.22 | Low | No | No | No | Yes | No | Yes |
| P_C5 | 611.07 | 7 | 4 | 2.34 | 13 | 187.07 | -4.32 | Low | No | No | No | Yes | No | Yes |
| P_C6 | 581.04 | 6 | 4 | 2.53 | 11 | 177.84 | -4.60 | Low | No | No | No | Yes | No | Yes |
| P_C7 | 699.17 | 9 | 4 | 2.55 | 19 | 205.53 | -4.25 | Low | No | No | No | Yes | No | Yes |
| P_C8 | 682.72 | 10 | 4 | 2.28 | 19 | 205.53 | -3.81 | Low | No | No | No | Yes | No | Yes |
| P_C9 | 552.99 | 6 | 4 | 2.03 | 9 | 177.84 | -4.41 | Low | No | No | No | Yes | No | Yes |

^a^Accessed on Oct-22–25 ; ^B^VHL-based PROTACs as reference ; ^C^CRBN-based PROTACs as reference

MW: Molecular Weight; HBA: H-Bond Acceptors; HBD: H-Bond Donors; Con_LogP: consensus LogP; RotB: Rotatable Bonds; TPSA: Topological Polar Surface Area; ESOL_LogS: predicted aqueous solubility (Log10 mol/L); GI: Gastrointestinal absorption; BBB: Blood–brain barrier.

**Table SI6:** Primer sequence.

| Primer | Forward | Reverse |
| --- | --- | --- |
| ZNF414_N1 | CTGAGAGGTGGAAAATGGCG | CCCGGAGACTATGCTGGTC |
| CRK_N1 | CTGTGAAGCTGAAACCGGAG | AATCACTCCACTACCCTGCC |

*General synthesis instructions for the preparation of E3 ligases with various linkers:*

(A) 1 eq. linker (**17**), 1 eq. E3 ligand (VHL or CRBN ligand), 1.5 eq. HATU and 3 eq. DIPEA were dissolved in 3 ml/mmol DMF and stirred at RT overnight. The reaction was purified by RP FCC. The resulting product (E3-Ligase-Linker-NBOC) is converted directly to free amine in the second step. (B) 1 eq. E3-Ligase-Linker-NBOC was dissolved in 2.5 ml/mmol (DCM/MeOH, 10:1) and followed by the addition of 2.5 ml/mmol TFA. The reaction was stirred at RT for 1 h. The DCM and TFA are removed under reduced pressure and purified by RP FCC to obtain the E3-Ligase-Linker-NH_2_.

*General synthesis instructions for the preparation of the Warhead with an activated carboxylic acid:*

(A) 1 eq. USP39_B1 was dissolved in 7 ml/mmol and 7 ml/mmol 4N NaOH solution was added. The reaction solution was stirred at 40 °C for 3 h and monitored using TLC. The reaction was cooled to 0°C and the precipitate was removed. The pH was adjusted to <4 by adding 6N HCl to obtain the product (USP39_B1-COOH). The product was converted directly into the activated carboxylic acid. (B) 1 eq. USP39_B1-COOH, 1.2 eq. EDC-HCl and 1.2 eq. NHS were dissolved in 5 ml/mmol DMF and the reaction was stirred at RT for 3 h. The reaction was monitored using TLC. 50 mL/mmol of dist. water was added to the reaction solution, and it was stirred for 20 minutes at 0°C. The resulting precipitate is filtered, yielding the activated carboxylic acid (**5**).

*Synthese von 5-(4-(2-aminoethyl)piperazin-1-yl)-2-(2,6-dioxopiperidin-3-yl)isoindoline-1,3-dione:*

(A) 200 mg (0.72 mmol, 1 eq.) 2-(2,6-dioxopiperidin-3-yl)-5-fluoroisoindoline-1,3-dione (**18**) was dissolved in 15 ml/mmol N-Methyl-2-Pyrrolidon (NMP) and stirred at RT for 10 minutes. Afterwards, 216 mg (0.94 mmol, 1.3 eq.) tert-butyl (2-(piperazin-1-yl)ethyl)carbamate (**19**) and 38 mg (0.05 ml, 0.29 mmol, 0.4 eq.) DIPEA are added and the reaction is heated to 90°C and stirred overnight. The reaction was purified by RP FCC and the resulting tert-butyl-(2-(4-(2-(2,6-dioxopiperidin-3-yl)-1,3-dioxoisoindolin-5-yl)piperazin-1-yl)ethyl)carbamate (**20**) is converted directly to the free amine. (B) The product from (A) was dissolved in 2.5 ml/mmol (DCM/MeOH, 10:1) and followed by the addition of 2.5 ml/mmol TFA. The reaction was stirred at RT for 1 h. The DCM and TFA are removed under reduced pressure and purified by RP FCC to obtain 5-(4-(2-aminoethyl)piperazin-1-yl)-2-(2,6-dioxopiperidin-3-yl)isoindoline-1,3-dione.

^1^H-NMR (400 MHz, DMSO-d_6_): **δ** 11.10 (s, 1H), 8.03 (s, 3H), 7.76 (d, *J* = 8.5 Hz, 1H), 7.48 (d, *J* = 2.3 Hz, 1H), 7.36 (dd, *J* = 8.6, 2.3 Hz, 1H), 5.10 (dd, *J* = 13.0, 5.4 Hz, 1H), 3.69 (s, 3H), 3.39 (q, *J* = 7.0 Hz, 2H), 2.90 (ddd, *J* = 17.2, 14.0, 5.5 Hz, 1H), 2.61 – 2.53 (m, 1H), 2.09 – 1.98 (m, 1H), 1.10 (t, *J* = 7.0 Hz, 2H).

^13^C-NMR (400 MHz, DMSO-d_6_): **δ** 173.27, 170.50, 167.91, 167.38, 159.04, 158.70, 134.29, 125.45, 119.05, 118.34, 115.41, 109.25, 65.39, 53.47, 51.70, 51.65, 49.39, 49.35, 49.32, 40.63, 40.41, 40.20, 39.99, 39.78, 39.56, 39.35, 31.43, 22.61, 15.63.

*Synthese von tert-butyl-(6-(2-((4-chlorophenyl)amino)thiazole-4-carboxamido)hexyl)carbamate (15):*

150 mg (0.43 mmol, 1 eq.) 2,5-dioxopyrrolidin-1-yl 2-(3-acetylphenyl)thiazole-5-carboxylate (**5**), 93 mg (0.43 mmol, 1 eq.) tert-butyl-(6-aminohexyl)carbamate and 128 mg (0.17 ml, 1.0 mmol, 2.3 eq.) N,N-Diisopropylethylamine (DIPEA) were dissolved in 9 ml ACN and 1 ml DMF. The reaction was stirred for 3 h at RT and monitored using TLC. Subsequently, the reaction was then purified by RF FCC and a white solid was obtained (183 mg, 94%).

^1^H-NMR (300 MHz, DMSO): **δ** 10.44 (s, 1H), 8.09 (t, J = 6.1 Hz, 1H), 7.82 – 7.72 (m, 2H), 7.42 – 7.30 (m, 2H), 6.78 (t, J = 5.4 Hz, 1H), 3.27 (q, J = 6.8 Hz, 2H), 2.92 (q, J = 6.5 Hz, 2H), 1.55 (q, J = 9.2 Hz, 2H), 1.39 (s, 14H).

^13^C-NMR (300 MHz, DMSO): **δ** 163.19, 160.96, 156.08, 146.70, 140.15, 129.36, 129.33, 129.25, 125.22, 119.17, 113.56, 77.77, 40.60, 40.31, 40.03, 39.75, 39.46, 29.85, 28.76, 28.71, 26.63, 26.46.

*Synthese von N-(6-aminohexyl)-2-((4-chlorophenyl)amino)thiazole-4-carboxamide (16):*

100 mg (0.22 mmol, 1 eq.) Tert-butyl-(6-(2-((4-chlorophenyl)amino)thiazole-4-carboxamido)hexyl)carbamate (**15**) was dissolved in 2.5 ml/mmol (DCM/MeOH, 10:1) and followed by the addition of 2.5 ml/mmol TFA. The reaction was stirred at RT for 1 h. The DCM and TFA are removed under reduced pressure and the resulting slime was purified by RP FCC to obtain N-(6-aminohexyl)-2-((4-chlorophenyl)amino)thiazole-4-carboxamide (**16**) as a white solid (76 mg, 98%).

Note: The product is a white solid when it is not in salt form. In salt form (TFA-salt), it was a slightly orange solid.

^1^H-NMR (300 MHz, DMSO): **δ** 10.46 (s, 1H), 8.11 (t, J = 6.1 Hz, 1H), 7.80 – 7.74 (m, 2H), 7.67 (s, 2H), 7.50 (s, 1H), 7.40 – 7.31 (m, 2H), 3.29 (q, J = 6.7 Hz, 2H), 2.81 (dt, J = 14.9, 6.3 Hz, 2H), 1.54 (q, J = 7.1 Hz, 4H), 1.44 – 1.23 (m, 4H).

^13^C-NMR (300 MHz, DMSO): **δ** 163.22, 161.03, 146.65, 140.15, 129.25, 125.22, 119.18, 113.64, 40.59, 40.30, 40.02, 39.74, 39.45, 29.71, 27.45, 26.45, 26.00.

*Synthese von USP39_B1-FITC:*

76.3 mg (0.22 mmol, 1 eq.) N-(6-aminohexyl)-2-((4-chlorophenyl)amino)thiazole-4-carboxamide (**16**), 104 mg (0.22 mmol, 1 eq.) 5/6-FITC-NHS and 65 mg (0.09 ml, 0.5 mmol, 2.3 eq.) N,N-Diisopropylethylamine (DIPEA) were dissolved in 4.5 ml ACN and 0.5 ml DMF. The reaction was stirred for 3 h at RT and monitored using TLC. Subsequently, the reaction was then purified by RF FCC and a orange solid was obtained (128 mg, 79%).

^1^H-NMR (300 MHz, DMSO): **δ** 10.43 (d, J = 4.3 Hz, 1H), 10.15 (s, 2H), 8.81 (t, J = 5.6 Hz, 1H), 8.67 (t, J = 5.6 Hz, 0H), 8.47 (d, J = 1.6 Hz, 1H), 8.26 (dd, J = 8.1, 1.6 Hz, 1H), 8.21 – 8.16 (m, 0H), 8.14 – 8.03 (m, 1H), 7.80 – 7.71 (m, 2H), 7.69 (s, 0H), 7.49 (d, J = 9.3 Hz, 1H), 7.40 – 7.32 (m, 3H), 6.70 (t, J = 2.5 Hz, 2H), 6.63 – 6.53 (m, 4H), 3.24 (ddd, J = 25.7, 13.1, 6.8 Hz, 4H), 1.62 – 1.48 (m, 5H), 1.42 – 1.28 (m, 4H).

^13^C-NMR (300 MHz, DMSO): **δ** 168.69, 168.54, 164.98, 164.80, 163.19, 163.17, 160.99, 160.95, 160.06, 155.04, 153.15, 152.32, 152.29, 146.68, 146.66, 141.32, 140.13, 140.11, 136.90, 135.15, 129.84, 129.71, 129.63, 129.23, 129.16, 128.58, 126.92, 125.21, 124.66, 123.68, 119.16, 119.14, 119.06, 113.58, 113.54, 113.21, 113.13, 109.66, 109.59, 102.75, 102.72, 83.77, 83.72, 40.33, 40.16, 39.99, 39.82, 39.65, 33.81, 29.85, 29.44, 29.36, 26.66, 26.58, 25.79, 24.92.

ESI-MS: C_37_H_31_ClN_4_O_7_S 710,16 (calculated), 711,16 [M+H]^+^ (calculated), m/z = 710,98 [M+H]^+^ (found).

LC-MS: C_37_H_31_ClN_4_O_7_S 710.16 (calculated), 711,16 [M+H]^+^ (calculated), m/z = 711,1 [M+H]^+^ (found).

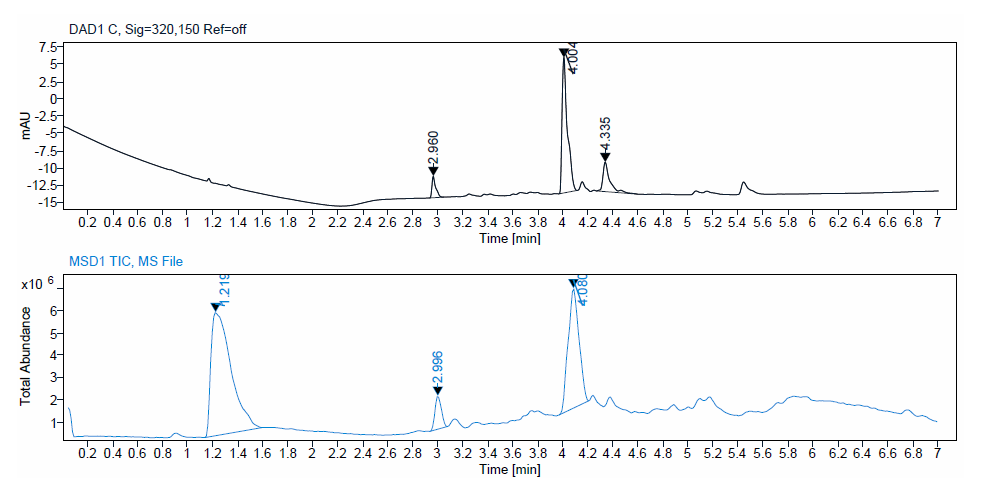


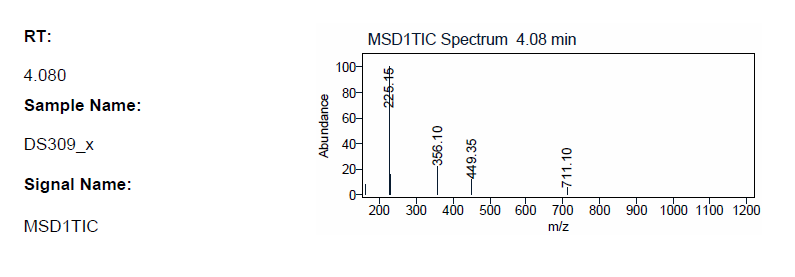


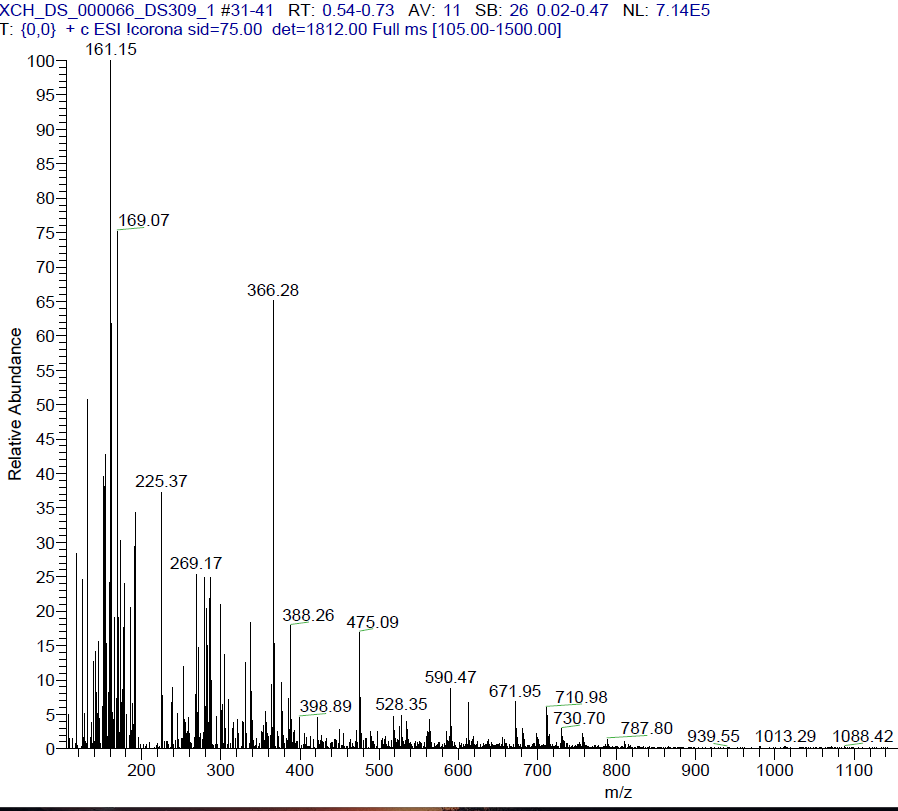


**Analyses of PROTACs from Table SI4**

*PROTAC_V1:*

^1^H-NMR (400 MHz, DMSO-d_6_): **δ** 10.46 (s, 1H), 8.98 (s, 1H), 8.57 (t, J = 6.1 Hz, 1H), 8.05 (t, J = 5.9 Hz, 1H), 7.91 (d, J = 9.3 Hz, 1H), 7.76 – 7.71 (m, 2H), 7.52 (s, 1H), 7.40 (q, J = 8.2 Hz, 4H), 7.36 – 7.31 (m, 2H), 5.13 (s, 1H), 4.55 (d, J = 9.4 Hz, 1H), 4.47 – 4.38 (m, 2H), 4.35 (s, 1H), 4.22 (dd, J = 15.8, 5.4 Hz, 1H), 3.70 – 3.44 (m, 17H), 2.45 (s, 3H), 2.38 – 2.29 (m, 1H), 2.09 – 1.99 (m, 1H), 1.90 (ddd, J = 12.9, 8.7, 4.7 Hz, 1H), 0.93 (s, 7H).

^13^C-NMR (400 MHz, DMSO-d_6_): **δ** 172.39, 170.39, 170.00, 163.25, 161.08, 151.92, 148.19, 146.33, 140.11, 139.98, 130.11, 129.28, 129.23, 129.11, 127.90, 125.27, 119.14, 113.89, 70.23, 70.18, 70.06, 69.93, 69.38, 69.34, 67.41, 40.69, 40.64, 40.48, 40.42, 40.27, 40.21, 40.00, 39.79, 26.78, 26.73, 16.41.

LC-MS: C_41_H_52_N_7_O_8_S_2_Cl 869,30 (calculated), 870,30 [M+H]^+^ (calculated), m/z = 870,20 [M+H]^+^ (found).

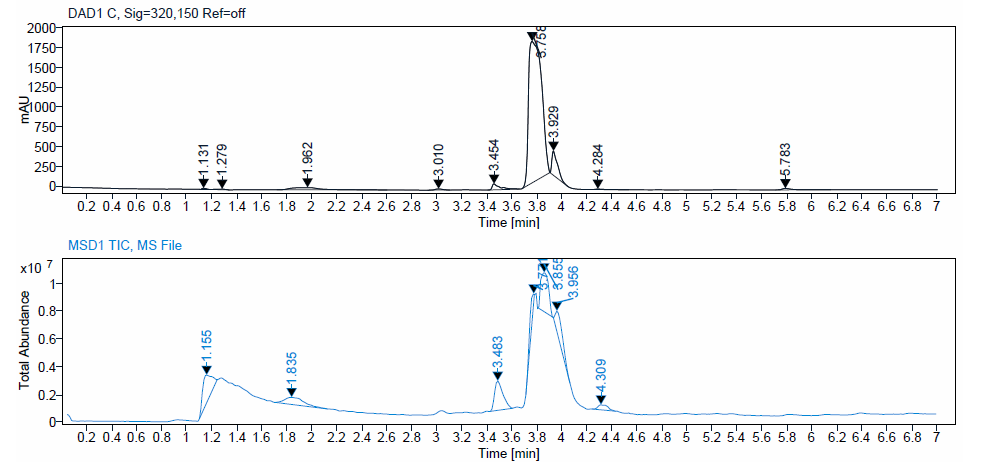


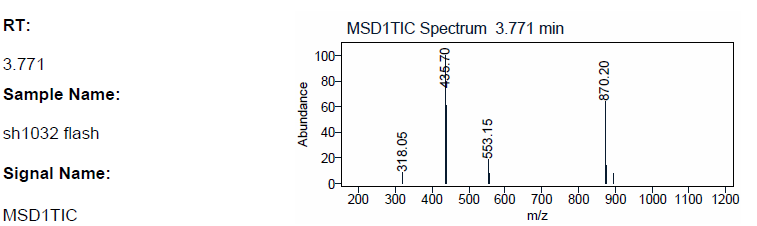


*PROTAC_V2*

^1^H-NMR (400 MHz, DMSO-d_6_): **δ** 10.50 (s, 1H), 8.99 (s, 1H), 8.56 (t, J = 6.0 Hz, 1H), 8.02 – 7.86 (m, 2H), 7.77 – 7.70 (m, 1H), 7.64 (t, J = 2.1 Hz, 1H), 7.56 (s, 1H), 7.38 (dq, J = 21.0, 8.2 Hz, 5H), 7.02 (ddd, J = 8.0, 2.1, 0.9 Hz, 1H), 4.55 (d, J = 9.4 Hz, 1H), 4.48 – 4.39 (m, 2H), 4.35 (s, 1H), 4.22 (dd, J = 15.9, 5.4 Hz, 1H), 3.70 – 3.46 (m, 17H), 2.45 (s, 3H), 2.38 – 2.27 (m, 1H), 2.03 (q, J = 9.4 Hz, 1H), 1.90 (ddd, J = 12.9, 8.6, 4.6 Hz, 1H), 0.93 (s, 9H).

^13^C-NMR (400 MHz, DMS-d_6_): **δ** 172.40, 170.40, 170.00, 164.01, 163.50, 163.14, 161.03, 151.93, 146.36, 142.53, 139.98, 133.79, 131.22, 130.11, 129.11, 127.89, 121.60, 116.75, 116.06, 114.18, 70.24, 70.18, 70.07, 69.92, 69.40, 67.40, 59.19, 56.85, 56.77, 40.42, 40.21, 39.99, 39.78, 39.57, 26.78, 16.41.

ESI-MS: C_41_H_52_N_7_O_8_S_2_Cl 869,30 (calculated), 870,30 [M+H]^+^ (calculated), m/z = 870,41 [M+H]^+^ (found).

LC-MS: C_41_H_52_N_7_O_8_S_2_Cl 869,30 (calculated), 870,30 [M+H]^+^ (calculated), m/z = 870,30 [M+H]^+^ (found).

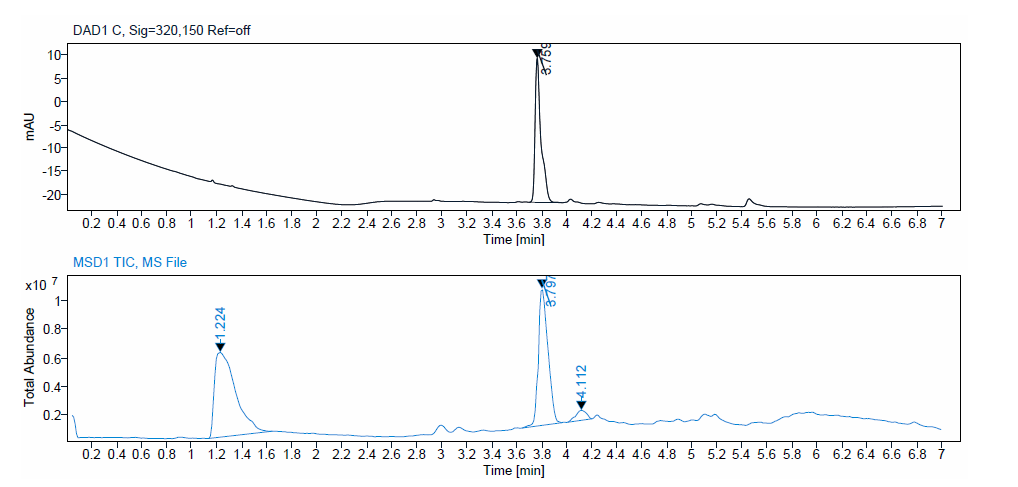


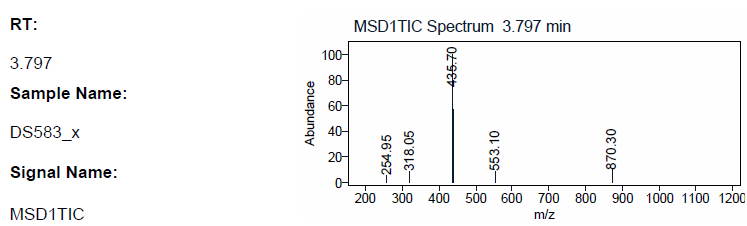


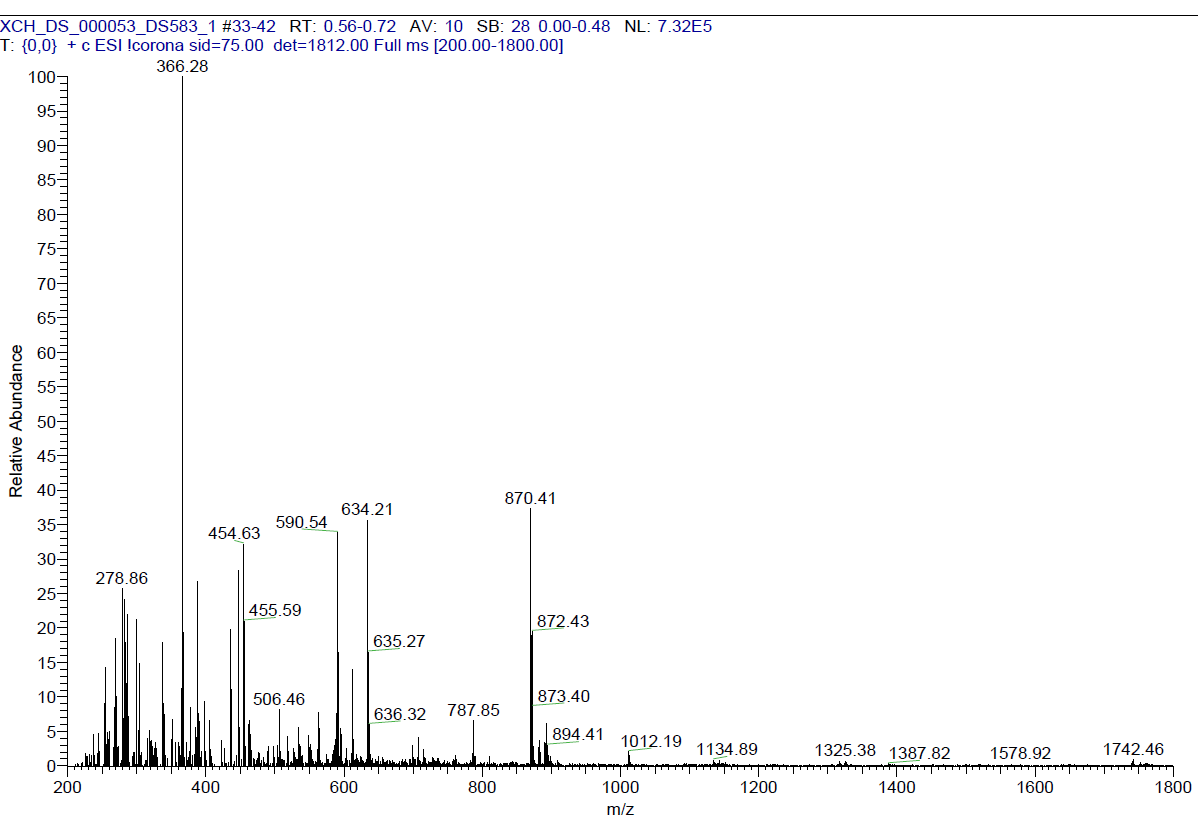


*PROTAC_V3*

^1^H-NMR (400 MHz, DMSO-d_6_): **δ** 10.53 (s, 1H), 8.98 (s, 1H), 8.56 (t, *J* = 6.1 Hz, 1H), 8.05 (t, *J* = 5.8 Hz, 1H), 7.90 (d, *J* = 9.3 Hz, 1H), 7.83 – 7.73 (m, 1H), 7.65 (dt, *J* = 11.8, 2.3 Hz, 1H), 7.55 (s, 1H), 7.45 – 7.34 (m, 7H), 6.82 – 6.73 (m, 1H), 5.14 (s, 1H), 4.54 (d, *J* = 9.4 Hz, 1H), 4.42 (dd, *J* = 9.1, 5.4 Hz, 2H), 4.35 (s, 1H), 4.22 (dd, *J* = 15.9, 5.4 Hz, 1H), 4.03 (q, *J* = 7.1 Hz, 1H), 3.71 – 3.58 (m, 4H), 3.61 – 3.40 (m, 23H), 2.44 (s, 3H), 2.33 (dt, *J* = 14.6, 6.1 Hz, 1H), 2.03 (t, *J* = 10.5 Hz, 2H), 1.90 (ddd, *J* = 12.9, 8.7, 4.6 Hz, 1H), 1.18 (t, *J* = 7.1 Hz, 1H), 0.92 (s, 10H).

^13^C-NMR (400 MHz, DMSO-d_6_): **δ** 172.41, 170.43, 170.00, 163.17, 161.06, 151.92, 148.19, 146.39, 139.96, 131.64, 131.06, 130.11, 129.11, 127.89, 114.14, 113.48, 70.21, 70.17, 70.07, 69.91, 69.41, 69.39, 69.33, 67.39, 60.24, 59.19, 56.85, 40.39, 40.18, 39.97, 39.75, 39.54, 26.77, 26.73, 26.70, 16.40.

ESI-MS: C_41_H_52_N_7_O_8_S_2_F 853,33 (calculated), 854,33 [M+H]^+^ (calculated), m/z = 854,44 [M+H]^+^ (found).

LC-MS: C_41_H_52_N_7_O_8_S_2_F 853,33 (calculated), 854,33 [M+H]^+^ (calculated), m/z = 854,35 [M+H]^+^ (found).

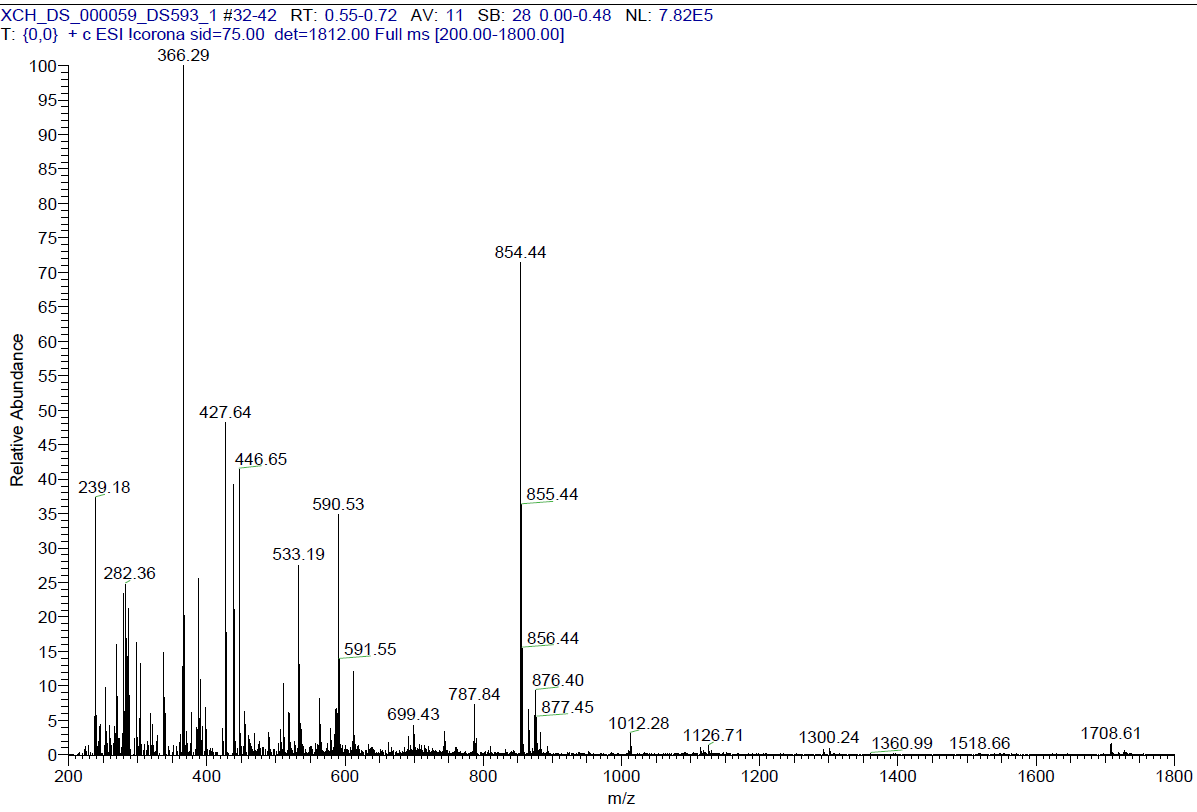


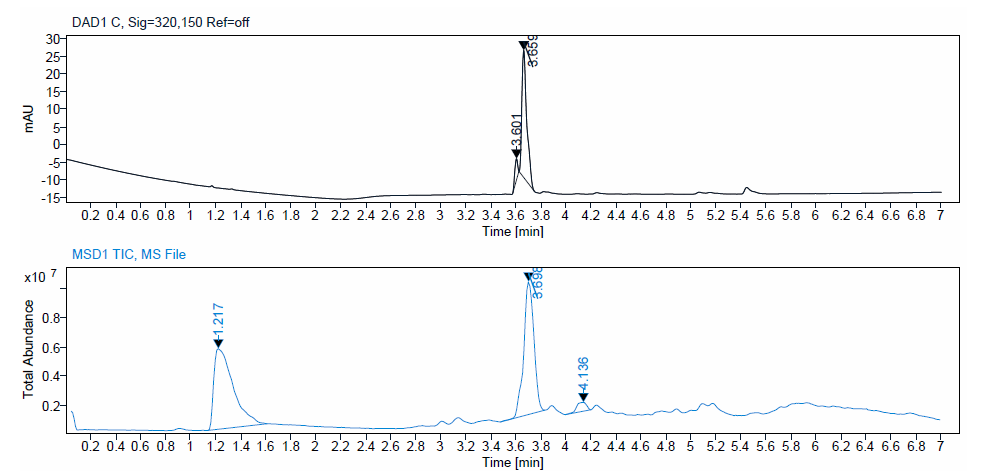


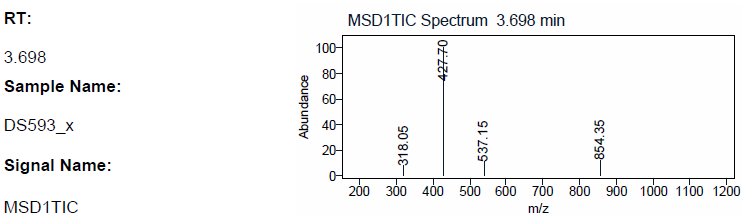


*PROTAC_V5*

^1^H-NMR (400 MHz, DMSO-d_6_): **δ** 10.48 (s, 1H), 9.00 (s, 1H), 8.56 (t, J = 6.1 Hz, 1H), 8.41 (d, J = 6.3 Hz, 1H), 7.95 (d, J = 9.4 Hz, 1H), 7.81 – 7.74 (m, 2H), 7.61 (d, J = 4.6 Hz, 1H), 7.48 – 7.32 (m, 7H), 4.55 (d, J = 9.3 Hz, 1H), 4.49 – 4.40 (m, 3H), 4.37 (s, 1H), 4.24 (dd, J = 15.8, 5.4 Hz, 1H), 4.15 (d, J = 8.1 Hz, 1H), 3.73 – 3.58 (m, 8H), 3.19 (s, 3H), 3.00 (d, J = 21.4 Hz, 2H), 2.59 (s, 2H), 2.46 (s, 4H), 2.08 (d, J = 4.3 Hz, 1H), 1.93 (ddd, J = 12.8, 8.5, 4.5 Hz, 1H), 0.96 (s, 9H).

ESI-MS: C_43_H_54_N_9_O_6_S_2_Cl 891,33 (calculated), 892,33 [M+H]^+^ (calculated), m/z = 892,47 [M+H]^+^ (found).

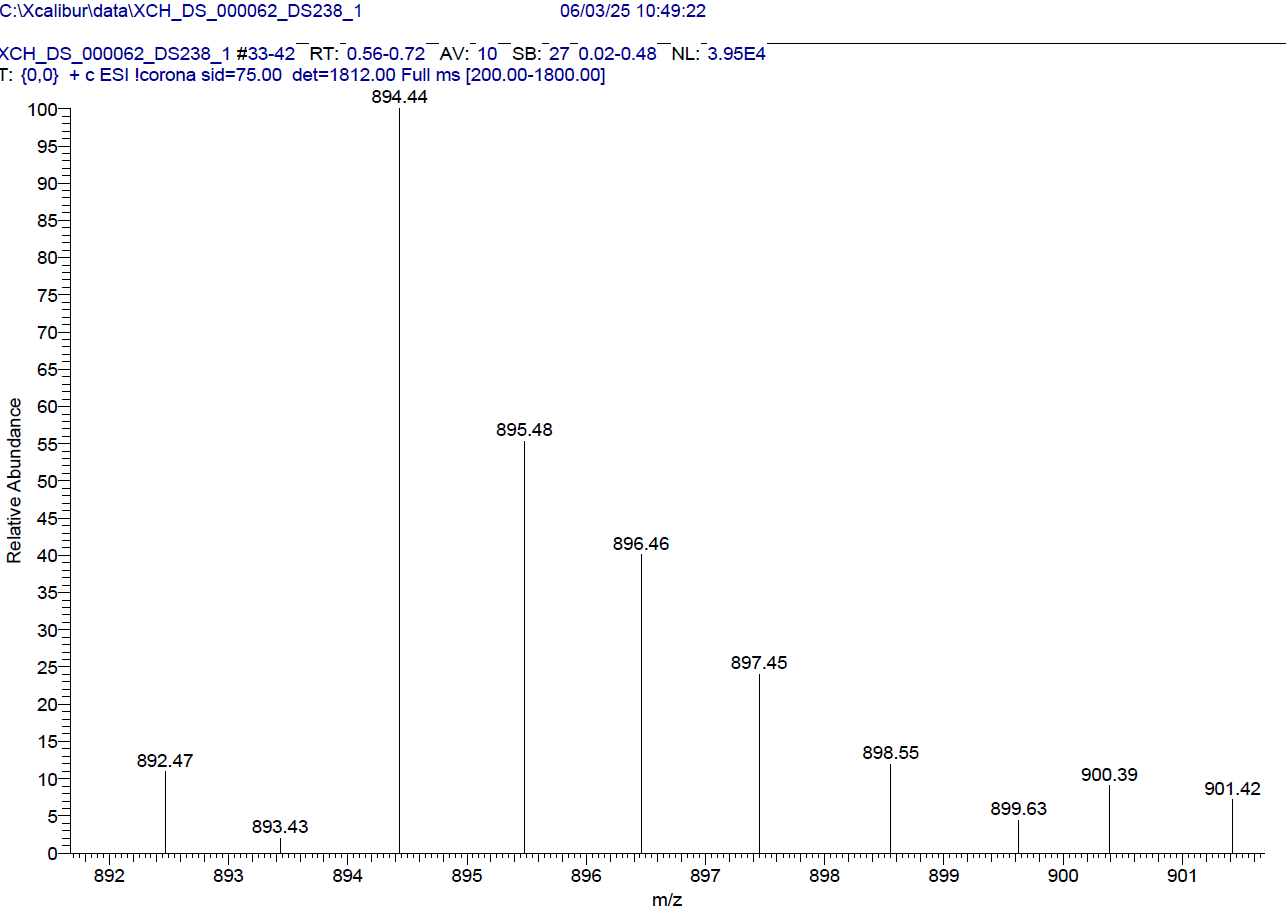


*PROTAC_V6:*

^1^H-NMR (400 MHz, DMSO-d_6_): **δ** 10.47 (s, 1H), 8.56 (t, J = 6.1 Hz, 1H), 8.15 (t, J = 5.9 Hz, 1H), 7.97 (d, J = 9.3 Hz, 1H), 7.84 (dd, J = 8.5, 2.1 Hz, 1H), 7.61 (d, J = 2.1 Hz, 1H), 7.53 (s, 1H), 7.38 (dq, J = 16.3, 8.1 Hz, 5H), 7.01 (dd, J = 8.0, 2.1 Hz, 1H), 5.12 (d, J = 3.6 Hz, 1H), 4.56 (d, J = 9.3 Hz, 1H), 4.44 (ddd, J = 10.9, 6.8, 3.2 Hz, 2H), 4.38 – 4.31 (m, 1H), 4.22 (dd, J = 15.9, 5.5 Hz, 1H), 3.65 (d, J = 3.6 Hz, 2H), 3.27 (q, J = 6.8 Hz, 2H), 2.44 (s, 3H), 2.27 (ddt, J = 46.2, 14.4, 7.5 Hz, 3H), 2.04 (ddd, J = 10.1, 7.9, 2.3 Hz, 1H), 1.90 (ddd, J = 13.0, 8.5, 4.6 Hz, 1H), 1.76 (td, J = 7.3, 3.8 Hz, 2H), 0.94 (s, 9H).

^13^C-NMR (400 MHz, DMSO-d_6_): **δ** 172.43, 172.31, 170.13, 163.00, 161.10, 151.92, 148.19, 146.70, 142.57, 139.98, 133.74, 131.64, 131.29, 130.11, 129.11, 127.89, 121.51, 116.75, 116.11, 113.92, 69.36, 60.23, 59.18, 56.89, 40.42, 40.21, 40.00, 39.78, 39.57, 26.86, 21.24, 16.41, 14.56.

ESI-MS: C_36_H_42_N_7_O_5_S_2_Cl 751,24 (calculated), 752,24 [M+H]^+^ (calculated), m/z = 752,23 [M+H]^+^ (found).

LC-MS: C_36_H_42_N_7_O_5_S_2_Cl 751,24 (calculated), 752,24 [M+H]^+^ (calculated), m/z = 752,25 [M+H]^+^ (found).

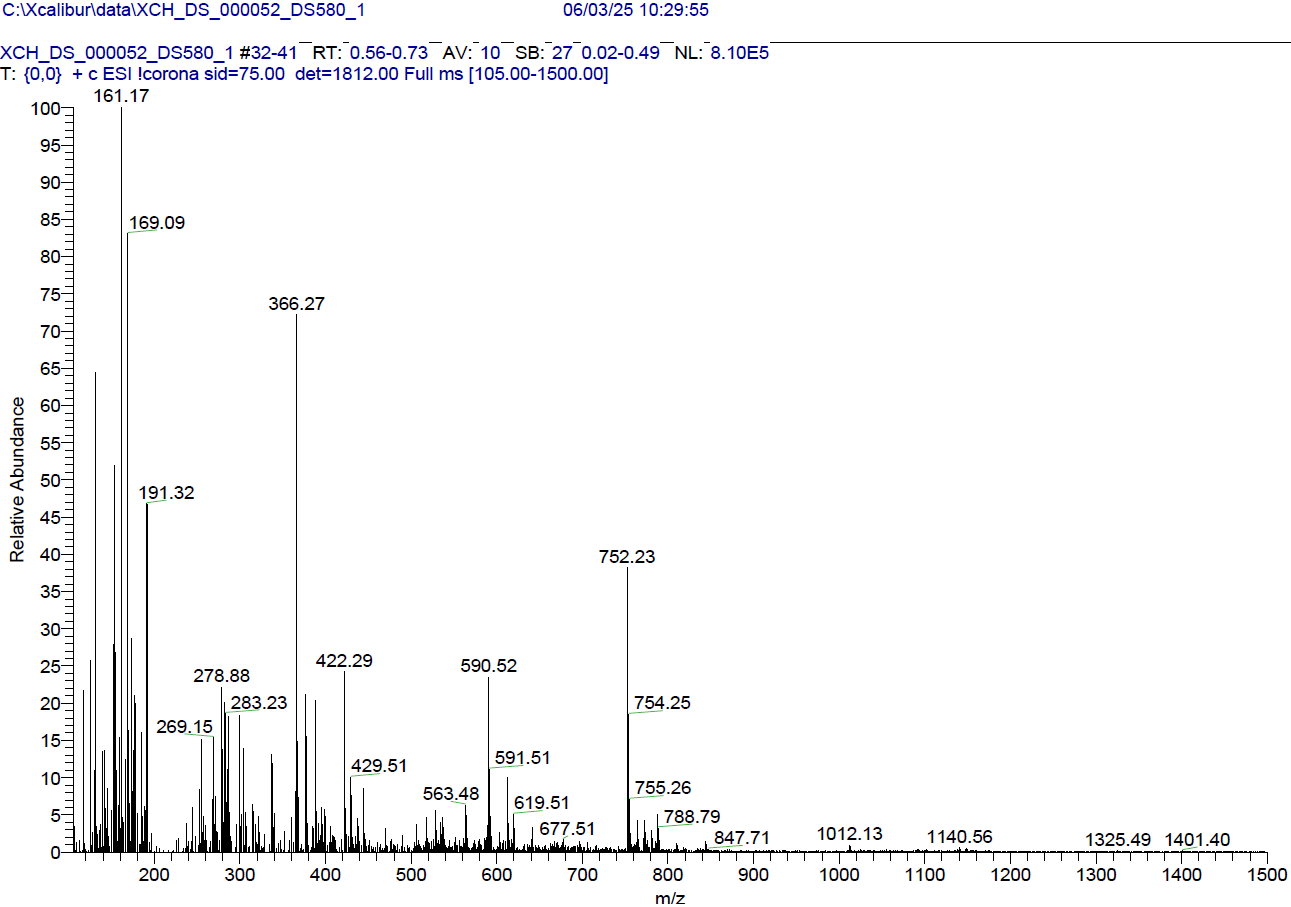


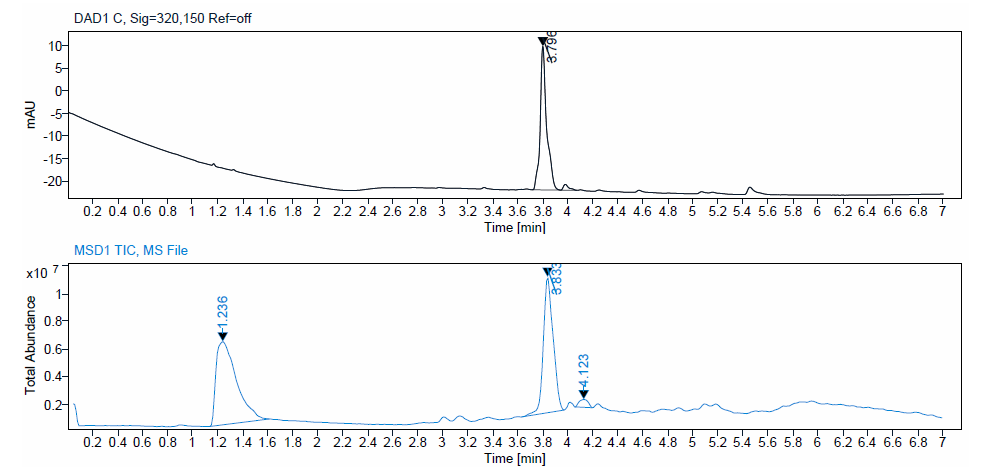


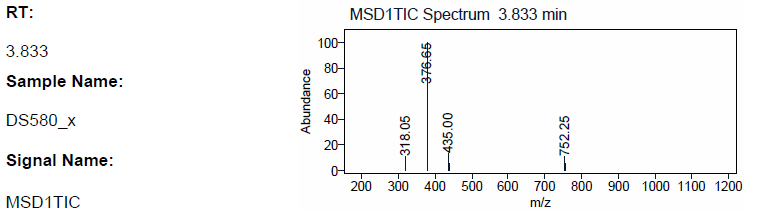


*PROTAC_V7*

^1^H-NMR (400 MHz, DMSO-d_6_): **δ** 10.49 (s, 1H), 8.99 (s, 1H), 8.56 (t, *J* = 6.0 Hz, 1H), 8.22 (t, *J* = 5.9 Hz, 1H), 7.97 (d, *J* = 9.4 Hz, 1H), 7.65 (dt, *J* = 11.7, 2.3 Hz, 1H), 7.52 (s, 1H), 7.46 – 7.37 (m, 6H), 6.81 – 6.72 (m, 1H), 4.56 (d, *J* = 9.3 Hz, 1H), 4.43 (ddd, *J* = 11.0, 6.6, 3.2 Hz, 2H), 4.35 (q, *J* = 3.2 Hz, 1H), 4.21 (dd, *J* = 15.9, 5.5 Hz, 1H), 3.65 (d, *J* = 3.2 Hz, 2H), 3.26 (t, *J* = 6.8 Hz, 3H), 2.44 (s, 3H), 2.32 (dt, *J* = 15.0, 7.7 Hz, 1H), 2.21 (dt, *J* = 14.4, 7.2 Hz, 1H), 2.08 – 2.00 (m, 1H), 1.95 – 1.86 (m, 1H), 1.76 (td, *J* = 7.3, 3.2 Hz, 2H), 0.94 (s, 9H).

^13^C-NMR (400 MHz, DMSO-d_6_): **δ** 172.44, 172.35, 170.14, 163.07, 161.13, 151.94, 148.16, 146.74, 139.98, 131.65, 130.09, 129.11, 127.89, 113.91, 113.50, 108.36, 69.36, 60.24, 56.90, 40.39, 40.18, 39.97, 39.75, 39.54, 26.84, 26.27, 21.23, 16.39, 14.55.

ESI-MS: C_36_H_42_N_7_O_5_S_2_Cl 735,27 (calculated), 736,27 [M+H]^+^ (calculated), m/z = 736,26 [M+H]^+^ (found).

LC-MS: C_36_H_42_N_7_O_5_S_2_Cl 735,27 (calculated), 736,27 [M+H]^+^ (calculated), m/z = 736,25 [M+H]^+^ (found).

*
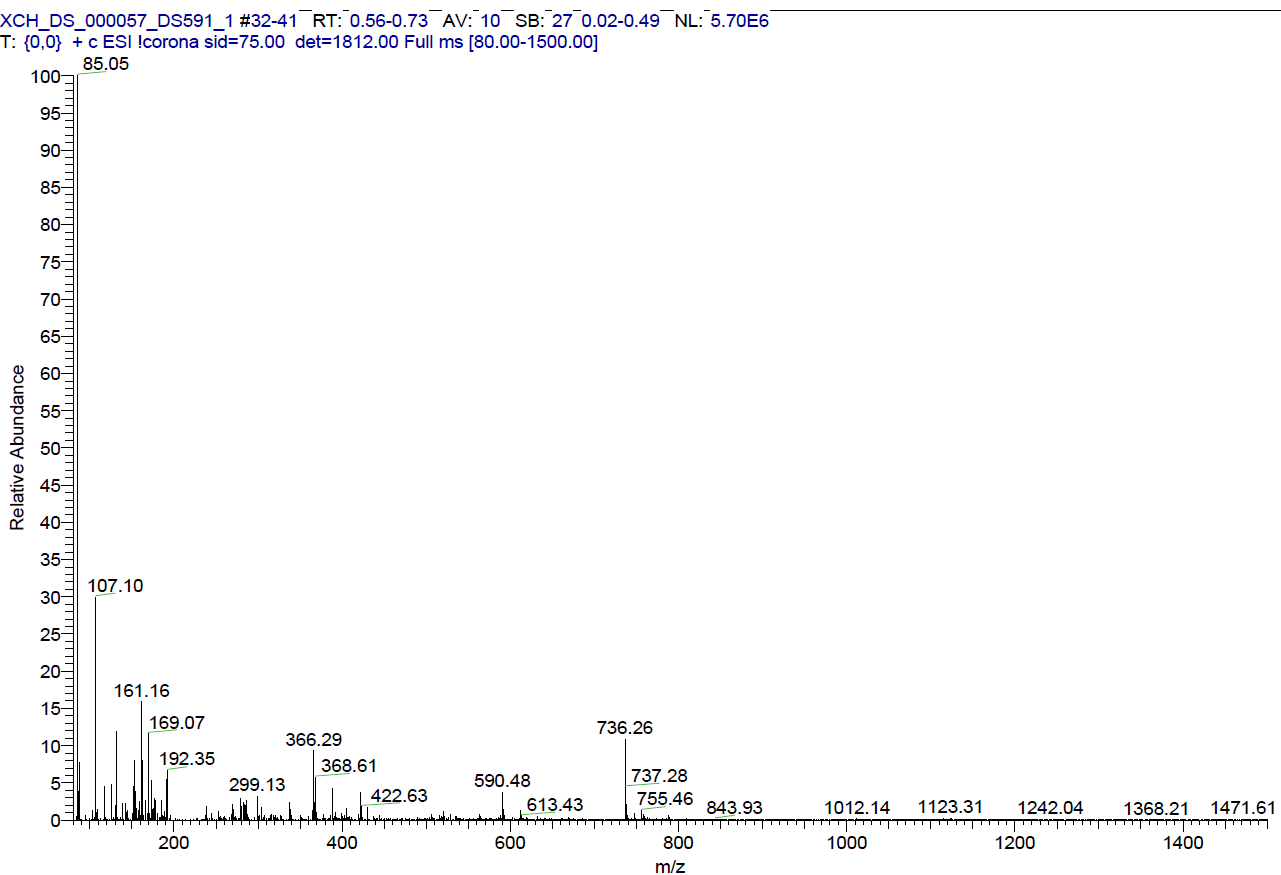
*

*
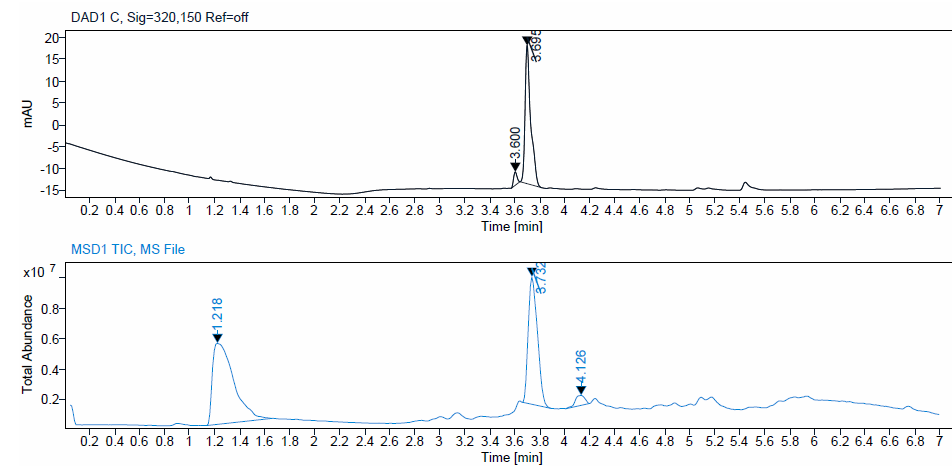
*

*
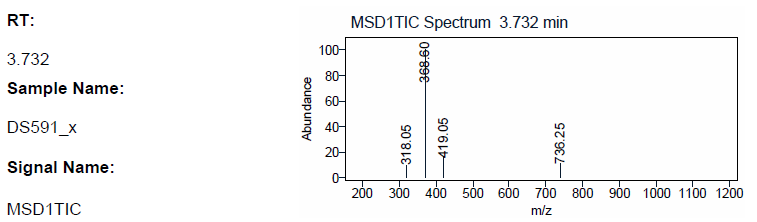
*

*PROTAC_V9*

^1^H-NMR (300 MHz, DMSO-d_6_): **δ** 10.59 (s, 1H), 9.00 (s, 1H), 8.48 (d, J = 7.7 Hz, 1H), 7.79 (d, J = 9.7 Hz, 1H), 7.64 – 7.57 (m, 3H), 7.49 – 7.36 (m, 7H), 5.15 (s, 1H), 4.94 (t, J = 7.2 Hz, 1H), 4.65 (d, J = 9.7 Hz, 1H), 4.49 (t, J = 8.4 Hz, 1H), 4.32 (s, 1H), 3.66 (s, 2H), 2.47 (d, J = 4.0 Hz, 3H), 2.09 (t, J = 10.5 Hz, 1H), 1.86 – 1.74 (m, 1H), 1.41 (d, J = 7.0 Hz, 3H), 1.03 (s, 10H).

^13^C-NMR (300 MHz, DMSO-d_6_): **δ** 170.91, 169.56, 163.65, 163.34, 145.27, 130.18, 129.37, 129.32, 129.28, 129.19, 126.88, 126.81, 126.76, 125.80, 119.13, 114.12, 40.59, 40.31, 40.03, 39.74, 39.46, 26.67, 26.63, 16.47.

ESI-MS: C_33_H_37_N_6_O_4_S_2_Cl 680,20 (calculated), 681,20 [M+H]^+^ (calculated), m/z = 681,11 [M+H]^+^ (found).

LC-MS: C_33_H_37_N_6_O_4_S_2_Cl 680,20 (calculated), 681,20 [M+H]^+^ (calculated), m/z = 681,25 [M+H]^+^ (found).

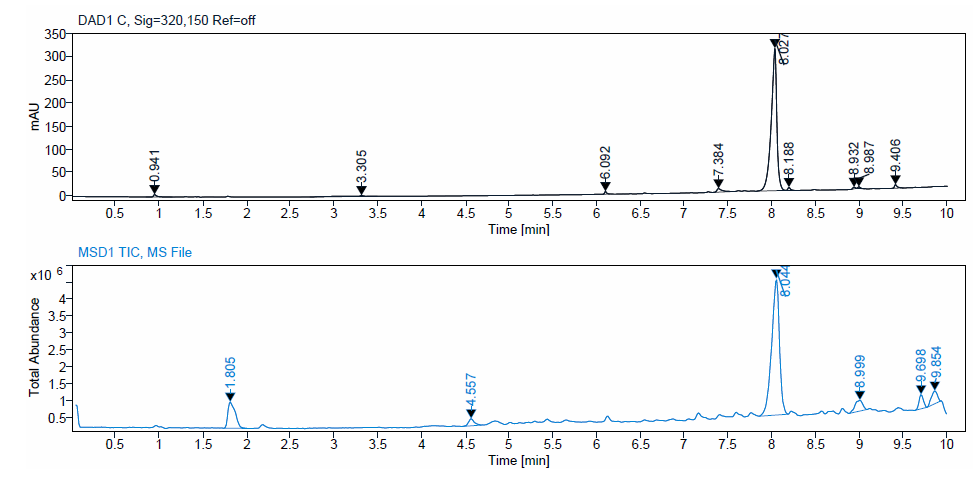


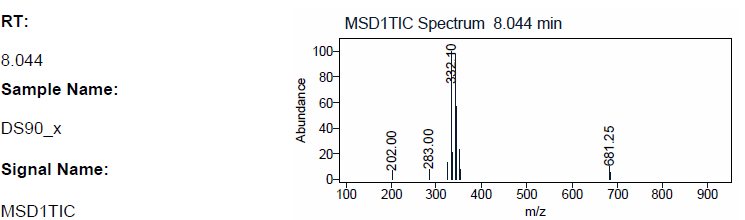


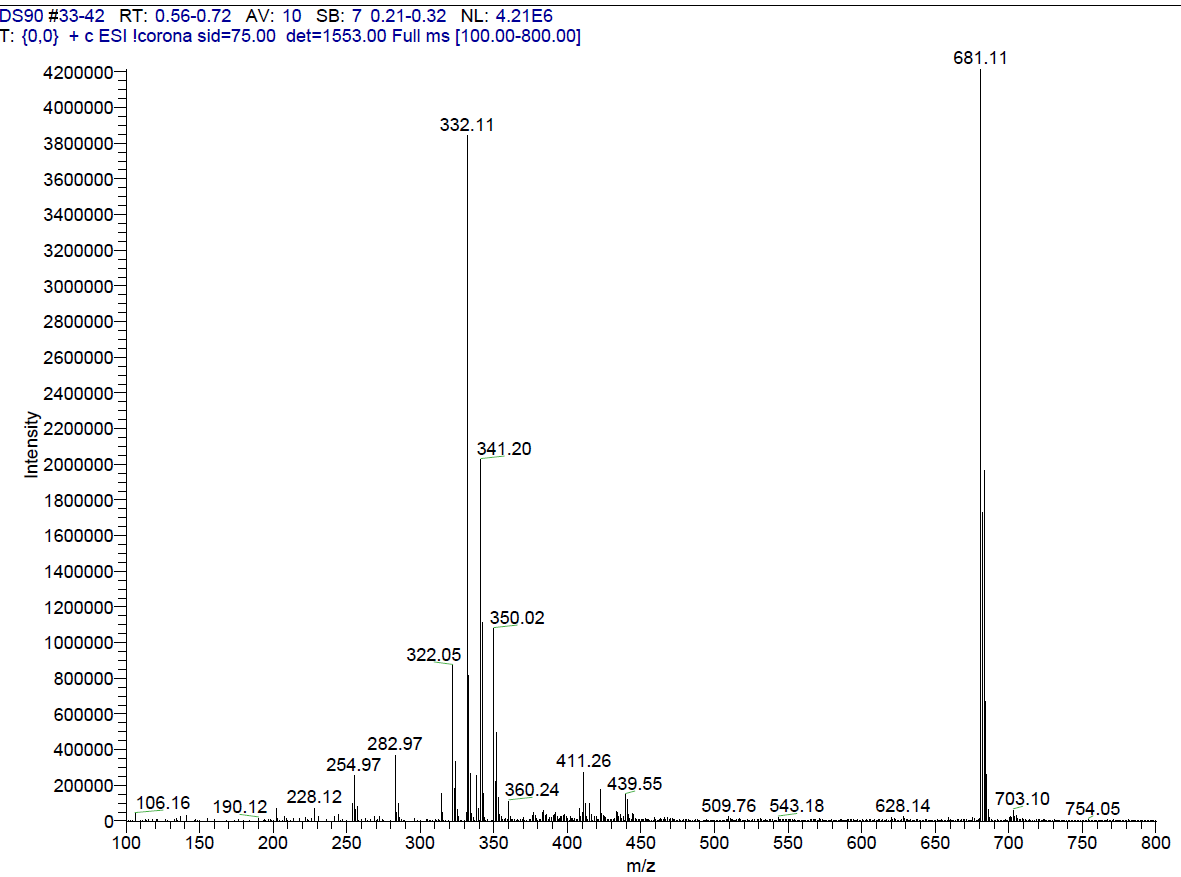


*PROTAC_V10:*

^1^H-NMR (400 MHz, DMSO-d_6_): **δ** 10.44 (s, 1H), 8.98 (s, 1H), 8.56 (t, *J* = 6.1 Hz, 1H), 8.08 (t, *J* = 6.1 Hz, 1H), 7.87 (d, *J* = 9.3 Hz, 1H), 7.77 – 7.73 (m, 2H), 7.49 (s, 1H), 7.44 – 7.32 (m, 7H), 5.12 (d, *J* = 3.6 Hz, 1H), 4.55 (d, *J* = 9.3 Hz, 1H), 4.47 – 4.39 (m, 2H), 4.35 (s, 1H), 4.21 (dd, *J* = 15.9, 5.4 Hz, 1H), 3.70 – 3.62 (m, 2H), 3.26 (d, *J* = 6.3 Hz, 3H), 2.44 (s, 3H), 2.31 (d, *J* = 14.6 Hz, 1H), 2.22 – 2.12 (m, 1H), 2.06 (s, 1H), 1.95 – 1.84 (m, 1H), 1.52 (s, 3H), 1.31 – 1.20 (m, 3H), 0.94 (s, 10H).

^13^C-NMR (400 MHz, DMSO-d_6_): **δ** 172.48, 172.41, 170.16, 163.16, 160.97, 151.92, 148.18, 146.62, 140.13, 139.97, 131.63, 130.10, 129.25, 129.10, 127.88, 125.18, 119.14, 119.06, 113.60, 69.34, 59.17, 56.85, 40.42, 40.27, 40.21, 39.99, 39.78, 39.57, 26.86, 16.41.

ESI-MS: C_37_H_44_N_7_O_5_S_2_Cl 765,25 (calculated), 766,25 [M+H]^+^ (calculated), m/z = 766,20 [M+H]^+^ (found).

LC-MS: C_37_H_44_N_7_O_5_S_2_Cl 765,25 (calculated), 766,25 [M+H]^+^ (calculated), m/z = 766,25 [M+H]^+^ (found).

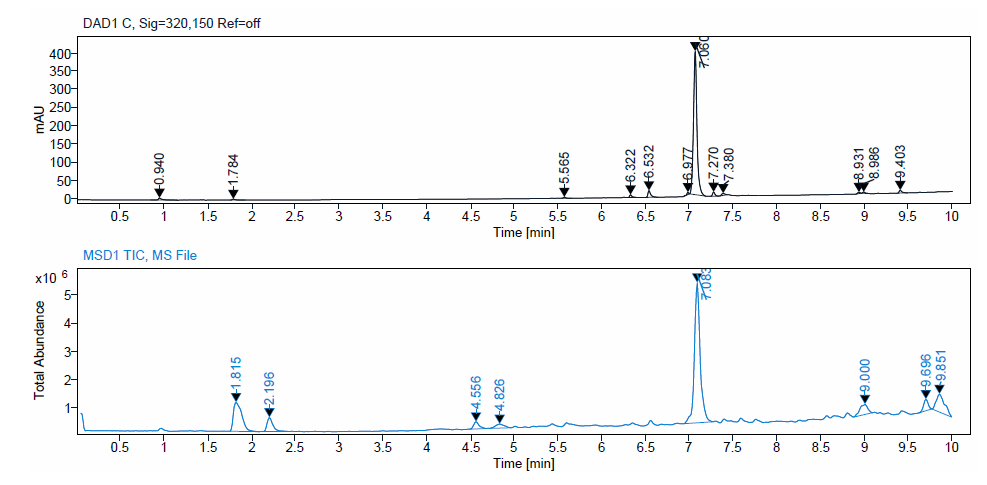


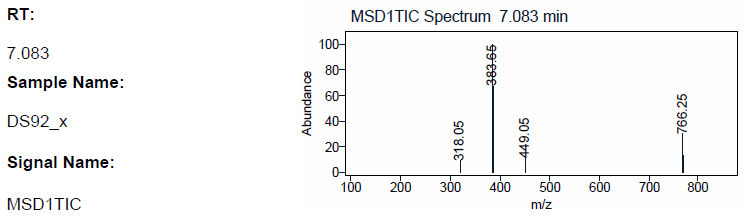


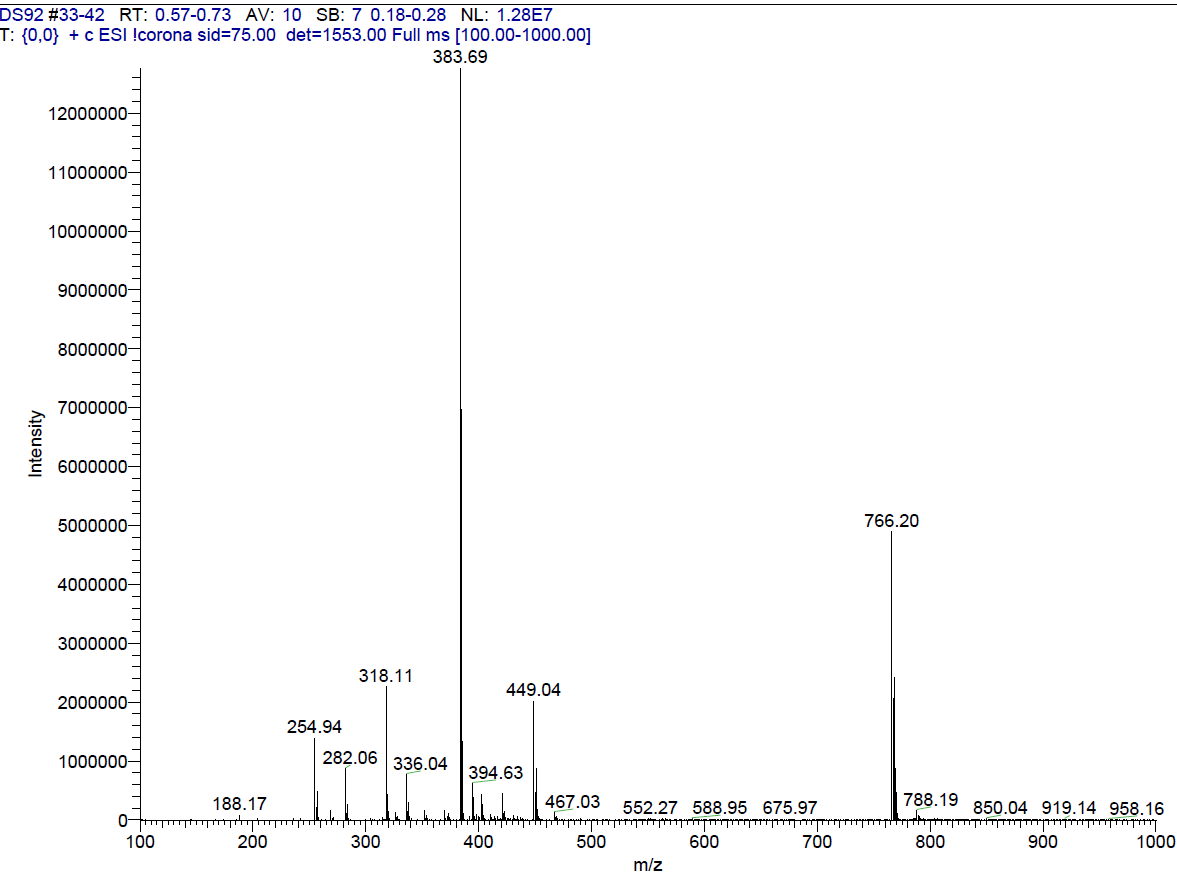


*PROTAC_V11:*

^1^H-NMR (400 MHz, DMSO-d_6_): **δ** 10.46 (s, 1H), 8.98 (s, 1H), 8.57 (t, *J* = 6.1 Hz, 1H), 8.03 (t, *J* = 5.9 Hz, 1H), 7.95 (d, *J* = 9.4 Hz, 1H), 7.73 (dd, *J* = 8.3, 3.4 Hz, 2H), 7.52 (s, 1H), 7.44 – 7.34 (m, 7H), 4.56 (d, *J* = 9.4 Hz, 1H), 4.43 (td, *J* = 7.3, 3.0 Hz, 2H), 4.35 (s, 1H), 4.22 (dd, *J* = 15.9, 5.5 Hz, 1H), 3.71 – 3.59 (m, 4H), 3.57 – 3.38 (m, 5H), 2.57 (dd, *J* = 14.4, 7.0 Hz, 1H), 2.44 (s, 4H), 2.09 – 2.01 (m, 1H), 1.90 (ddd, *J* = 13.0, 8.5, 4.5 Hz, 1H), 0.92 (s, 9H).

^13^C-NMR (400 MHz, DMSO-d_6_): **δ** 172.39, 163.23, 161.10, 151.92, 140.11, 139.97, 131.64, 129.27, 129.11, 127.89, 125.26, 119.12, 118.83, 69.36, 69.07, 67.27, 59.19, 56.89, 56.86, 40.42, 40.21, 40.00, 39.79, 39.58, 26.79, 16.41.

ESI-MS: C_37_H_44_N_7_O_6_S_2_Cl 781,25 (calculated), 782,25 [M+H]^+^ (calculated), m/z = 782,31 [M+H]^+^ (found).

LC-MS: C_37_H_44_N_7_O_6_S_2_Cl 781,25 (calculated), 782,25 [M+H]^+^ (calculated), m/z = 782,25 [M+H]^+^ (found).

*
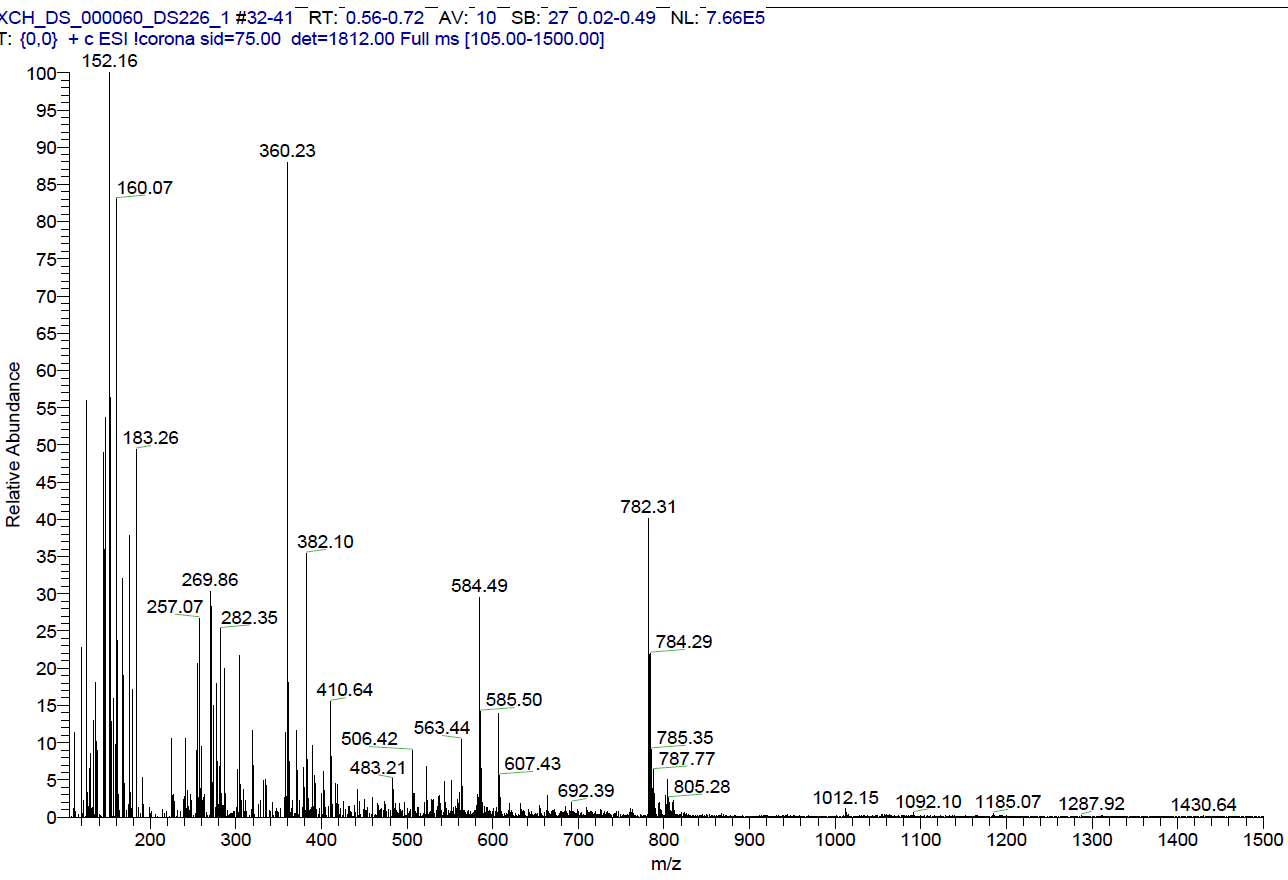
*

*
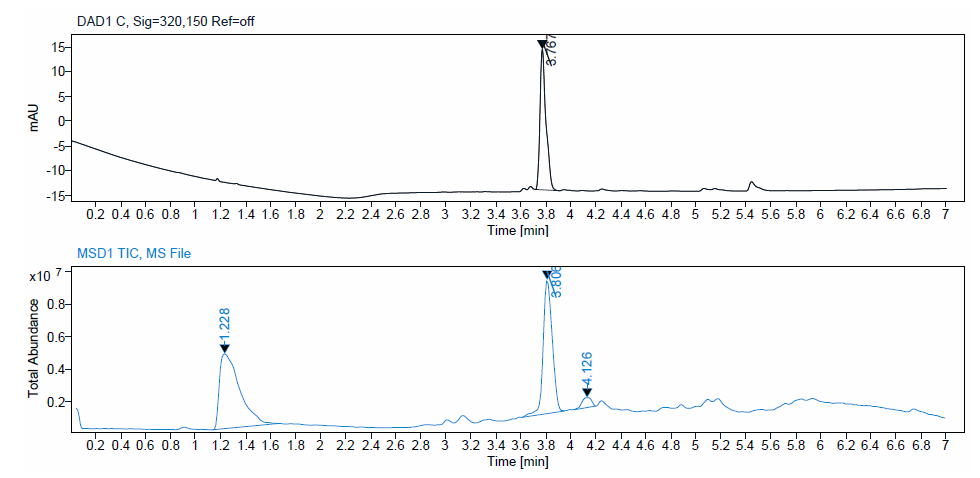
*

*
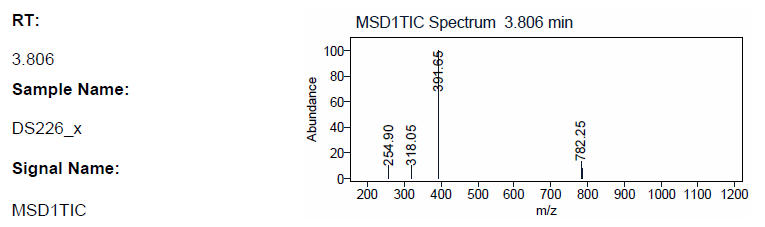
*

*PROTAC_C1:*

^1^H-NMR (300 MHz, DMSO-d_6_): **δ** 11.01 (s, 1H), 10.49 (s, 1H), 9.81 (s, 1H), 7.96 (t, *J* = 5.8 Hz, 1H), 7.81 (dd, *J* = 6.7, 2.2 Hz, 1H), 7.72 (dd, *J* = 8.1, 2.2 Hz, 1H), 7.63 (t, *J* = 2.1 Hz, 1H), 7.58 – 7.44 (m, 3H), 7.33 (t, *J* = 8.1 Hz, 1H), 7.01 (dd, *J* = 7.9, 2.1 Hz, 1H), 5.14 (dd, *J* = 13.2, 5.1 Hz, 1H), 4.44 – 4.24 (m, 2H), 3.68 (t, *J* = 6.3 Hz, 2H), 3.58 – 3.39 (m, 12H), 2.99 – 2.83 (m, 1H), 2.60 (q, *J* = 6.7 Hz, 3H), 2.42 – 2.24 (m, 1H), 2.10 – 1.96 (m, 1H).

^13^C-NMR (300 MHz, DMSO-d_6_): **δ** 173.32, 171.53, 169.79, 168.28, 163.14, 161.03, 146.35, 142.52, 134.16, 134.12, 133.78, 133.14, 131.21, 129.12, 121.60, 116.75, 116.05, 114.20, 70.99, 70.23, 70.18, 70.07, 69.38, 67.04, 51.99, 49.08, 40.42, 40.20, 39.99, 39.78, 39.57, 23.13.

ESI-MS: C_32_H_35_N_6_O_8_S_2_Cl 698,19 (calculated), 699,19 [M+H]^+^ (calculated), m/z = 699,15 [M+H]^+^ (found).

LC-MS: C_32_H_35_N_6_O_8_S_2_Cl 698,19 (calculated), 699,19 [M+H]^+^ (calculated), m/z = 699,15 [M+H]^+^ (found).

*
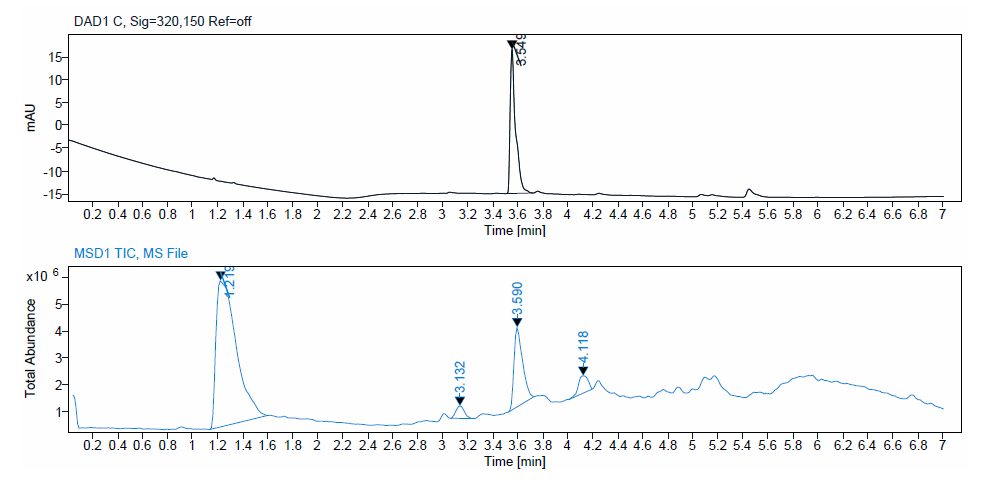
*

*
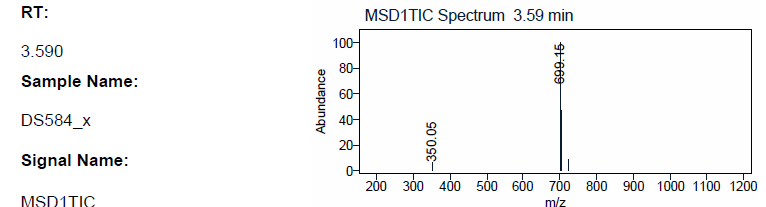
*

*
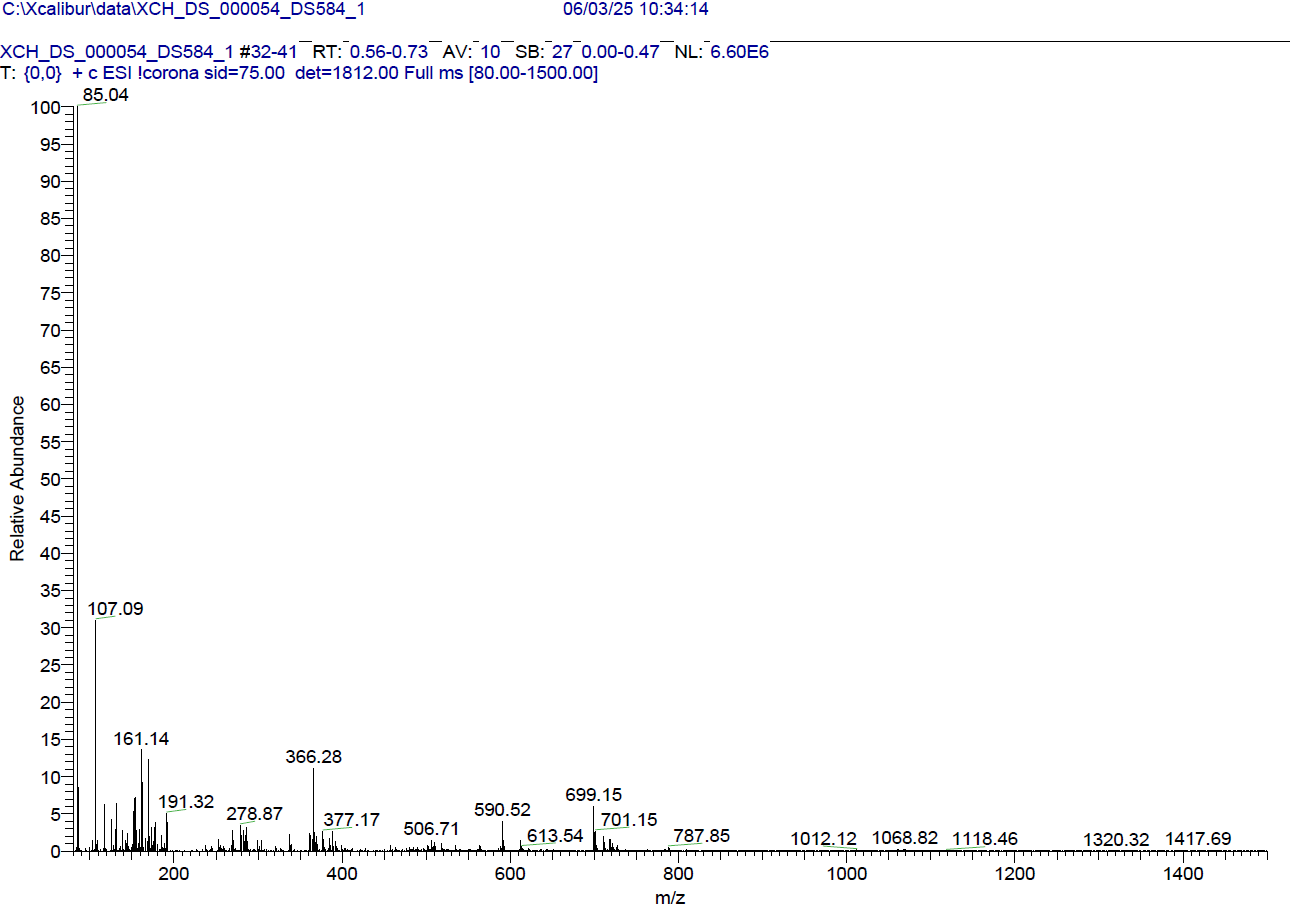
*

*PROTAC_C2:*

^1^H-NMR (400 MHz, DMSO-d_6_): **δ** 11.01 (s, 1H), 10.49 (s, 1H), 9.84 (s, 1H), 8.23 (t, *J* = 6.1 Hz, 1H), 7.81 (dd, *J* = 7.7, 1.3 Hz, 1H), 7.66 (dt, *J* = 11.7, 2.3 Hz, 1H), 7.53 – 7.40 (m, 4H), 7.36 – 7.27 (m, 1H), 6.77 (td, *J* = 8.4, 2.5 Hz, 1H), 5.13 (dd, *J* = 13.3, 5.1 Hz, 1H), 4.44 – 4.30 (m, 2H), 3.43 (d, *J* = 5.9 Hz, 2H), 2.91 (ddd, *J* = 17.9, 13.5, 5.4 Hz, 1H), 2.60 (d, *J* = 14.7 Hz, 1H), 2.46 – 2.29 (m, 3H), 2.06 – 1.98 (m, 1H), 1.93 – 1.84 (m, 2H).

^13^C-NMR (400 MHz, DMSO-d_6_): **δ** 173.35, 171.61, 171.53, 168.32, 163.09, 161.20, 146.67, 134.21, 133.09, 129.02, 119.46, 114.00, 113.49, 51.99, 40.39, 40.18, 39.97, 39.76, 39.55, 25.85, 23.08.

ESI-MS: C_27_H_25_N_6_O_5_SF 564,16 (calculated), 565,16 [M+H]^+^ (calculated), m/z = 565,07 [M+H]^+^ (found).

LC-MS: C_27_H_25_N_6_O_5_SF 564,16 (calculated), 565,16 [M+H]^+^ (calculated), m/z = 565,10 [M+H]^+^ (found).

*
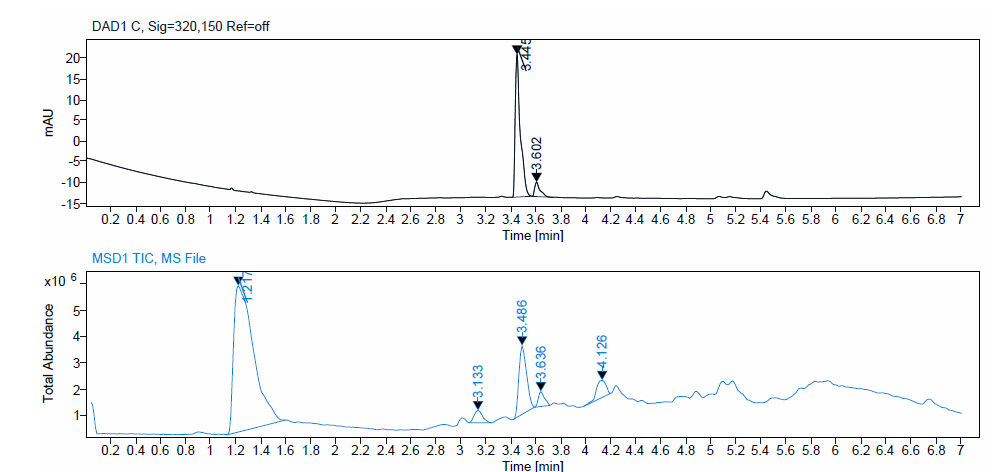
*

*
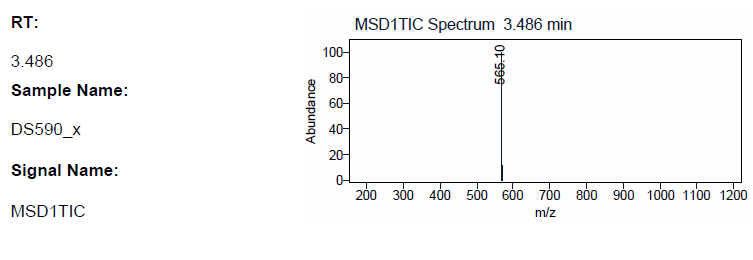
*

*
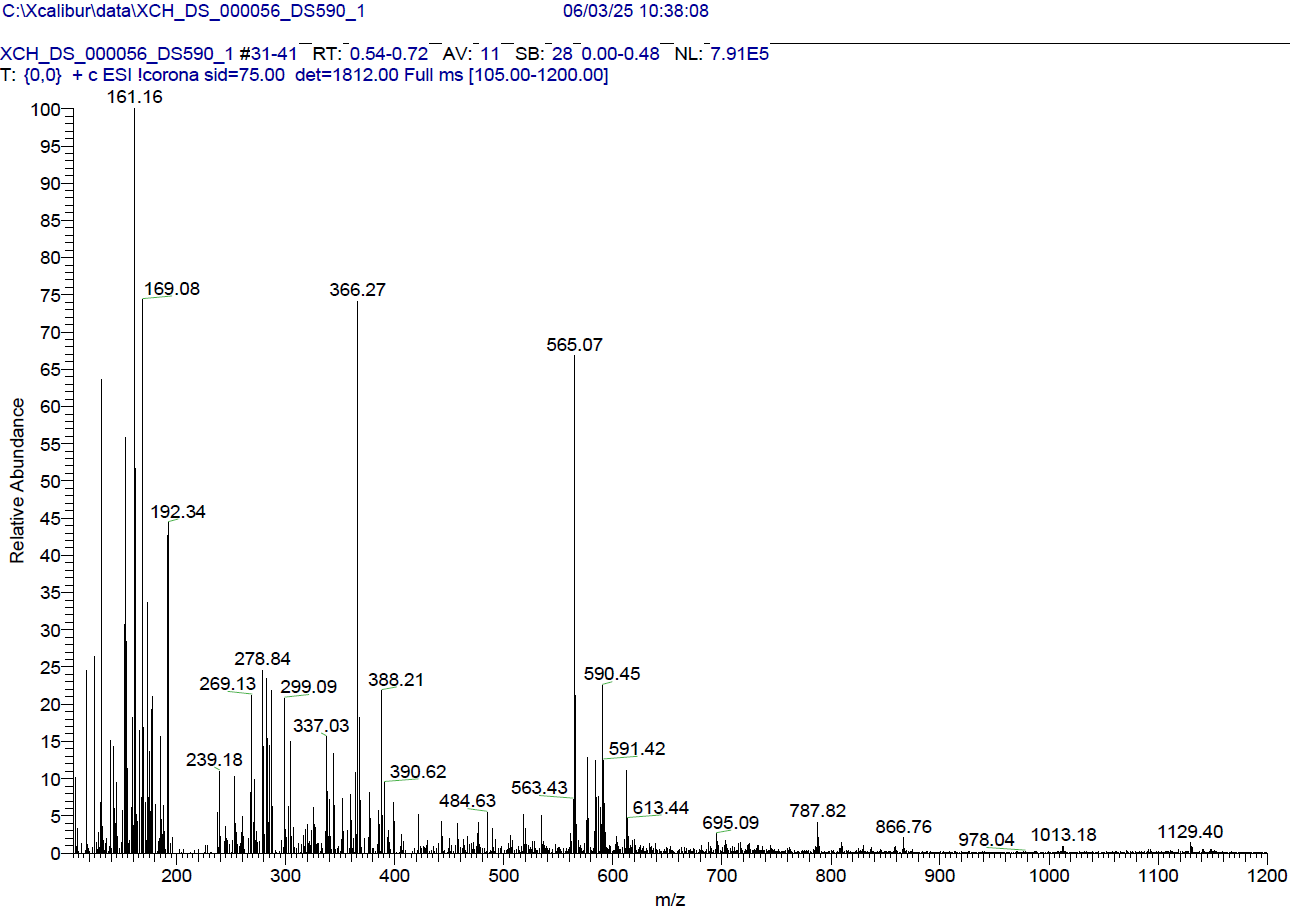
*

*PROTAC_C3*

^1^H-NMR (400 MHz, DMSO-d_6_): **δ** 11.01 (s, 1H), 10.42 (s, 1H), 9.76 (s, 1H), 8.09 (t, *J* = 6.1 Hz, 1H), 7.81 (dd, *J* = 7.3, 1.7 Hz, 1H), 7.76 – 7.71 (m, 2H), 7.51 – 7.44 (m, 3H), 7.36 – 7.29 (m, 2H), 5.14 (dd, *J* = 13.3, 5.1 Hz, 1H), 4.44 – 4.30 (m, 2H), 3.28 (q, *J* = 6.8 Hz, 2H), 2.89 (td, *J* = 7.4, 4.8 Hz, 1H), 2.36 (q, *J* = 7.4 Hz, 3H), 2.07 – 1.96 (m, 1H), 1.61 (dp, *J* = 30.4, 7.3 Hz, 4H), 1.37 (q, *J* = 7.8 Hz, 2H).

^13^C-NMR (400 MHz, DMSO-d_6_): **δ** 173.33, 173.23, 171.78, 171.54, 168.29, 163.16, 160.97, 146.65, 140.12, 134.25, 133.12, 129.24, 129.05, 125.18, 119.14, 40.42, 40.20, 39.99, 39.78, 39.57, 25.69, 25.36.

ESI-MS: C_29_H_29_N_6_O_5_SCl 608,16 (calculated), 609,16 [M+H]^+^ (calculated), m/z = 609,06 [M+H]^+^ (found).

LC-MS: C_29_H_29_N_6_O_5_SCl 608,16 (calculated), 609,16 [M+H]^+^ (calculated), m/z = 609,20 [M+H]^+^ (found).

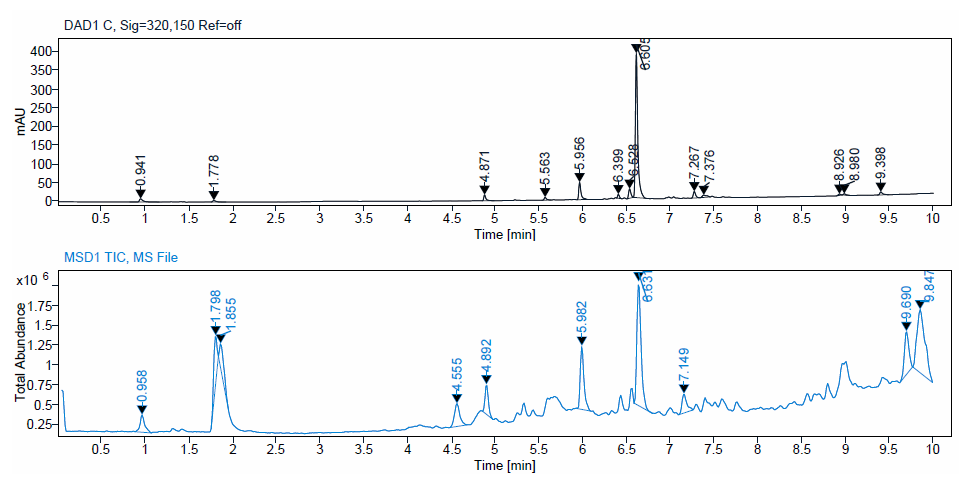


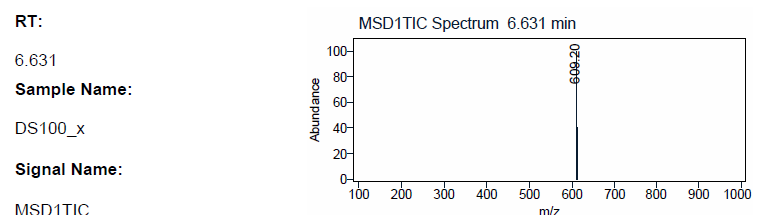


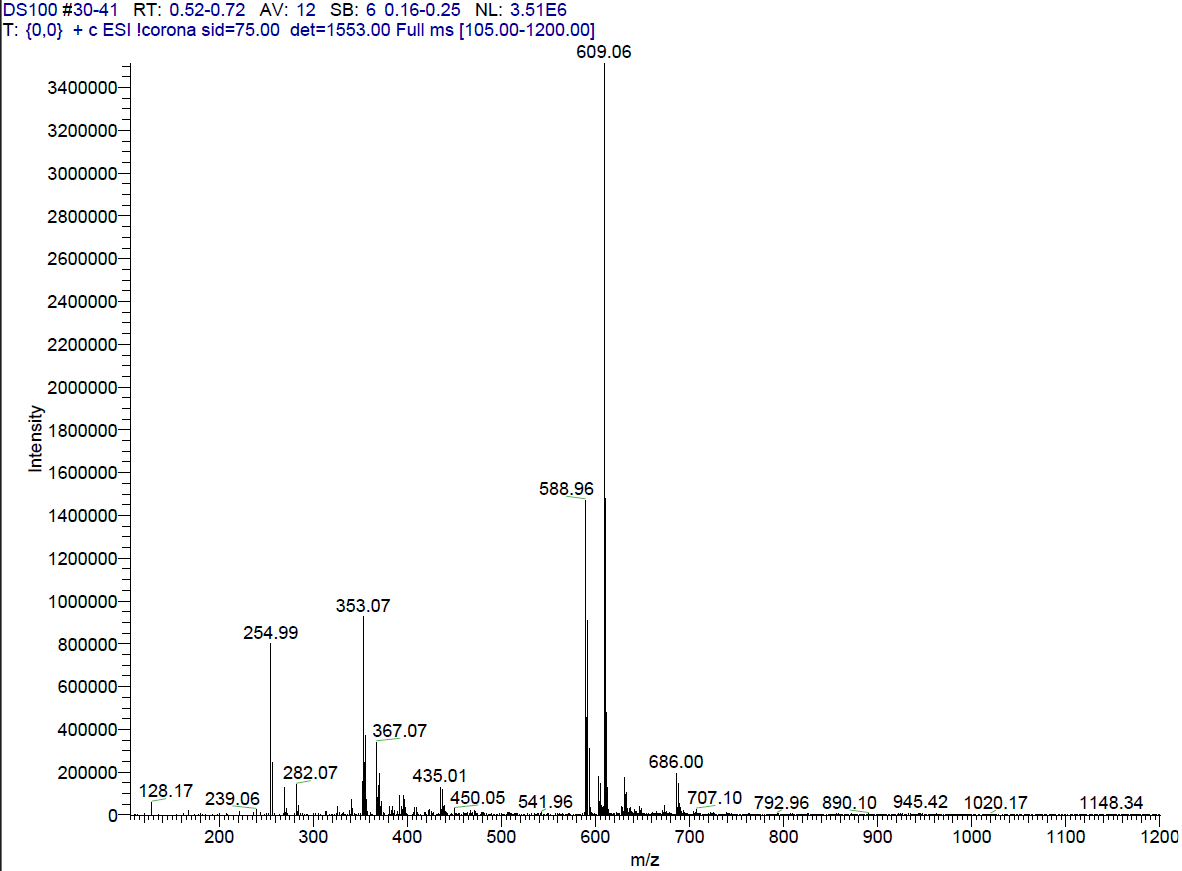


*PROTAC_C4:*

^1^H-NMR (300 MHz, DMSO-d_6_): **δ** 11.09 (s, 1H), 10.47 (s, 1H), 8.04 (t, J = 5.5 Hz, 1H), 7.77 – 7.66 (m, 3H), 7.54 (s, 1H), 7.40 (d, J = 2.2 Hz, 1H), 7.32 (dd, J = 8.7, 2.3 Hz, 1H), 7.29 – 7.17 (m, 2H), 5.09 (dd, J = 12.6, 5.4 Hz, 1H), 3.47 (d, J = 7.1 Hz, 6H), 3.00 – 2.82 (m, 2H), 2.75 (d, J = 0.7 Hz, 1H), 2.62 (dt, J = 15.2, 5.0 Hz, 8H), 2.09 – 1.97 (m, 1H), 1.25 (s, 1H).

^13^C-NMR (300 MHz, DMSO-d_6_): **δ** 173.28, 170.50, 168.04, 167.50, 163.27, 160.76, 155.86, 146.39, 140.11, 134.41, 129.23, 129.21, 129.17, 125.40, 125.27, 119.16, 118.98, 118.44, 113.83, 108.47, 52.50, 49.27, 47.72, 47.68, 47.65, 47.63, 40.32, 40.03, 39.75, 39.47.

ESI-MS: C_29_H_28_N_7_O_5_SCl 621,16 (calculated), 622,16 [M+H]^+^ (calculated), m/z = 622,07 [M+H]^+^ (found).

LC-MS: C_29_H_28_N_7_O_5_SCl 621,16 (calculated), 622,16 [M+H]^+^ (calculated), m/z = 622,10 [M+H]^+^ (found).

*
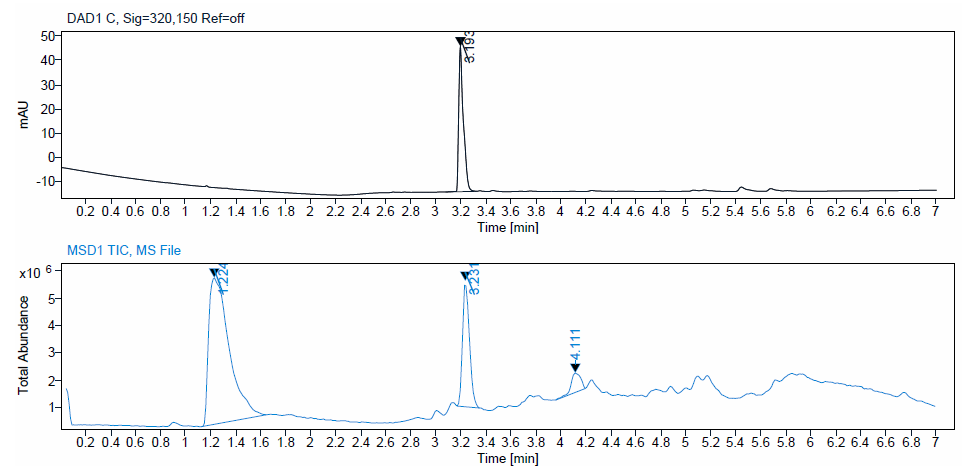
*

*
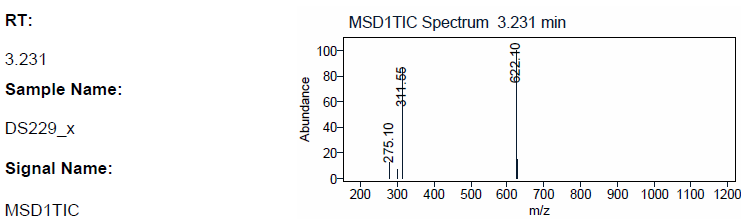
*

*
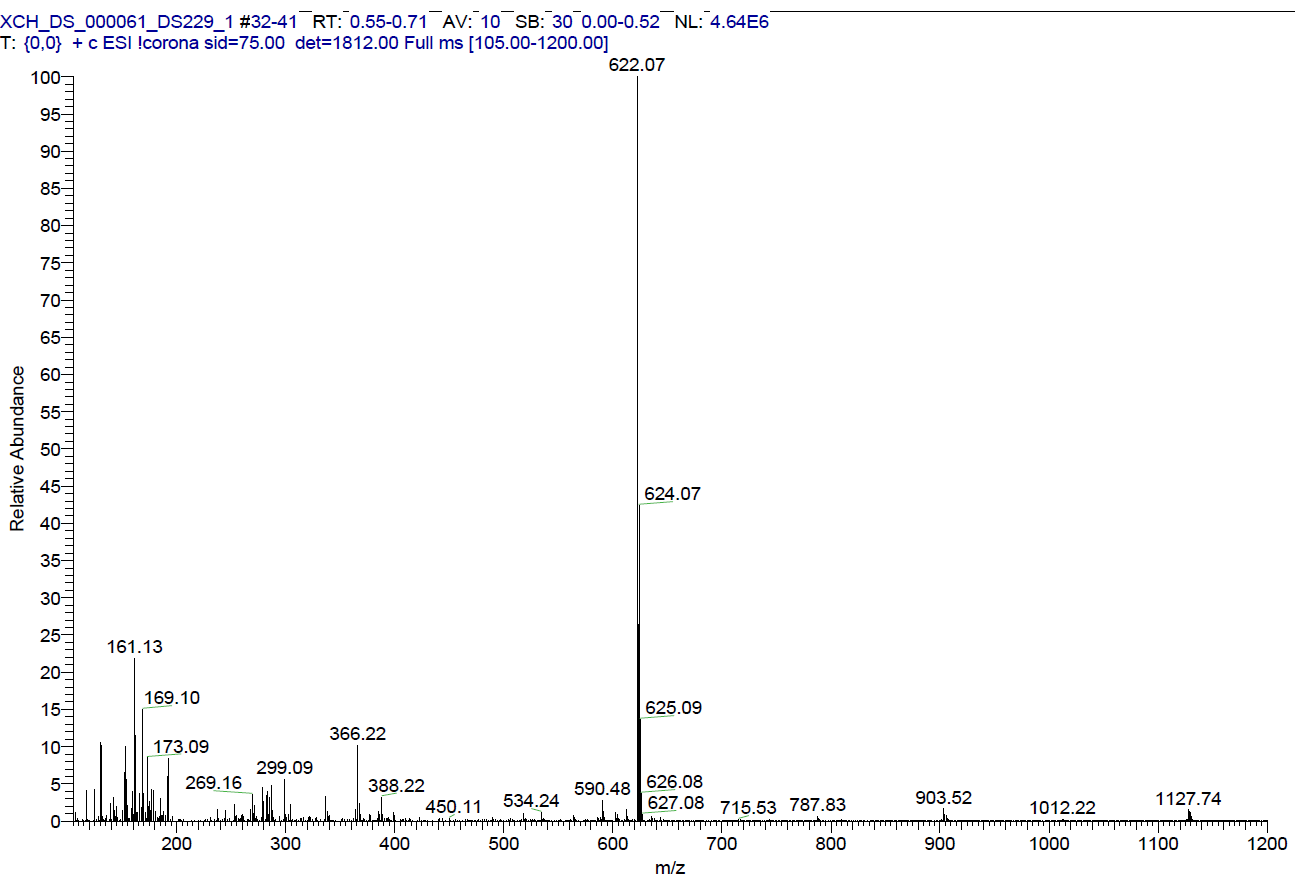
*

*PROTAC_C5*

^1^H-NMR (400 MHz, DMSO-d_6_): **δ** 11.01 (s, 1H), 10.44 (s, 1H), 9.83 (s, 1H), 8.05 (t, *J* = 5.9 Hz, 1H), 7.81 (dd, *J* = 7.7, 1.2 Hz, 1H), 7.77 – 7.68 (m, 2H), 7.55 – 7.42 (m, 3H), 7.37 – 7.29 (m, 2H), 5.15 (dd, *J* = 13.3, 5.1 Hz, 1H), 4.46 – 4.29 (m, 2H), 3.78 (t, *J* = 6.3 Hz, 2H), 3.60 (s, 2H), 3.46 (q, *J* = 6.0 Hz, 2H), 3.00 – 2.86 (m, 1H), 2.64 (q, *J* = 5.2 Hz, 3H), 2.43 – 2.28 (m, 1H), 2.08 – 1.98 (m, 1H).

^13^C-NMR (400 MHz, DMSO-d_6_): **δ** 172.81, 171.02, 169.28, 160.63, 145.83, 139.59, 133.60, 128.74, 128.57, 124.76, 119.06, 118.62, 113.38, 68.69, 66.36, 51.51, 46.42, 39.94, 39.73, 39.53, 39.32, 39.11.

ESI-MS: C_28_H_27_N_6_O_6_SCl 610,14 (calculated), 611,14 [M+H]^+^ (calculated), m/z = 611,04 [M+H]^+^ (found).

LC-MS: C_28_H_27_N_6_O_6_SCl 610,14 (calculated), 611,14 [M+H]^+^ (calculated), m/z = 611,15 [M+H]^+^ (found).

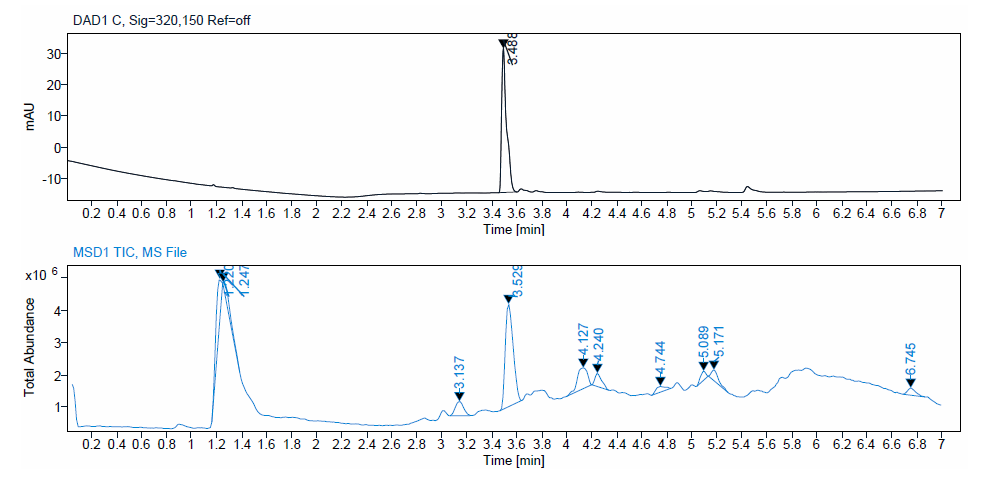


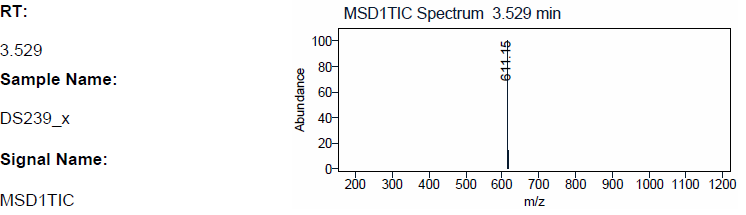


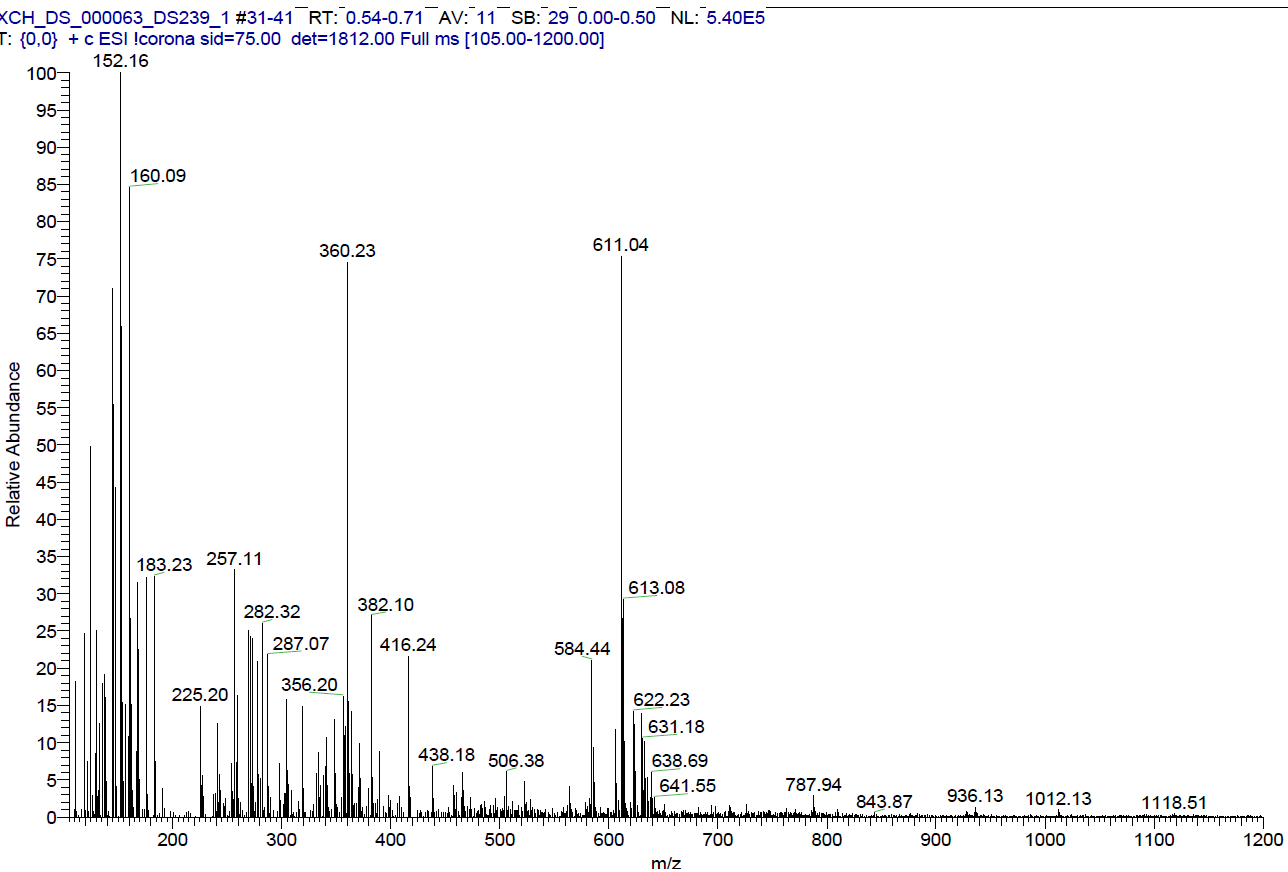


*PROTAC_C6:*

^1^H-NMR (400 MHz, DMSO-d_6_): **δ** 11.01 (s, 1H), 10.46 (s, 1H), 9.83 (s, 1H), 8.16 (t, *J* = 6.0 Hz, 1H), 7.85 – 7.78 (m, 2H), 7.60 (t, *J* = 2.1 Hz, 1H), 7.54 – 7.42 (m, 3H), 7.33 (t, *J* = 8.1 Hz, 1H), 7.03 – 6.98 (m, 1H), 5.14 (dd, *J* = 13.3, 5.1 Hz, 1H), 4.46 – 4.28 (m, 2H), 4.03 (q, *J* = 7.1 Hz, 1H), 3.41 – 3.34 (m, 3H), 2.92 (ddd, *J* = 17.2, 13.6, 5.4 Hz, 1H), 2.64 – 2.56 (m, 1H), 2.42 (q, *J* = 7.2 Hz, 2H), 2.35 (dd, *J* = 13.2, 4.4 Hz, 1H), 2.05 – 1.99 (m, 2H), 1.89 (p, *J* = 7.2 Hz, 2H).

^13^C NMR (101 MHz, DMSO) **δ** 173.34, 171.58, 171.53, 168.30, 163.03, 161.17, 146.64, 142.54, 134.27, 134.21, 133.73, 133.10, 131.26, 129.02, 121.53, 116.75, 116.09, 114.01, 60.23, 51.98, 40.42, 40.20, 39.99, 39.78, 39.57, 31.66, 25.82, 23.09, 21.23, 14.56.

ESI-MS: C_27_H_25_N_6_O_5_SCl 580,13 (calculated), 581,13 [M+H]^+^ (calculated), m/z = 581,05 [M+H]^+^ (found).

LC-MS: C_27_H_25_N_6_O_5_SCl 580,13 (calculated), 581,13 [M+H]^+^ (calculated), m/z = 581,10 [M+H]^+^ (found).

*
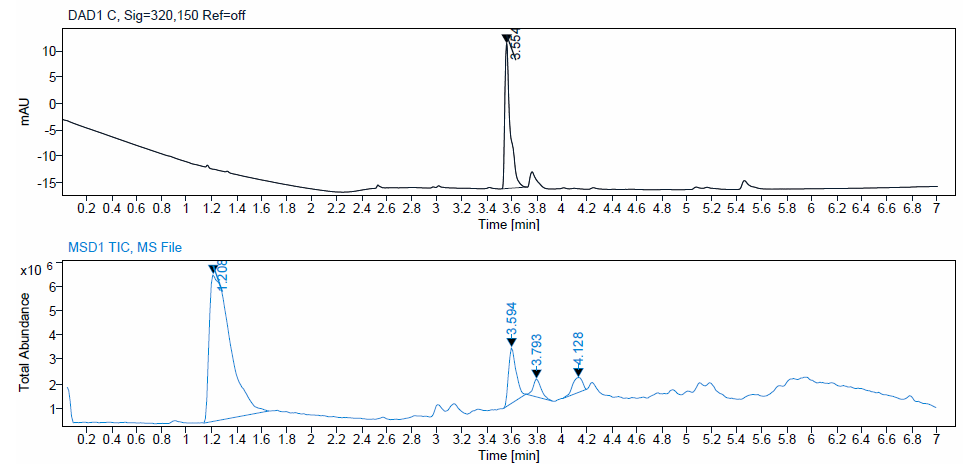
*

*
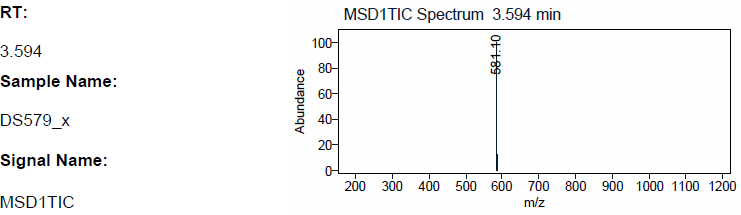
*

*
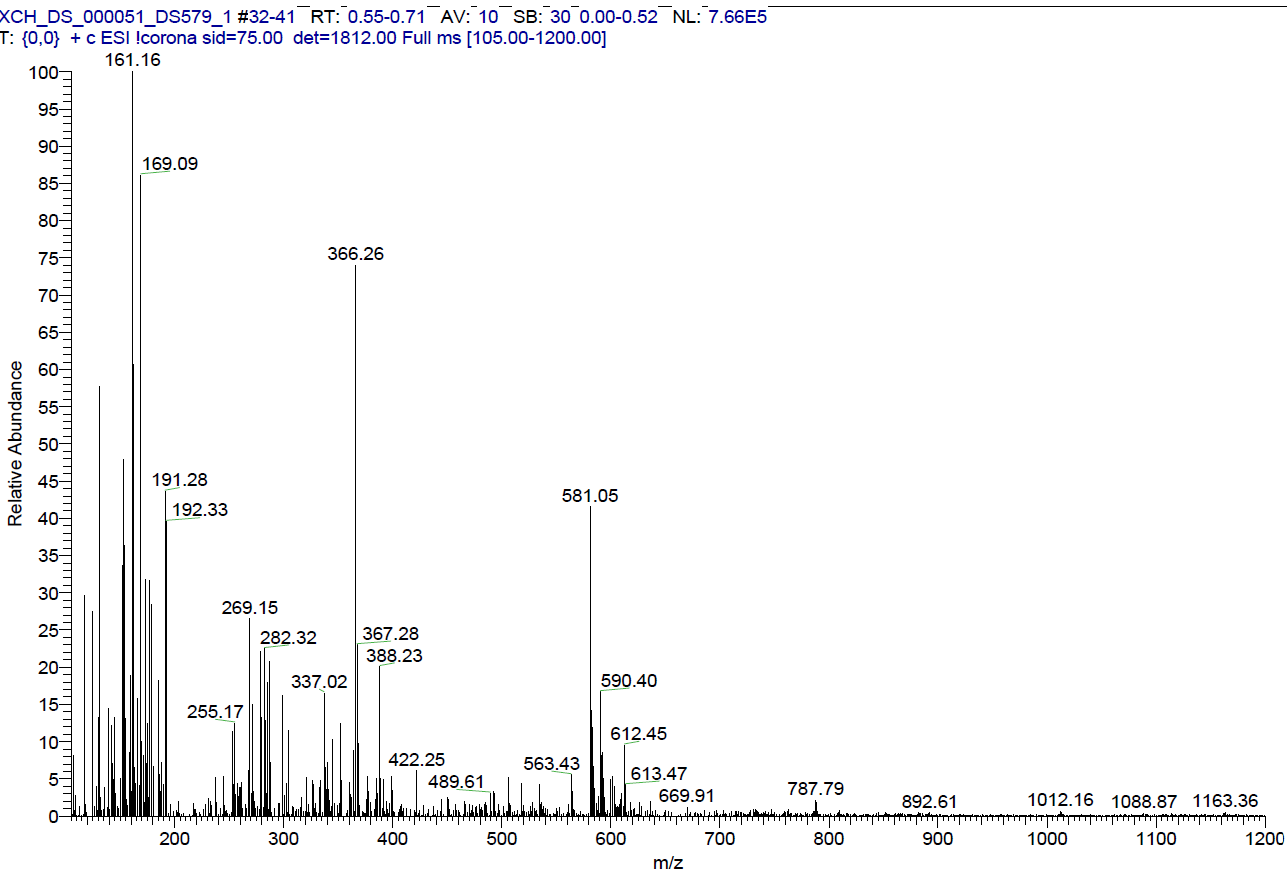
*

*PROTAC_C8:*

^1^H-NMR (400 MHz, DMSO-d_6_): **δ** 11.02 (s, 1H), 10.53 (s, 1H), 9.83 (s, 1H), 8.05 (t, *J* = 5.8 Hz, 1H), 7.82 (dd, *J* = 7.2, 1.9 Hz, 1H), 7.65 (dt, *J* = 11.8, 2.3 Hz, 1H), 7.58 – 7.45 (m, 3H), 7.41 – 7.31 (m, 2H), 6.84 – 6.72 (m, 1H), 5.14 (dd, *J* = 13.3, 5.1 Hz, 1H), 4.42 – 4.27 (m, 2H), 3.68 (q, *J* = 5.8 Hz, 3H), 3.52 (d, *J* = 8.5 Hz, 12H), 3.43 (s, 3H), 2.98 – 2.85 (m, 1H), 2.60 (q, *J* = 8.1 Hz, 3H), 2.33 (qd, *J* = 13.2, 4.4 Hz, 1H), 2.08 – 1.99 (m, 1H).

^13^C-NMR (400 MHz, DMSO-d_6_): **δ** 173.33, 171.53, 169.82, 168.30, 163.57, 163.18, 161.07, 146.39, 134.16, 134.11, 133.13, 129.14, 114.15, 113.48, 70.23, 70.20, 70.16, 70.07, 69.38, 67.04, 52.01, 40.39, 40.18, 39.97, 39.76, 39.54, 31.65, 23.11.

ESI-MS: C_32_H_35_N_6_O_8_SF 682,22 (calculated), 683,22 [M+H]^+^ (calculated), m/z = 683,18 [M+H]^+^ (found).

LC-MS: C_32_H_35_N_6_O_8_SF 682,22 (calculated), 683,22 [M+H]^+^ (calculated), m/z = 683,15 [M+H]^+^ (found).

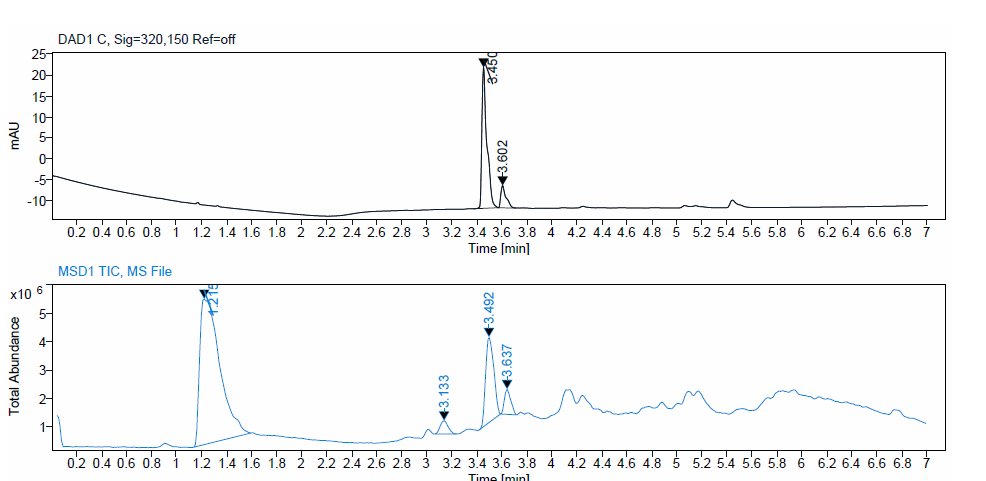


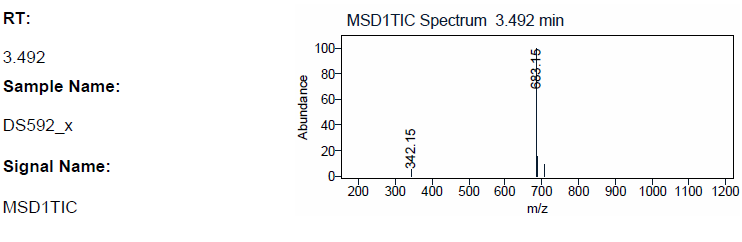


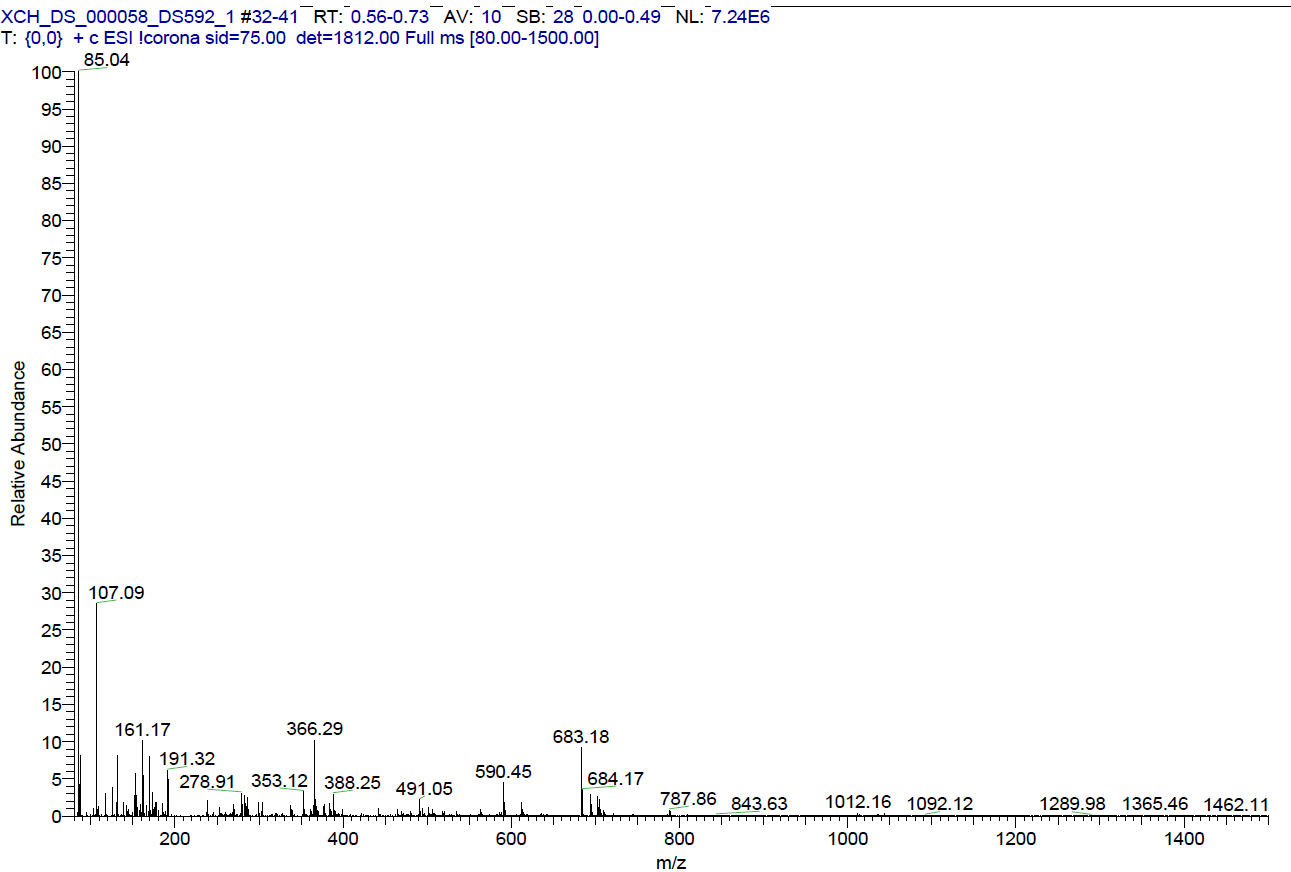


*PROTAC_C9:*

^1^H-NMR (400 MHz, DMSO-d_6_): **δ** 11.02 (s, 1H), 10.49 (s, 1H), 10.00 (s, 1H), 8.41 (t, *J* = 6.0 Hz, 1H), 7.86 (dt, *J* = 7.7, 3.9 Hz, 1H), 7.81 – 7.74 (m, 2H), 7.58 (s, 1H), 7.53 (q, *J* = 4.5 Hz, 2H), 7.37 – 7.31 (m, 2H), 5.15 (dd, *J* = 13.3, 5.1 Hz, 1H), 4.47 – 4.32 (m, 2H), 4.16 (d, *J* = 5.9 Hz, 2H), 2.97 – 2.87 (m, 1H), 2.61 (d, *J* = 16.6 Hz, 1H), 2.33 (dd, *J* = 12.8, 4.6 Hz, 1H), 2.10 – 2.01 (m, 1H).

^13^C-NMR (400 MHz, DMSO-d_6_): **δ** 173.30, 171.54, 168.39, 168.24, 163.29, 161.40, 146.01, 140.10, 133.95, 133.21, 129.24, 125.28, 119.17, 114.37, 40.41, 40.20, 39.99, 39.78, 39.57.

ESI-MS: C_25_H_21_N_6_O_5_SCl 552,10 (calculated), 553,10 [M+H]^+^ (calculated), m/z = 553,02 [M+H]^+^ (found).

LC-MS: C_25_H_21_N_6_O_5_SCl 552,10 (calculated), 553,10 [M+H]^+^ (calculated), m/z = 553,15 [M+H]^+^ (found).

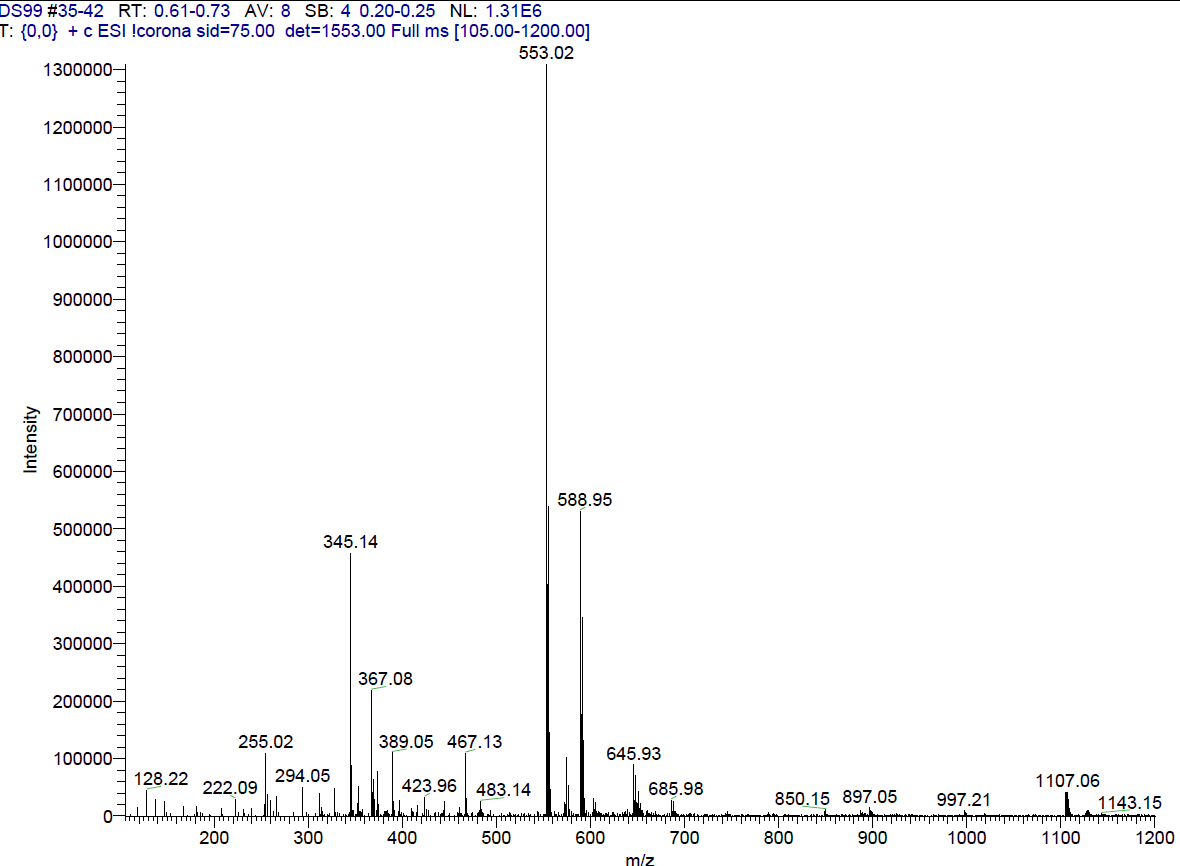


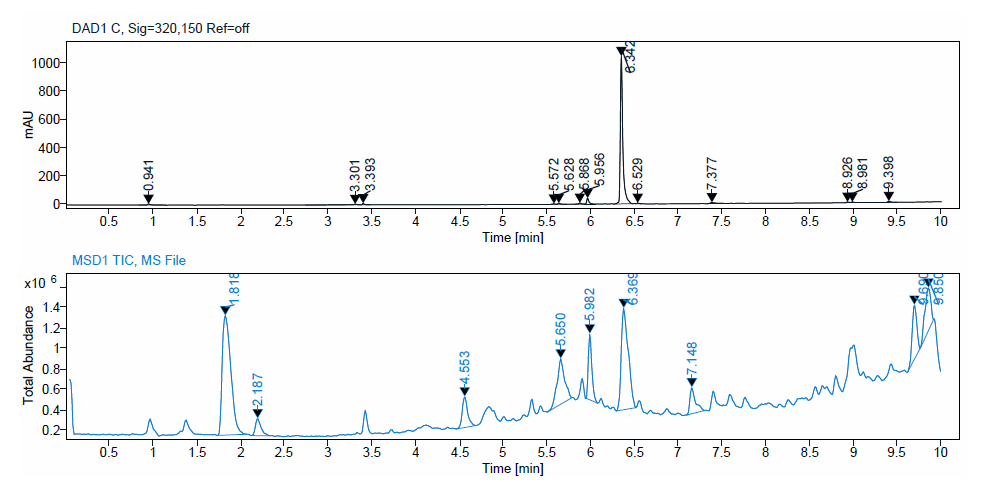


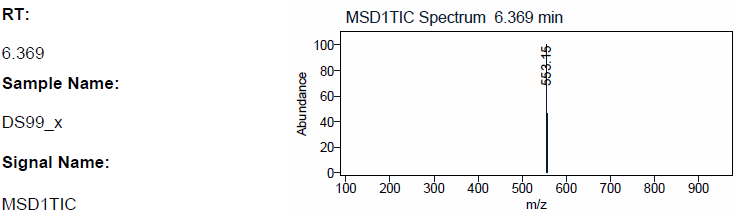


*Synthesis of ethyl 2-(3-acetylphenyl)thiazole-5-carboxylate* **(3)**

208 mg (1.27 mmol, 1.5 eq.) (3-acetylphenyl)boronic acid (**1**), 200 mg (0.85 mmol, 1 eq.) ethyl 2-bromothiazole-5-carboxylate (**2**), 50 mg (0.04 mmol, 0,05 eq.) Tetrakis(triphenylphosphine)palladium(0) and 350 mg (2.54 mmol, 3 eq.) Potassium carbonate were dissolved in 30 ml dioxane and the solution was heated to 102 °C for 16 h. The solution was allowed to cool to room temperature, placed on a 5 cm thick layer of celite and eluted twice with 50 ml ethyl acetate. The organic phases are combined and the solvent is concentrated under reduced pressure. Purification was carried out by RP FCC. The product was obtained as a brownish solid (150 mg, 64%).

^1^H-NMR (400 MHz, DMSO-d_6_): **δ** 8.55 (s, 1H), 8.51 (d, *J* = 1.9 Hz, 1H), 8.27 (dd, *J* = 7.8, 1.6 Hz, 1H), 8.16 – 8.10 (m, 1H), 7.71 (t, *J* = 7.8 Hz, 1H), 4.40 – 4.29 (m, 2H), 2.67 (s, 3H), 1.39 – 1.28 (m, 3H)

^13^C-NMR (400 MHz, DMSO-d_6_): **δ** 197.32, 171.28, 160.52, 149.18, 137.66, 132.55, 131.04, 130.94, 130.03, 129.22, 125.89, 61.65, 40.15, 39.99, 39.94, 39.78, 39.73, 39.52, 39.31, 39.10, 38.90, 26.88, 14.10.

*Synthesis of 2-(3-acetylphenyl)thiazole-5-carboxylic acid* (**4**):

150 mg (0.545 mmol, 1 eq.) ethyl 2-(3-acetylphenyl)thiazole-5-carboxylate **(3)** was dissolved in 5 ml methanol and 15 ml 4N NaOH was added. The solution was heated to 40 °C for 3 h. The reaction was monitored using TLC. The reaction was then purified by RF FCC and a brownish solid was obtained (121.4 mg, 90%).

^1^H-NMR (400 MHz, DMSO-d_6_): **δ** 13.69 (s, 1H), 8.54 – 8.44 (m, 2H), 8.31 – 8.24 (m, 1H), 8.14 (dt, *J* = 7.9, 1.4 Hz, 1H), 7.72 (t, *J* = 7.8 Hz, 1H), 2.68 (s, 3H).

^13^C-NMR (400 MHz, DMSO-d_6_): **δ** 197.34, 170.90, 161.94, 148.81, 137.64, 132.73, 130.98, 130.83, 130.80, 129.99, 125.76, 40.14, 39.93, 39.72, 39.51, 39.30, 39.09, 38.89, 26.87.

*2,5-dioxopyrrolidin-1-yl 2-(3-acetylphenyl)thiazole-5-carboxylate (****5****):*

105 mg (0.42 mmol, 1 eq.) 2-(3-acetylphenyl)thiazole-5-carboxylic acid (**4**), 65 mg (0.55 mmol, 1.3 eq.) 1-ethyl-3-(3-dimethylaminopropyl)carbodiimid-hydrochlorid and 106 mg (0.55 mmol, 1.3 eq.) N-hydroxysuccinimide were dissolved in 2 ml DMF and stirred for 3 h at RT. The reaction was monitored using TLC. After the reaction is complete, 15 ml dist. H2O was added. The resulting precipitate was filtered and dried under reduced pressure, resulting in a white solid (124.8 mg, 85%).

^1^H-NMR (400 MHz, DMSO-d_6_): **δ** 8.95 (s, 1H), 8.59 (t, J = 1.8 Hz, 1H), 8.36 (ddd, J = 7.8, 1.9, 1.1 Hz, 1H), 8.19 (dt, J = 7.9, 1.4 Hz, 1H), 7.76 (t, J = 7.8 Hz, 1H), 2.92 (s, 4H), 2.69 (s, 3H).
^13^C-NMR (400 MHz, DMSO-d_6_): **δ** 197.77, 174.77, 170.55, 157.19, 153.22, 138.23, 132.54, 132.16, 132.12, 132.02, 130.66, 126.86, 122.75, 40.42, 40.21, 40.00, 39.79, 39.57, 27.40,

*Synthesis of N^2^-(2-(3-acetylphenyl)thiazole-5-carbonyl)-N^6^-(tert-butoxycarbonyl)lysine (****7****):*

60 mg (0.174 mmol, 1 eq.) 2,5-dioxopyrrolidin-1-yl 2-(3-acetylphenyl)thiazole-5-carboxylate (**5**), 47 mg (0.174 mmol, 1 eq.) N^6^-(tert-butoxycarbonyl)lysine and 52 mg (0.07 ml, 0.4 mmol, 2.3 eq.) N,N-Diisopropylethylamine (DIPEA) were dissolved in 1.8 ml ACN and 0.2 ml DMF. The reaction was stirred for 2 h at RT and monitored using TLC. Subsequently, the reaction then purified by RF FCC and a yellowish solid was obtained (70 mg, 84%).

^1^H-NMR (400 MHz, DMSO-d_6_): **δ** 12.78 (s, 1H), 8.92 (d, J = 7.7 Hz, 1H), 8.62 (s, 1H), 8.49 (d, J = 1.8 Hz, 1H), 8.24 (dt, J = 7.9, 1.5 Hz, 1H), 8.15 – 8.12 (m, 1H), 7.70 (t, J = 7.8 Hz, 1H), 6.79 (t, J = 5.7 Hz, 1H), 4.34 (td, J = 8.9, 5.0 Hz, 1H), 2.91 (t, J = 6.5 Hz, 2H), 2.67 (s, 3H), 1.89 – 1.69 (m, 2H), 1.36 (s, 13H).

^13^C-NMR (400 MHz, DMSO-d_6_): **δ** 197.90, 173.87, 169.73, 163.59, 160.27, 156.05, 144.95, 138.11, 136.06, 133.40, 131.47, 131.36, 131.01, 130.46, 126.06, 77.83, 77.80, 53.14, 40.26, 40.20, 39.99, 39.78, 39.57, 30.87, 29.57, 27.37, 27.34, 23.63.

*Synthesis of tert-butyl-(5-(2-(3-acetylphenyl)thiazole-5-carboxamido)-6-((2-methoxyethyl)amino)-6-oxohexyl)carbamate (****USP39_B2****):*

60 mg (0.13 mmol, 1 eq.) N^2^-(2-(3-acetylphenyl)thiazole-5-carbonyl)-N^6^-(tert-butoxycarbonyl)lysine (**7**), 11 mg (0.14 mmol, 1 eq.) 2-methoxyethan-1-amine, 64 mg (0.167 mmol, 1.3 eq.) O-(7-Azabenzotriazol-1-yl)-N,N,N′,N′-tetramethyluronium-hexafluorphosphat (HATU) and 50 mg (0.07 ml, 0.38 mmol, 3 eq.) DIPEA were dissolved in 1.5 ml DMF and stirred overnight at RT. The reaction was purified by RF FCC and a yellow solid was obtained (35.4 mg, 53%).

^1^H-NMR (300 MHz, DMSO-d_6_): **δ** 8.85 (dd, *J* = 40.3, 7.8 Hz, 1H), 8.64 (d, *J* = 9.0 Hz, 1H), 8.50 (q, *J* = 1.6 Hz, 1H), 8.25 (dq, *J* = 7.8, 1.6 Hz, 1H), 8.18 – 8.06 (m, 2H), 7.71 (dd, *J* = 8.2, 7.3 Hz, 1H), 6.76 (s, 1H), 4.46 – 4.31 (m, 1H), 3.36 (t, *J* = 5.4 Hz, 5H), 3.26 (s, 4H), 2.91 (d, *J* = 6.6 Hz, 2H), 2.68 (s, 3H), 1.81 – 1.59 (m, 2H), 1.36 (d, *J* = 0.9 Hz, 13H).

LC/MS: C_26_H_36_N_4_O_6_S 532,24 (calc.), 555,22 [M+Na]^+^ (calc.), m/z = 555,15 [M+Na]^+^ (found).

ESI-MS: C_26_H_36_N_4_O_6_S 532,24 (calc.), 531,24 [M-H]^-^ (calc.), 577,20 [M+2Na]^2+^ (calc.), m/z = 531,19 [M-H]^-^ (found), 577,26 [M+2Na]^2+^ (found).

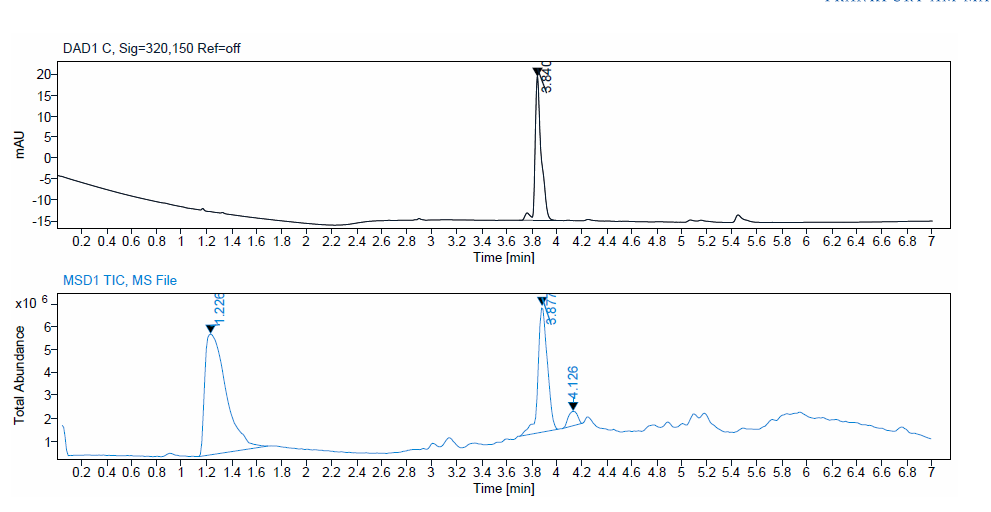


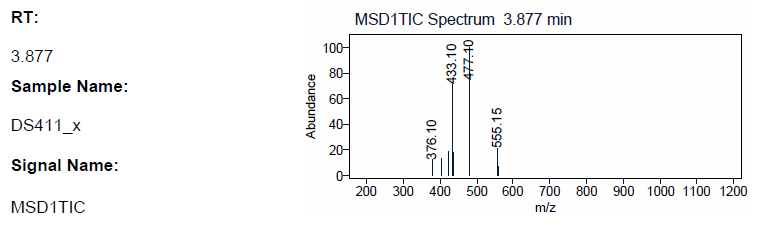


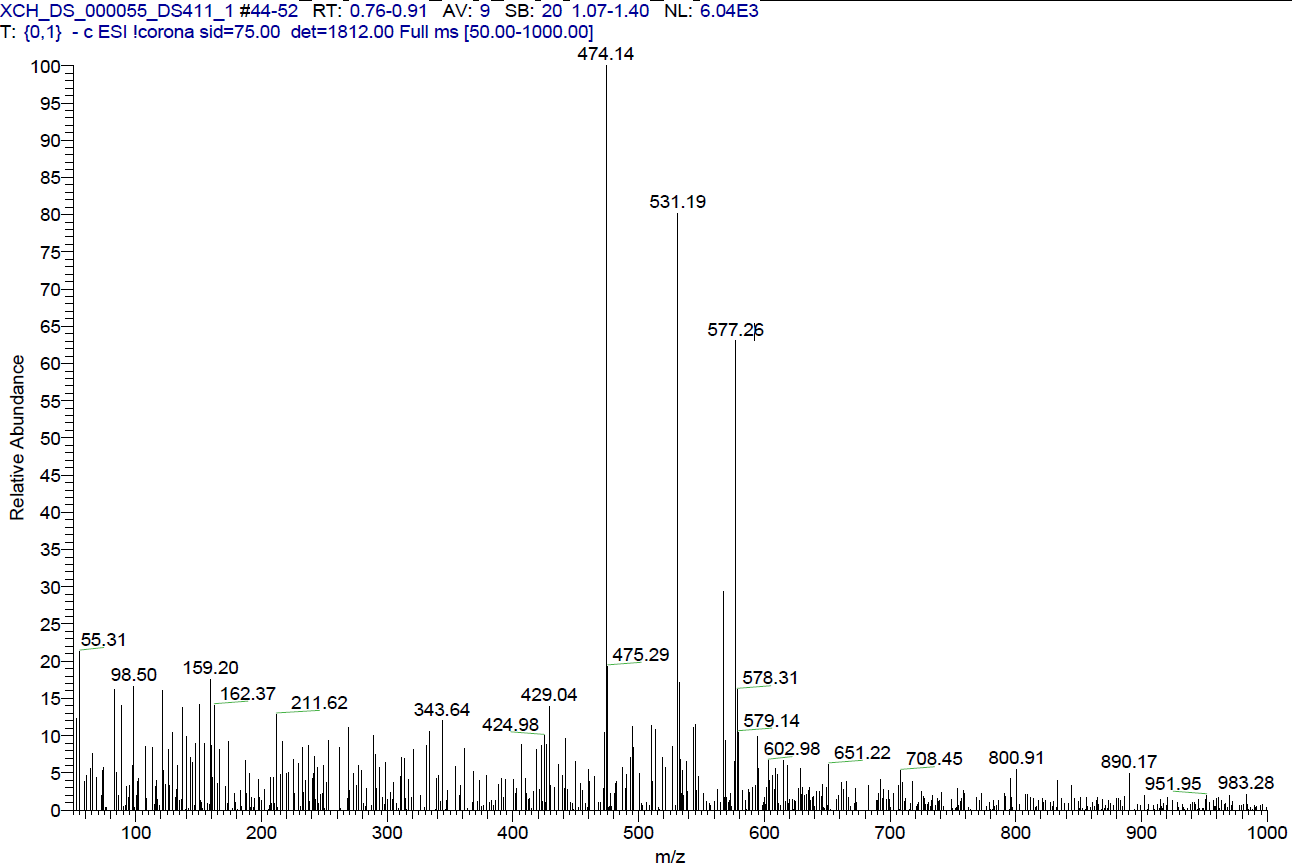


*Synthese of tert-butyl-(19-(2-(3-acetylphenyl)thiazole-5-carboxamido)-3-(4-hydroxy-2-((4-(4-methylthiazol-5-yl)benzyl)carbamoyl)pyrrolidine-1-carbonyl)-2,2-dimethyl-5,18-dioxo-8,11,14-trioxa-4,17-diazatricosan-23-yl)carbamate (****PROTAC_V4****):*

51.8 mg (0.110 mmol; 1.00 eq) N^2^-(2-(3-acetylphenyl)thiazole-5-carbonyl)-N^6^-(tert-butoxycarbonyl)lysine (**7**), 71.7 mg (0.113 mmol; 1.03 eq) (4R)-1-[(2S)-2-[3-[2-[2-(2-Aminoethoxy)ethoxy]ethoxy]propanoylamino]-3,3-dimethylbutanoyl]-4-hydroxy-N-[[4-(4-methylthiazol-5-yl)phenyl]methyl]pyrrolidine-2-carboxamide (NH_2_-PEG(3)-VHL-linker) and 43.8 mg (0.115 mmol; 1.05 eq) of HATU were stirred in 1.5 ml of DMF and 0.032 ml (0.230 mmol; 2.10 eq) of TEA overnight at RT. The solution was purified by RP FCC, and the mixed fraction of product and reactant was separated by NP FCC of 100% CH to 1:1 CH/acetone. The product was obtained as 24.4 mg of a colorless resin with a yield of 20.24%.

^1^H-NMR (600 MHz, DMSO-d_6_): **δ** 0.889 (s, H_A_, 9 H); 1.305-1.353 (m, H_A+Linker_, 13 H); 1.617-1.686 (m, H_Linker_, 2 H); 1.842-1.884 (m, H_Rest_, 1 H); 1.978-2.010 (m, H_Rest_, 1 H); 2.286-2.329 (m, H_Rest_, 1 H); 2.398 (s, H_PEG_, 2 H); 2.461 (s, H_B_, 3 H); 2.622 (s, H_C_, 3 H); 2.846-2.855 (m, H_Linker_, 2 H); 3.151-3.235 (m, H_Rest_, 2 H); 3.384 (t, ^3^*J* = 5.7 Hz, H_PEG_, 2 H); 3.40-3.452 (m, H_PEG_, 8 H); 3.506-3.642 (m, H_PEG_, 4 H); 4.197 (dd, *J* = 16.0, *J* = 5.3 Hz, H_Rest_, 1 H); 4.311 (s, H_Rest_, 1 H); 4.353-4.402 (m, H_D+Rest_, 3 H); 4.517 (d, ^3^*J* = 9.4 Hz, H_Rest_, 1 H); 5.080 (d, ^3^*J* = 3.0 Hz, H_Rest_, 1 H); 6.706 (s, H_N_, 1 H); 7.331-7.380 (m, H_O+P_, 2 H); 7.660 (t, ^3^*J* = 7.8 Hz, H_Q_, 1 H); 7.869 (d, ^3^*J* = 9.5 Hz, H_R_, 1 H); 8.056-8.068 (m, H_S+T_, 2 H); 8.190 (d, ^3^*J* = 7.7 Hz; H_U_, 1 H); 8.438 (s, H_V_, 1 H); 8.747 (t, ^3^*J* = 5.6 Hz, H_W_, 1 H); 8.604 (s, H_X_, 1 H); 8.747 (d, ^3^*J* = 7.9 Hz, H_Y_, 1 H); 8.934 (s, H_Z_, 1 H).

^13^C-NMR (600 MHz, DMSO-d_6_): **δ** 15.891; 23.013; 26.291; 26.844; 28.215; 29.171; 31.511; 35.312; 35.630; 37.904; 38.594; 41.631; 53.343; 56.271; 56.320; 58.677; 66.913; 68.840; 68.920; 69.440; 69.556; 69.680; 69.706; 77.274; 125.516; 127.407; 128.611; 129.628; 129.940; 130.465; 130.815; 131.129; 132.959; 135.832; 137.624; 139.456; 144.472; 147.677; 151.394; 155.507; 159.51; 169.060; 169.500; 169.889; 171.450; 171.867; 197.373.

LC-ESI-MS: C_54_H_74_N_8_O_12_S_2_ 1090,49 (calculated), 1113,32 [M+Na]^+^ (calculated), m/z = 1113,45 [M+Na]^+^ (found).


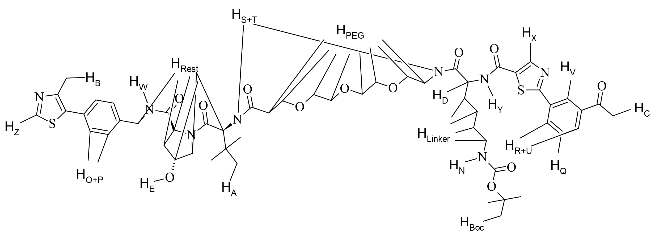

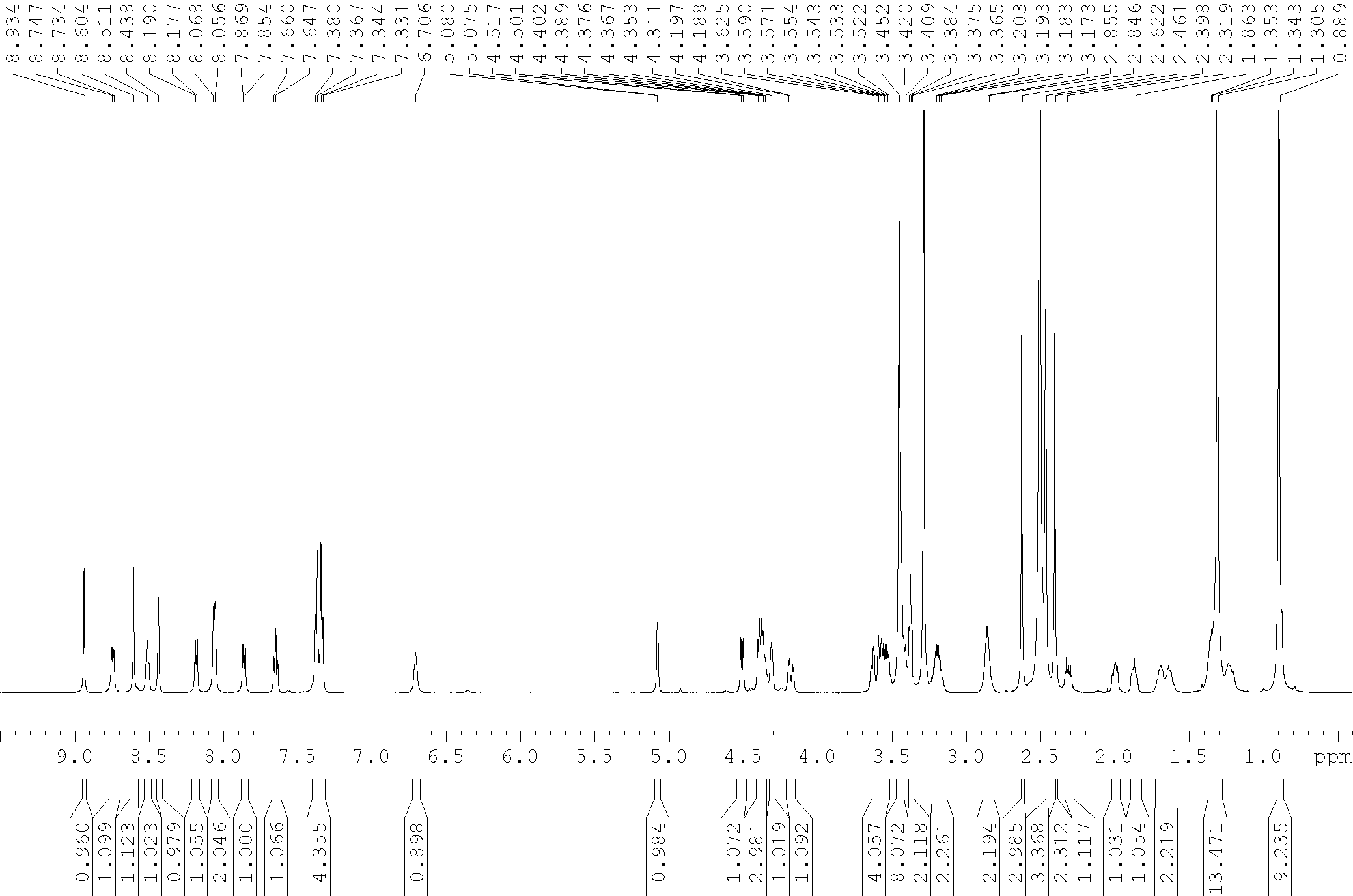


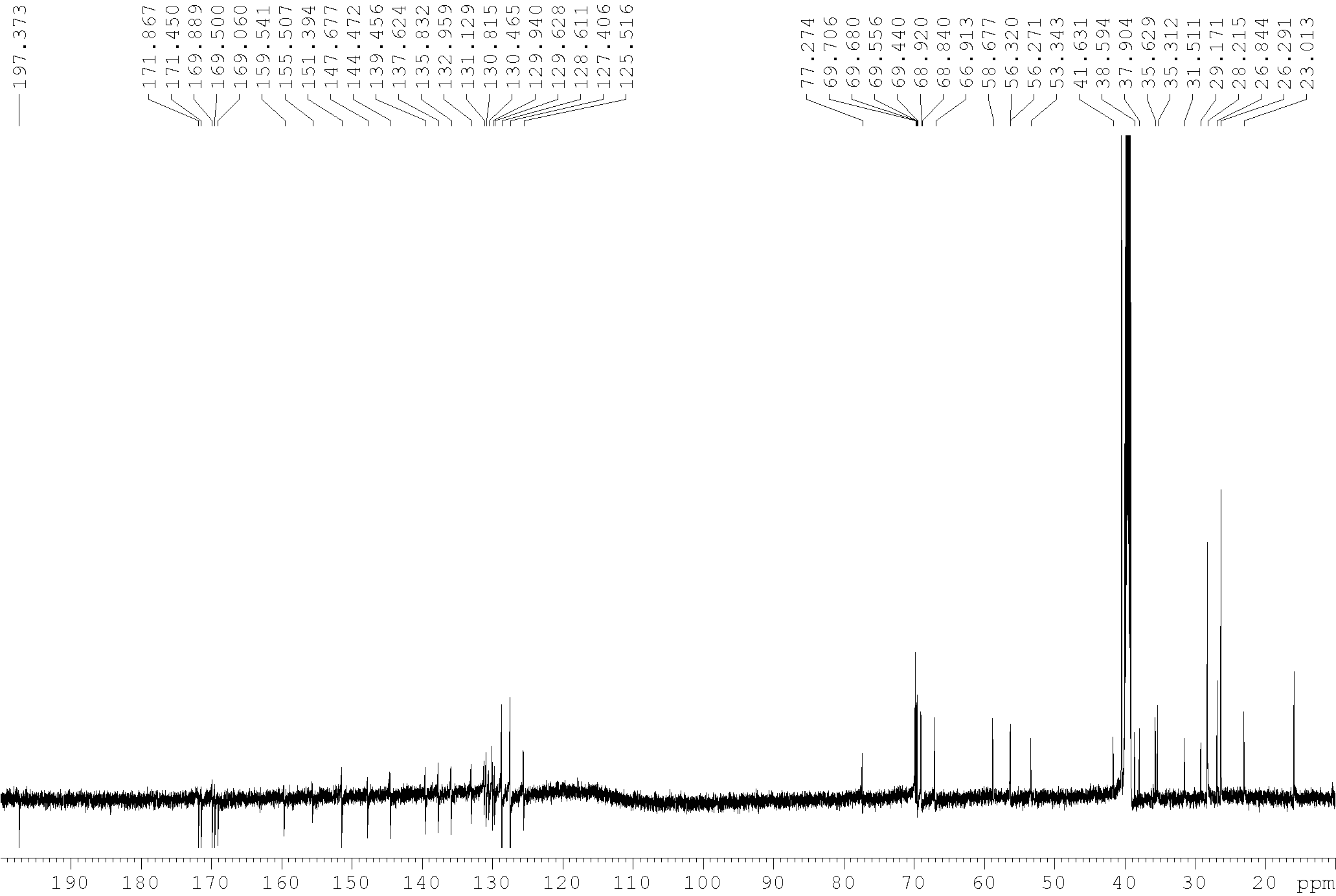


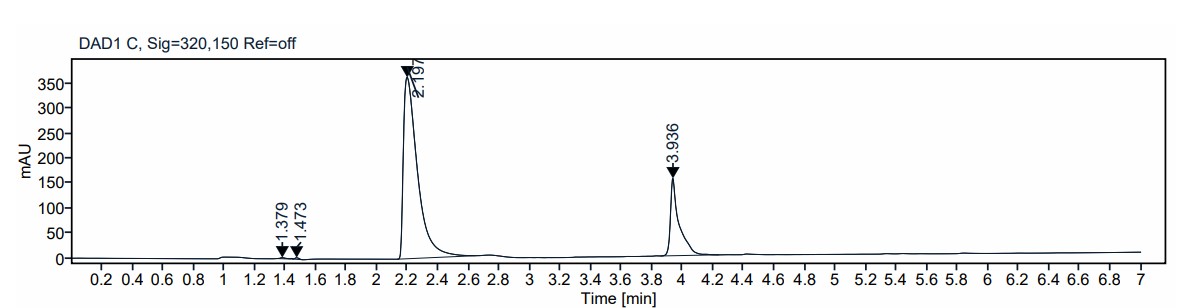


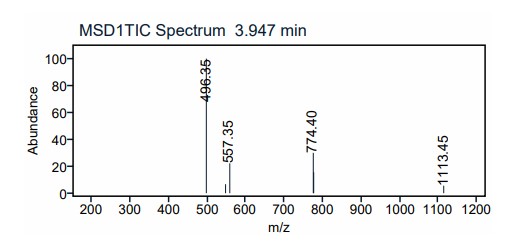


*General synthesis of ureas PROTACs:*

Note: Triphosgene should be handled strictly under an inert atmosphere in a fume hood, as it decomposes to toxic phosgene upon contact with moisture or heat. Appropriate personal protective equipment (lab coat, nitrile gloves, safety glasses) should be worn at all times.

1.00 eq. of CRBN-linker-NH_2_ or VHL-linker-NH_2_ was suspended in THF under a nitrogen atmosphere, 3.00 eq. of NEt_3_ was added and the solution was cooled to 0 °C. Subsequently, 0.40 eq. triphosgene was dissolved in THF under a nitrogen atmosphere and added. After 1 h of stirring at RT, a solution of 1.00 eq. 2-amino-5-nitrothiazole and 1.10 eq. NEt_3_ was prepared in THF under a nitrogen atmosphere and added to the first solution. After completion of the reaction, residual triphosgene was quenched by dropwise addition of anhydrous methanol under cooling until gas evolution ceases.

Synthese von Synthese von N-[2-(2,6-Dioxo-3-piperidyl)-1-oxo-isoindolin-4-yl]-9-[(5-nitrothiazol-2-yl)-carb-amoylamino]nonanamid (**PROTAC_C10**):

151.3 mg (0.37 mmol; 1.00 eq) of 9-amino-N-[2-(2,6-dioxo-3-piperidyl)-1-oxo-isoindolin-4-yl]nonanamide (CRBN-linker(C9)-NH_2)_ was reacted in 10 ml of THF according to general synthesis of ureas PROTACs. The solution was stirred for two days at RT, filtered and all volatiles were discarded under reduced pressure. Purification was carried out by RP column chromatography. The product was obtained as yellow mucilage (9.2 mg, 4,24%).

^1^H-NMR (400 MHz, DMSO-d6): **δ** 1.234-1.285 (m, HA-D, 8 H), 1.455-1.489 (m, HE, 2 H); 1.583-1.599 (m, HF, 2 H); 2.011-2.038 (m, HG, 1 H); 2.328-2.365 (m, HH und HI, 3 H); 2.570-2.626 (m, HJ, 1 H); 2.917-2.991 (m, HK, 1 H); 3.139-3.188 (m, HL, 2 H); 4.345 (d, 3*J* = 17.4 Hz, HN, 1H); 4.410 (d, 3*J* = 17.6 Hz, HO, 1H); 5.165 (dd, 3*J* = 13.4 Hz, 4*J* = 5.3 Hz, HP, 1 H); 7.459-7.513 (m, HT und HU, 2 H); 7.815 (d, 3 *J* = 6.8 Hz, HV, 1 H); 8.185 (s, HW, 1 H); 9.387 (s, HX, 1 H); 9.757 (s, HY, 1 H); 11.017 (s, HZ, 1 H).

^13^C-NMR (400 MHz, DMSO-d6): **δ** 9.527; 23.108; 25.532; 26.580; 28.641; 28.947; 29.078; 29.148; 31.671; 36.262; 46.045; 46.930; 51.987; 115.027; 119.425; 125.692; 129.077; 133.127; 134.278; 156.409; 156.764; 164.035; 168.298; 171.542; 171.837; 173.329.

ESI-MS: C_26_H_31_N_7_O_7_S 585,20 (calculated), 584,20 [M-H]^-^ (calculated), m/z = 584,14 [M-H]^-^ (found).

LC-MS: C_26_H_31_N_7_O_7_S 585,20 (calculated), 586,20 [M+H]^+^ (calculated), m/z = 586,15 [M+H]^+^ (found).

Synthese von 1-(16-(tert-butyl)-1-((5-nitrothiazol-2-yl)amino)-1,14-dioxo-5,8,11-trioxa-2,15-diazaheptadecan-17-oyl)-4-hydroxy-N-(4-(4-methylthiazol-5-yl)benzyl)pyrrolidine-2-carboxamide (**PROTAC_V8**):

50 mg (0.08 mmol; 1.00 eq) of 1-(1-amino-14-(tert-butyl)-12-oxo-3,6,9-trioxa-13-azapentadecan-15-oyl)-4-hydroxy-N-(4-(4-methylthiazol-5-yl)benzyl)pyrrolidine-2-carboxamide (VHL-linker(PEG(3))-NH_2)_ was reacted in 10 ml of THF according to general synthesis of ureas PROTACs. The solution was stirred overnight at RT, filtered and all volatiles were discarded under reduced pressure. Purification was carried out by RP column chromatography. The product was obtained as yellow solid (11.1 mg, 17,5%).

^1^H-NMR (400 MHz, DMSO-d_6_): **δ** 8.98 (s, 1H), 8.57 (t, *J* = 6.1 Hz, 1H), 7.91 (d, *J* = 9.3 Hz, 1H), 7.49 – 7.35 (m, 5H), 5.15 (d, *J* = 3.5 Hz, 1H), 4.55 (d, *J* = 9.4 Hz, 1H), 4.47 – 4.39 (m, 2H), 4.36 (s, 1H), 4.22 (dd, *J* = 15.9, 5.5 Hz, 1H), 3.71 – 3.46 (m, 20H), 2.44 (s, 4H), 2.35 (dt, *J* = 14.7, 6.1 Hz, 1H), 2.09 – 1.98 (m, 1H), 1.95 – 1.85 (m, 1H), 0.93 (s, 10H).

^13^C-NMR (400 MHz, DMSO-d_6_): **δ** 172.43, 170.49, 170.00, 151.94, 148.18, 139.95, 131.64, 130.10, 129.18, 129.11, 127.90, 70.21, 70.18, 70.07, 69.95, 69.34, 67.40, 59.20, 56.86, 40.35, 40.14, 39.93, 39.72, 39.50, 26.77, 16.39.

ESI-MS: C_35_H_48_N_8_O_10_S_2_ 804,29 (calculated), 805,29 [M-H]^+^ (calculated), m/z = 805,38 [M-H]^+^ (found).

LC-MS: C_35_H_48_N_8_O_10_S_2_ 804,29 (calculated), 805,29 [M-H]^+^ (calculated), m/z = 805,30 [M+H]^+^ (found).

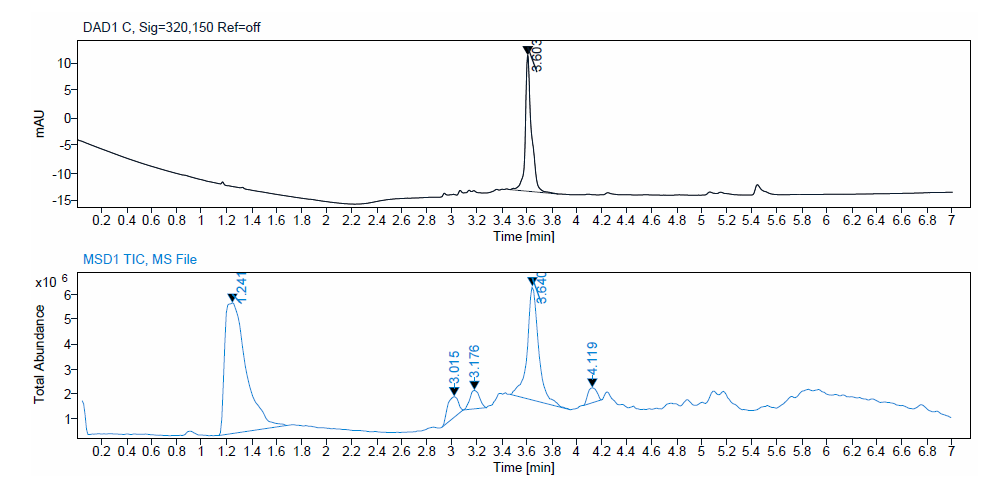


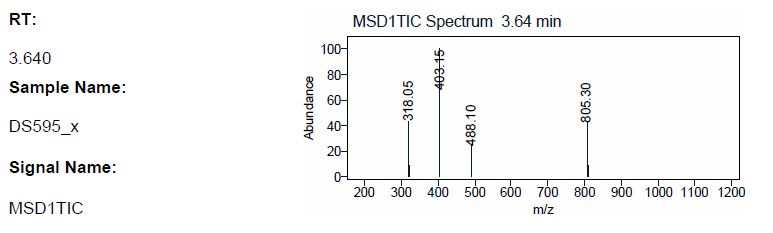


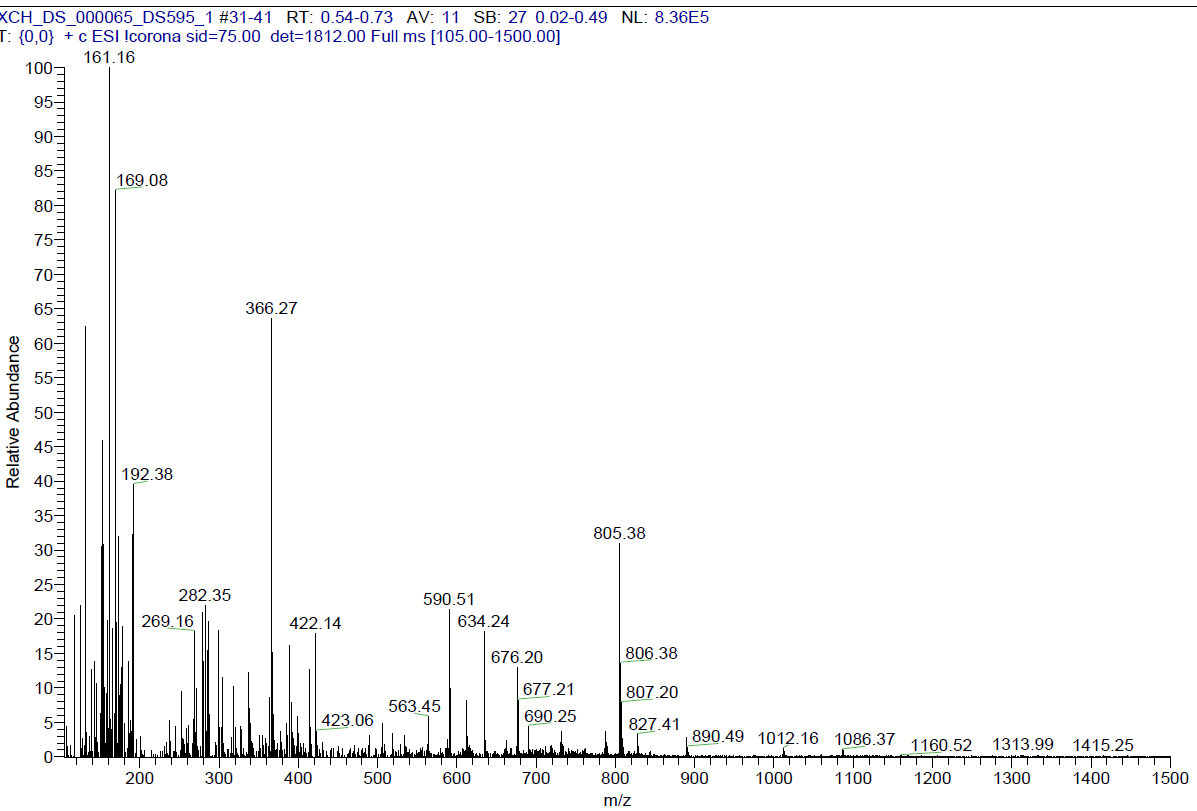


1. Wang, J.; Heinz, M.; Han, K.; Shah, V. J.; Hasselbeck, S.; Schwalm, M. P.; Rathore, R.; Hummer, G.; Zhou, J.; Dikic, I., Thalidomide derivatives degrade BCL-2 by reprogramming the binding surface of CRBN. *Clinical cancer research* **2024,** *5* (5).

2. Gama-Brambila, R. A.; Chen, J.; Zhou, J.; Tascher, G.; Münch, C.; Cheng, X., A PROTAC targets splicing factor 3B1. *Cell chemical biology* **2021,** *28* (11), 1616-1627. e8.

3. Abramson, J.; Adler, J.; Dunger, J.; Evans, R.; Green, T.; Pritzel, A.; Ronneberger, O.; Willmore, L.; Ballard, A. J.; Bambrick, J.; Bodenstein, S. W.; Evans, D. A.; Hung, C.-C.; O’Neill, M.; Reiman, D.; Tunyasuvunakool, K.; Wu, Z.; Žemgulytė, A.; Arvaniti, E.; Beattie, C.; Bertolli, O.; Bridgland, A.; Cherepanov, A.; Congreve, M.; Cowen-Rivers, A. I.; Cowie, A.; Figurnov, M.; Fuchs, F. B.; Gladman, H.; Jain, R.; Khan, Y. A.; Low, C. M. R.; Perlin, K.; Potapenko, A.; Savy, P.; Singh, S.; Stecula, A.; Thillaisundaram, A.; Tong, C.; Yakneen, S.; Zhong, E. D.; Zielinski, M.; Žídek, A.; Bapst, V.; Kohli, P.; Jaderberg, M.; Hassabis, D.; Jumper, J. M., Accurate structure prediction of biomolecular interactions with AlphaFold 3. *Nature* **2024,** *630* (8016), 493-500.

4. Madhavi Sastry, G.; Adzhigirey, M.; Day, T.; Annabhimoju, R.; Sherman, W., Protein and ligand preparation: parameters, protocols, and influence on virtual screening enrichments. *Journal of Computer-Aided Molecular Design* **2013,** *27* (3), 221-234.

5. Halgren, T. A., Identifying and Characterizing Binding Sites and Assessing Druggability. *Journal of Chemical Information and Modeling* **2009,** *49* (2), 377-389.

6. Friesner, R. A.; Banks, J. L.; Murphy, R. B.; Halgren, T. A.; Klicic, J. J.; Mainz, D. T.; Repasky, M. P.; Knoll, E. H.; Shelley, M.; Perry, J. K.; Shaw, D. E.; Francis, P.; Shenkin, P. S., Glide:  A New Approach for Rapid, Accurate Docking and Scoring. 1. Method and Assessment of Docking Accuracy. *Journal of Medicinal Chemistry* **2004,** *47* (7), 1739-1749.

7. Friesner, R. A.; Murphy, R. B.; Repasky, M. P.; Frye, L. L.; Greenwood, J. R.; Halgren, T. A.; Sanschagrin, P. C.; Mainz, D. T., Extra Precision Glide:  Docking and Scoring Incorporating a Model of Hydrophobic Enclosure for Protein−Ligand Complexes. *Journal of Medicinal Chemistry* **2006,** *49* (21), 6177-6196.

8. Hughes, C. S.; Moggridge, S.; Müller, T.; Sorensen, P. H.; Morin, G. B.; Krijgsveld, J., Single-pot, solid-phase-enhanced sample preparation for proteomics experiments. *Nature Protocols* **2019,** *14* (1), 68-85.

9. Messner, C. B.; Demichev, V.; Wendisch, D.; Michalick, L.; White, M.; Freiwald, A.; Textoris-Taube, K.; Vernardis, S. I.; Egger, A.-S.; Kreidl, M.; Ludwig, D.; Kilian, C.; Agostini, F.; Zelezniak, A.; Thibeault, C.; Pfeiffer, M.; Hippenstiel, S.; Hocke, A.; von Kalle, C.; Campbell, A.; Hayward, C.; Porteous, D. J.; Marioni, R. E.; Langenberg, C.; Lilley, K. S.; Kuebler, W. M.; Mülleder, M.; Drosten, C.; Suttorp, N.; Witzenrath, M.; Kurth, F.; Sander, L. E.; Ralser, M., Ultra-High-Throughput Clinical Proteomics Reveals Classifiers of COVID-19 Infection. *Cell Systems* **2020,** *11* (1), 11-24.e4.

10. Demichev, V.; Messner, C. B.; Vernardis, S. I.; Lilley, K. S.; Ralser, M., DIA-NN: neural networks and interference correction enable deep proteome coverage in high throughput. *Nature Methods* **2020,** *17* (1), 41-44.

11. Tyanova, S.; Temu, T.; Sinitcyn, P.; Carlson, A.; Hein, M. Y.; Geiger, T.; Mann, M.; Cox, J., The Perseus computational platform for comprehensive analysis of (prote)omics data. *Nat Methods* **2016,** *13* (9), 731-40.
